# Unraveling differences in aluminyl and carbene coordination chemistry: bonding in gold complexes and reactivity with carbon dioxide†

**DOI:** 10.1039/d2sc00630h

**Published:** 2022-03-31

**Authors:** Diego Sorbelli, Leonardo Belpassi, Paola Belanzoni

**Affiliations:** Department of Chemistry, Biology and Biotechnology, University of Perugia Via Elce di Sotto, 8 – 06123 Perugia Italy diegosorbelli00@gmail.com paola.belanzoni@unipg.it; CNR Institute of Chemical Science and Technologies “Giulio Natta” (CNR-SCITEC) Via Elce di Sotto, 8 – 06123 Perugia Italy leonardo.belpassi@cnr.it

## Abstract

The electronic properties of aluminyl anions have been reported to be strictly related to those of carbenes, which are well-known to be easily tunable *via* selected structural modifications imposed on their backbone. Since peculiar reactivity of gold-aluminyl complexes towards carbon dioxide has been reported, leading to insertion of CO_2_ into the Au–Al bond, in this work the electronic structure and reactivity of Au–Al complexes with different aluminyl scaffolds have been systematically studied and compared to carbene analogues. The analyses reveal that, instead, aluminyls and carbenes display a very different behavior when bound to gold, with the aluminyls forming an electron-sharing and weakly polarized Au–Al bond, which turns out to be poorly modulated by structural modifications of the ligand. The reactivity of gold–aluminyl complexes towards CO_2_ shows, both qualitatively and quantitatively, similar reaction mechanisms, reflecting the scarce tunability of their electronic structure and bond nature. This work provides further insights and perspectives on the properties of the aluminyl anions and their behavior as coordination ligands.

## Abstract 2

Aluminyls and carbenes as coordination ligands, although sharing similar electronic properties, reveal fundamental differences in their tunability, bonding to gold and reactivity of their complexes with carbon dioxide.

## Graphical abstract

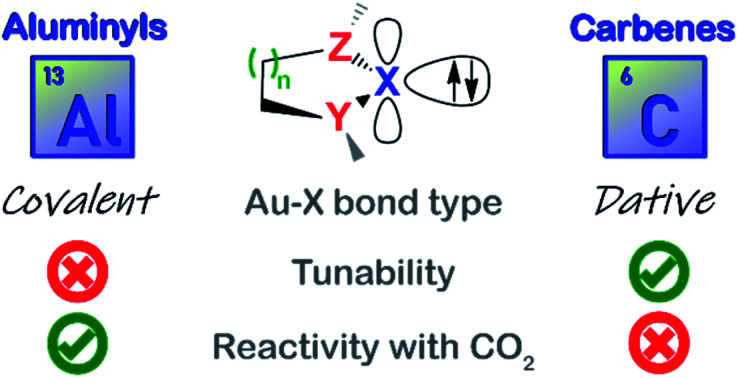

## Introduction

**Scheme 1:**
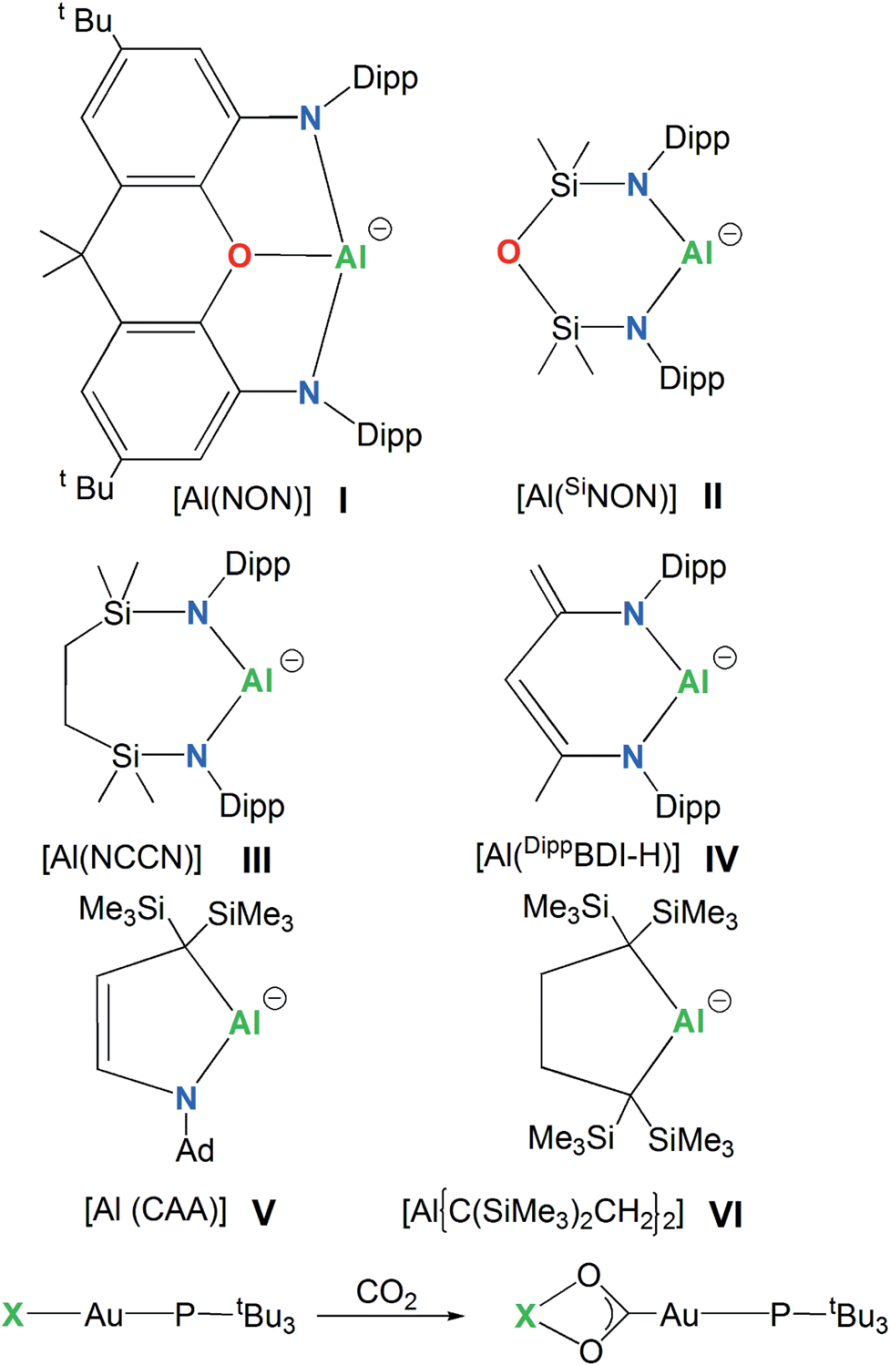
Aluminyl anions studied here (species I–VI) and the CO_2_ insertion reaction in their corresponding model gold complexes [^*t*^Bu_3_PAuX] (X = I, II, III, IV, V and VI).

In 2018, the isolation of the aluminum(i) compound K_2_[Al(NON)]_2_, consisting of a three-coordinated Al supported by a dianionic, di(amido)dimethylxanthene-based [NON]^2−^ (NON = 4,5-bis(2,6-diisopropylanilido)-2,7-di-*tert*-butyl-9,9-dimethylxanthene) ligand, sanctioned a novel class of anionic Al(i) compounds, named the aluminyl anions (species I, Scheme 1).^1,2^ Shortly after this discovery, the family of aluminyl anions enlarged to include five additional members (species II, III, IV, V and VI in Scheme 1).^3–7^ Synthesis, structures and reactivity for this series of six aluminyl anions and their associated metal complexes were reviewed and reactions including nucleophilic substitution, oxidative addition, cycloaddition, reductions and oxidations were summarized, with special focus on the C–H and C–F activation, small molecules activation and the ring opening of aromatics.^8^

**Fig. 1:**
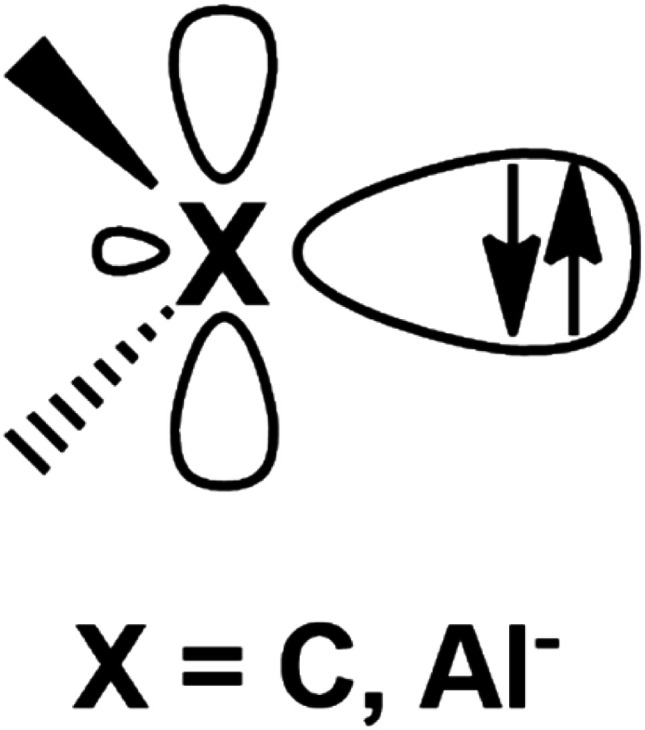
Schematic representation of the lone pair and the vacant 2p/3p orbital on the C and Al atoms of carbenes and aluminyls, respectively.

Metal–aluminyl complexes are of particular interest, since the electronic properties of aluminyls closely resemble those of carbenes, which represent a class of ubiquitous and extremely versatile ligands in coordination chemistry.^9–13^ Indeed, as shown in Fig. 1, in the most relevant frontier orbitals (FMO) manifold, both aluminyls and singlet carbenes feature (i) a lone pair on the Al/C atom respectively, associated with their donating ability, which is responsible for their nucleophilic behavior and (ii) an empty p orbital (3p for Al and 2p for C), associated with their acceptor capability, which is, in turn, responsible for their electrophilicity.^14,15^

The electronic structure of carbenes is intimately related to their nucleophilic/eletrophilic behavior, with the stability of the HOMO, which allocates the lone pair, being an indicator of their nucleophilicity (the higher the HOMO energy, the more nucleophilic the carbene) and the stability of the LUMO, which can be ascribed to the empty 2p orbital of C, being a measure of their electrophilicity (the lower the LUMO energy, the more electrophilic the carbene).^15^ Another particularly interesting feature of carbenes is that their electronic properties can be sizably modulated by applying selected modifications on their structure.^14–16^ For instance, the nucleophilicity of carbenes can be enhanced by modifying the size of the ring, where, in the so-called Ring Expanded N-Heterocyclic carbenes (RENHCs),^17,18^ the wider N–C–N angle of six- and seven-membered NHCs corresponds to an increase of the p-character of the lone pair, which, in turn, destabilizes the HOMO and enhances its nucleophilic behavior.^16,19^ Analogously, as it has been reported for anti-Bredt carbenes,^20^ a pyramidalized N substituent, due to a reduced N-to-C π-donation, is able to decrease the LUMO energy enhancing their electrophilicity. Additionally, the synthesis of cyclic (alkyl)(amino)carbenes^21–23^ (CAACs) sanctioned the advent of a class of cyclic carbenes with enhanced electrophilicity (due to lowered N-to-C π-donation which induces a LUMO stabilization) and nucleophilicity (decreased electronegativity of one of the α-subtituent which induces HOMO destabilization).^19,21^

At a first sight, the analysis of the most relevant molecular orbitals (MOs) of the aluminyl anions I–III and V–VI in their monomeric form reported in ref. 8 revealed a qualitatively similar relationship between electronic effects and geometrical structure. Indeed, HOMO destabilization was found for the seven-membered III with respect to the six-membered II (as a consequence of the ring size) anion and for V and VI anions (due to the reduced electronegativity of the carbon α atoms). Similarly, the MO corresponding to the 3p empty orbital of Al (which, however, unlike carbenes, does not correspond to LUMO in all cases) is stabilized not only for CAAC-like aluminyls V and VI (due to reduced/absent π-donation towards Al) but also for I, for which, in addition to a small effect of the Al–O σ* character (thus implying the existence of a bond between Al and the O atom of NON), the reduced N-to-Al π-donation is induced by geometrical restraints similarly to what happens for anti-Bredt carbenes. In addition to this, analogies between Arduengo-type carbenes and isoelectronic analogues with the group 13 elements (B, Al, Ga, In) have been also computationally previously predicted.^24^

Notably, it has been extensively reported that the relationship between electronic properties and structure can be transferred to the features of the coordination bond in metal–NHC complexes. Particularly, in the case of gold(i)–NHC complexes, the Au–C bond can be described, according to the Dewar–Chatt–Duncanson (DCD) model, as consisting of a σ C-to-Au donation and a non-negligible Au → C π back-donation^10,25–30^ and both components are found to be highly tunable with respect to the structural modifications discussed earlier.^30^ Similar tuning has also been reported for the trans effect in gold(i)–alkynyl complexes.^31^

Analogous experimental and/or theoretical systematic studies assessing this relationship for metal–aluminyl complexes have been never reported to the best of our knowledge, despite the interesting and novel chemistry of metal–aluminyl compounds. In this regard, it was recently revealed that the gold complex of aluminyl anion I, the [^*t*^Bu_3_PAuAl(NON)] complex, is surprisingly able to insert CO_2_ into the Au–Al bond, leading to the [^*t*^Bu_3_PAuCO_2_Al(NON)] product with the CO_2_ carbon atom coordinated to Au.^32^ Based on mechanistic and electronic structure analysis, we recently unraveled a bimetallic radical-like reactivity towards CO_2_, with the Au–Al bond acting as a nucleophilic site, consistently with an electron-sharing, weakly polarized Au–Al bond, which is also assisted by the electrophilic behavior of Al through its empty 3p_*z*_ orbital.^33^

Within this framework, the Au–Al bond/reactivity relationship is certainly worth exploring to advance our knowledge on the nature of this new type of Au–Al bond and on its possible tuning through structural modifications of the aluminyl scaffold.

The aluminyl chemistry is currently widely studied and the number of aluminium nucleophiles is rapidly expanding.^34^ However, the experimentally synthesized and characterized aluminyl nucleophiles depicted in Scheme 1 sufficiently differ each other from a structural point of view to our aim. Anion I is a three-coordinated Al species, anions II and III are two-coordinated Al species supported by bidentate six- and seven-membered diamino ligands, K_2_[Al(^Si^NON)]_2_ (^Si^NON = {O(SiMe_2_NDipp)_2_})^3^ and K_2_[Al(NCCN)]_2_ (NCCN = {CH_2_SiMe_2_N(Dipp)}_2_, Dipp = 2,6-iPr_2_C_6_H_3_),^4^ respectively. Anion IV is a two-coordinated Al by the chelating β-diketiminate six-membered planar ligand, where a backbone Me group is deprotonated K[Al(^Dipp^BDI-H)] (^Dipp^BDI-H = H_2_C=C(N-Dipp)-C(H)=C(Me)-N-Dipp).^5^ Substitution of nitrogen with carbon atoms leads to alkyl aluminyl anions (species V and VI, Scheme 1). Both compounds [K(toluene)_2_-Al{(C(SiMe_3_)_2_CH_2_)_2_}]^6^ and [K(12-crown-4)_2_][Al(CAA)]^7^ (CAA = cyclic alkyl amino = {C_2_H_2_C(SiMe_3_)_2_NAd}, Ad = 1-adamantyl) feature 5-membered aluminium heterocycles, essentially differing by two carbon atoms (anion VI) *vs.* one carbon and one nitrogen atoms (anion V) as ligand donors in the ring.

In this work we investigate the features of the Au–Al bond in this series of gold–aluminyl complexes in close relationship with the Au–C bond in gold-carbene analogues. Although carbene homologues of I–VI have not been synthesized yet, at least to our knowledge, gold complexes bearing saturated six- and seven-membered RENHCs ligands have been reported previously.^35,36^ We also analyze the mechanism of the CO_2_ insertion in the [^*t*^Bu_3_PAuX] (X = aluminyl anions I, II, III, IV, V and VI, Scheme 1) complexes in tight connection with their electronic structure, within the interpretative framework provided in ref. 33. To directly compare the aluminyl anions I–VI bonding properties towards Au and the reactivity of the corresponding complexes with carbon dioxide, a common [^*t*^Bu_3_PAu]^+^ metal fragment has been selected (Scheme 1).

We unravel that actually aluminyls show a different behavior with respect to carbenes as gold ligands: a highly-covalent, weakly polar Au–Al bond is formed in all gold-aluminyl complexes, revealing a different nature with respect to the Au–C bond in gold-carbene analogues, which is mainly dative. As a consequence, the possibility of tuning the features of the Au–Al bond by imposing structural modifications on the aluminyl is highly reduced. As a result of the scarce tunability of their electronic structures, the six complexes under study are demonstrated to show no remarkable differences when reacting with CO_2_, favoring in all cases the formation of similarly stable [^*t*^Bu_3_PAuCO_2_X] insertion products.

## Results and discussion

### Comparative analysis of the Au–Al bond

By relying on the use of Energy Decomposition Analysis (EDA),^37,38^ and Charge Displacement (CD) analysis,^39–41^ combined with the ETS-NOCV (Extended Transition State – Natural Orbitals for Chemical Valence)^42^ approach, we investigate in detail the features of the bond between the gold and aluminyl fragments within a quantitative and comparative framework, to unravel if and how such features can be modulated by the structural modifications imposed by the aluminyl ligand scaffold.

Firstly, based on the computational protocol reported in ref. 43 and 44, we carry out a comparative EDA using differently charged fragments (*i.e.* [^*t*^Bu_3_PAu]^+/0/−^–[X]^−/0/+^) in order to assess which fragmentation scheme is the most suitable for the description of the Au–Al bond. This approach, coherently with the previously reported results for [^*t*^Bu_3_PAu-I] and [^*t*^Bu_3_PAu-II],^33,45^ reveals that in all the cases the best fragmentation scheme is in terms of neutral doublet [^*t*^Bu_3_PAu]˙ and [X]˙ fragments (see Tables S1–S4† in the ESI for the results).

**Fig. 2:**
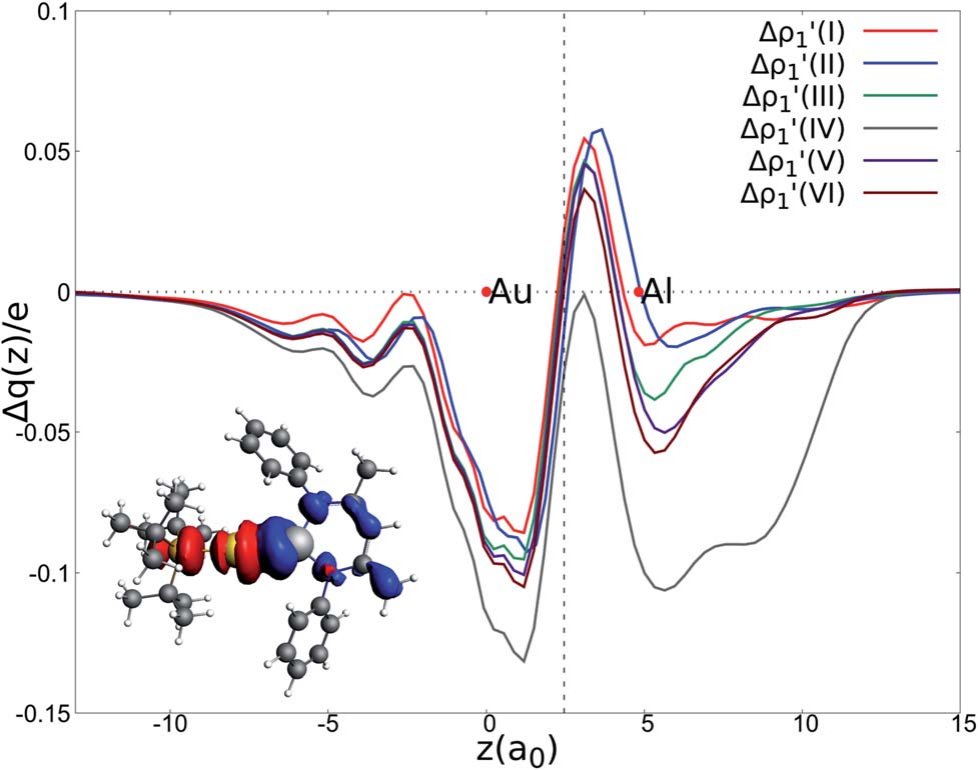
Charge Displacement (CD-NOCV) curves associated with the 
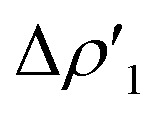
 NOCV deformation density for the interaction between doublet [^*t*^Bu_3_PAu]˙ and [X]˙ (X = I–VI) fragments for complexes [^*t*^Bu_3_PAuX]. Red dots indicate the average position of the nuclei along the *z* axis. The black dashed line indicates the average position of the isodensity boundary. Positive (negative) values of the curve indicate right-to-left (left-to-right) charge transfer. *Inset:* isodensity surfaces associated with the 
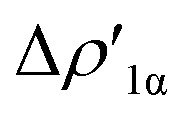
 NOCV deformation density for complex [^*t*^Bu_3_PAuIV]. Charge flux is red-to-blue. Isodensity value is 1 me a_0_^−3^. Results for [^*t*^Bu_3_PAuI] and [^*t*^Bu_3_PAuII] are taken and adapted from ref. 33 and 45, respectively.

Next, we combine CD and ETS-NOCV approaches for analyzing the Au–Al bond in the complexes under study. The numerical results of the CD-NOCV analysis are reported in Table 1, with the CD-NOCV curves associated with the 
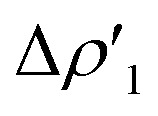
 NOCV deformation density depicted in Fig. 2. All the curves associated with the different NOCV deformation densities 
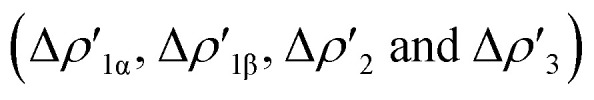
 and the corresponding isosurfaces are reported in the ESI (Fig. S1–S5†).

**Table 1:** Orbital interaction energies (Δ*E*_oi_^*k*^) and charge transfer (CT^*k*^) associated with the first three NOCV deformation densities for the interaction between neutral doublet [^*t*^Bu_3_PAu]· and [X]˙ (X = I–VI) for complexes [^*t*^Bu_3_PAuX]. The overall Δ*E*_oi_ and Δ*E* values from the EDA are also reported. Values for [^*t*^Bu_3_PAuI] and [^*t*^Bu_3_PAuII] are taken from ref. 33 and 45, respectively

X	I	II	III	IV	V	VI
CT^1α^	−0.272	−0.312	−0.284	−0.325	−0.287	−0.298
Δ*E*_oi_^1α^	−32.7	−33.1	−34.3	−43.6	−33.9	−34.6
CT^1β^	0.299	0.299	0.304	0.300	0.304	0.307
Δ*E*_oi_^1β^	−24.5	−23.4	−24.0	−23.6	−23.7	−23.8
CT^1^	0.027	−0.013	0.020	−0.025	0.017	0.009
Δ*E*_oi_^2^	−4.3	−4.3	−4.4	−4.2	−4.2	−4.7
CT^2^	−0.030	−0.038	−0.029	−0.020	−0.024	−0.032
Δ*E*_oi_^3^	−3.3	−3.6	−3.5	−3.4	−3.8	−3.5
CT^3^	−0.018	−0.027	−0.014	−0.015	−0.020	−0.020
Δ*E*_oi_	−71.5	−70.9	−73.4	−84.6	−72.1	−72.8
Δ*E*	−87.6	−87.1	−84.7	−90.7	−83.5	−83.3

The results of the CD-NOCV analysis unequivocally point out a clear outcome concerning the nature of the Au–Al bond: structural modifications on the aluminyl scaffold appear to be not relevant for tuning the features of the Au–Al bond.

In detail, in complexes [^*t*^Bu_3_PAuIII]-[^*t*^Bu_3_PAuVI], the Au–Al bond can be envisaged to be mainly consisting of two opposite charge transfers (CTs), namely an Al-to-Au CT 
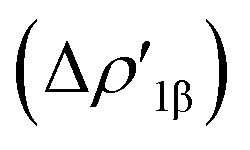
 and an Au-to-Al charge flux 
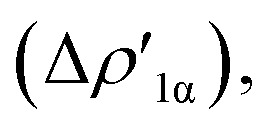
 as found for [^*t*^Bu_3_PAuI] and [^*t*^Bu_3_PAuII] in ref. 33 and 45, respectively. On a quantitative ground, the former is surprisingly very similar for all the complexes. Indeed, the extent of the Al-to-Au charge flux is practically identical for all the complexes (the corresponding CT^1β^ values vary in the 0.299–0.307*e* range, Table 1) and, consistently, the associated stabilizing orbital interaction Δ*E*_oi_^1β^ varies in a very narrow range (1.1 kcal mol^−1^, Table 1). This result is surprising since it suggests that the donor ability of the aluminyl moiety, despite the structural differences between anions I–VI, remains substantially unaltered.

Similarly, the Au-to-Al charge flux appears also to be only marginally affected by such modifications. Although the Au-to-Al charge transfer varies in a slightly wider range for complexes [^*t*^Bu_3_PAuI]-[^*t*^Bu_3_PAuIII] and [^*t*^Bu_3_PAuV]-[^*t*^Bu_3_PAuVI] (CT^1α^ −0.272/−0.312*e*), the associated Δ*E*_oi_^1α^ values clearly demonstrate that, overall, no significant influence of the aluminyl ligand is detected along the series (Δ*E*_oi_^1α^ varies in a range of 1.9 kcal mol^−1^ for these complexes). Analogously, the overall interaction energies Δ*E* in this series vary in a particularly narrow range (−87.6/−83.3 kcal mol^−1^, see ref. 33, 45 and Tables S1–S4† in the ESI). This is again an outstanding result, particularly because the acceptor ability (electrophilicity) of the group 13 aluminyl anion has been recently demonstrated to be a crucial factor in these complexes for determining the degree of covalency of the Au–X bond and potentially their reactivity towards CO_2_.^33^ Despite this homogeneity, some differences can be observed for complex [^*t*^Bu_3_PAuIV]. As it can be envisioned from the data in Table 1 and from the CD-NOCV curve in Fig. 2, the [Al(^Dipp^BDI-H′)]˙ fragment seems to have an enhanced ability of accepting charge from gold, with both increased CT^1α^ and Δ*E*_oi_^1α^ values (−0.325*e* and −43.6 kcal mol^−1^, respectively). Upon inspection of the isodensity surface related to the Δ*E*_oi_^1α^ NOCV deformation density (see inset of Fig. 2), it is clear that this different behavior in [^*t*^Bu_3_PAuIV] is determined by the planarity of the [Al(^Dipp^BDI-H′)] anion, which, as clearly shown in the isodensity picture, is responsible for an hyperconjugation interaction, where the Au–Al σ bond formally interacts with the π-system of the planar [(^Dipp^BDI-H′)]^2−^ backbone.

Interestingly, we should note that complex [^*t*^Bu_3_PAuI], in which the [Al(NON′)] is the only aluminyl bearing a tri-coordinated Al in the series, is very much analogous to the di-coordinated Al aluminyls [^*t*^Bu_3_PAuII] and [^*t*^Bu_3_PAuIII], thus suggesting that the oxygen atom of NON has not a direct effect on the Au–Al bond. Indeed, the isodensity surfaces of the 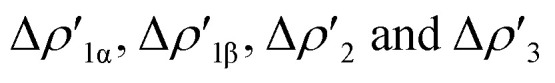 NOCV deformation densities (Fig. S1–S4†) do not show any depletion/accumulation pattern involving the NON oxygen atom, thus ruling out a significant electronic influence due to the existence of a possible Al–O bond.

Overall, these analogies and differences are well explained by the CD-NOCV curves in Fig. 2 and the associated CT values of the total 
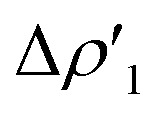
 component. As it can be seen, most of the net curves practically overlap (and have values close to zero) in the region of the Au–Al bond, clearly indicating an overall electron-rich, highly covalent and weakly polarized Au–Al bond, as also demonstrated by the very small values of net CT (Table 1). Yet again, complex [^*t*^Bu_3_PAuIV] is an outlier in this series, as it can be envisaged by the corresponding CD-NOCV curve, which suggests a slightly more significant Au(*δ*^+^)–Al(*δ*^−^) polarization of the bond, consistently with the results discussed earlier.

Analogously to [^*t*^Bu_3_PAuI] and [^*t*^Bu_3_PAuII], complexes [^*t*^Bu_3_PAuIII]–[^*t*^Bu_3_PAuVI] have two dative π back-donation components, which, as demonstrated by the CT values reported in Table 1 and by the CD-NOCV curves shown in Fig. S5,† are very small in magnitude and do not display significant modulation upon variation of the aluminyl moiety.

### Aluminyls/carbenes Au–Al/Au–C bond comparison

Based on the results illustrated in the previous section, it appears that the main features of the bond between gold and aluminyl fragments are hardly affected by the different structural modifications at the aluminyl site, thus highlighting substantial differences with respect to their carbene counterparts. In light of this finding, here we quantitatively assess the carbene/aluminyl ligand relationship.

Firstly, to discuss in more detail the results reported in ref. 8 concerning the similarities between carbenes and aluminyls in terms of frontier molecular orbitals (FMOs), we optimize isolated anions I–VI and their carbene analogues [Y] (Y=C(NON′), C(^Si^NON′), C(NCCN′), C(^Dipp^BDI-H′), C(CAA′), C({C(SiH_3_)_2_CH_2_}_2_), labeled as I^C^–VI^C^ in the order). This allows to quantitatively compare the trends observed for the most relevant FMOs. Their FMO energies are reported in Fig. S6 in the ESI.† As it can be clearly envisaged by the FMO energies along the two series of carbenes and aluminyls, respectively, while qualitatively the two trends are generally alike (corroborating the similarities discussed in ref. 8) from a quantitative perspective we readily realize that the electronic properties of the aluminyls appear to be less responsive to the structural differences. Indeed, for the lone pair orbital (HOMO), in the case of carbenes its energy varies in a much wider range with respect to aluminyls (1.1 *vs.* 0.4 eV, respectively) and a similar pattern is observed for the MO corresponding to the vacant 2p/3p orbital of C/Al (1.2 eV *vs.* 0.6 eV, respectively).

Despite the FMO-based analysis suggests some differences between isolated aluminyls and carbenes, our aim is to assess properly and quantitatively an aluminyls/carbenes comparison as coordination ligands. We decided to study, by relying on the same EDA-CD-NOCV-based protocol used in the previous section, the features of the Au–C bond in the six cationic gold-carbene complexes [^*t*^Bu_3_PAuY]^+^, which represent the analogues of the [^*t*^Bu_3_PAuX] complexes object of this study. The optimized structures of the [^*t*^Bu_3_PAuY]^+^ complexes are depicted in Fig. S7 in the ESI,† and they are qualitatively very similar to those of their aluminyl counterparts.

Firstly, we identify which fragments describe the Au–C bond best, *i.e.* closed-shell [^*t*^Bu_3_PAu]^+^–[Y] or doublet open-shell [^*t*^Bu_3_PAu]˙−[Y]˙^+^ fragments. This analysis is performed by using the EDA approach on both fragmentation schemes, and the results are reported in Table S5 in the ESI.† In contrast to their aluminyl counterparts, the interaction between gold and carbene fragments, as expected, is best described by closed-shell [^*t*^Bu_3_PAu]^+^–[Y] fragments in all cases, as demonstrated by the less stabilizing orbital interaction (which is the indicator for the most suitable fragmentation scheme)^43,44^ for the closed-shell fragments.

This different fragmentation scheme suggests that the Au–C bond has probably a prevailing dative character and this can be quantitatively inferred by the results of the CD-NOCV analysis of the Au–C bond. The most relevant results are reported in Tables 2 and 3. The complete results can be found in Table S6, Fig. S8 and S9 in the ESI.†

**Table 2:** Orbital interaction energies (Δ*E*_oi_^*k*^) and charge transfer (CT^*k*^) associated with the first two NOCV deformation densities 
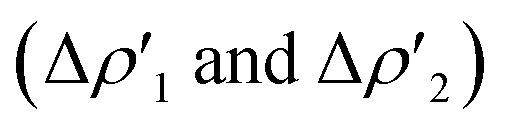
 for the interaction between closed-shell [^*t*^Bu_3_PAu]^+^ and [Y] fragments Y I^C^–VI^C^) for complexes [^*t*^Bu_3_PAuI^C^]^+^–[^*t*^Bu_3_PAuVI^C^]^+^. Energies are reported in kcal mol^−1^, CT values are reported in electrons

X	I^C^	II^C^	III^C^	IV^C^	V^C^	VI^C^
Δ*E*_oi_^1^	−46.6	−49.8	−50.6	−46.6	−47.6	−49.6
CT^1^	0.364	0.381	0.390	0.358	0.360	0.382
Δ*E*_oi_^2^	−9.7	−8.3	−8.6	−9.0	−9.9	−11.9
CT^2^	−0.057	−0.057	−0.062	−0.056	−0.048	−0.097

**Table 3:** Percentage variation (*ε*_1_ = [(Δ*E*_oi_^1β^ − Δ*E*_oi_^1β^_ref_)/Δ*E*_oi_^1β^_ref_] × 100; *ε*_2_ = [(Δ*E*_oi_^2^ − Δ*E*_oi_^2^_ref_)/Δ*E*_oi_^2^_ref_] × 100) with respect to a reference value (values for I and I^C^) of the orbital interaction associated to Δ*E*_oi_^1β^ and Δ*E*_oi_^2^ for complexes [^*t*^Bu_3_PAuI]–[^*t*^Bu_3_PAuVI] and Δ*E*_oi_^1^ and Δ*E*_oi_^2^ for complexes [^*t*^Bu_3_PAuI^C^]^+^–[^*t*^Bu_3_PAuVI^C^]^+^

	*ε*_1_		*ε*_2_	
	Aluminyl	Carbene	Aluminyl	Carbene
I/I^C^	0.0	0.0	0.0	0.0
II/II^C^	0.0	6.9	0.0	−14.4
III/III^C^	1.7	8.6	2.3	−11.3
IV/IV^C^	0.3	0.0	2.3	−7.2
V/V^C^	1.7	2.1	2.3	2.1
VI/VI^C^	2.7	6.4	9.3	22.7

The CD-NOCV results unequivocally depict the Au–C bond in complexes [^*t*^Bu_3_PAuI^C^]^+^–[^*t*^Bu_3_PAuVI^C^]^+^ as a “classical” metal–ligand coordination bond, with the main bond components that can be described within the framework of the Dewar–Chatt–Duncanson model. Indeed, consistently with the results reported in the literature for the Au(i)–NHC bond,^30,46^ the main and first NOCV component is, in all the cases, a C-to-Au charge transfer of σ symmetry that can be envisaged as a σ donation 
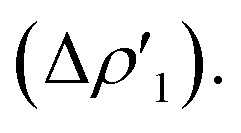
 Additionally, a gold-to-carbon π back-donation component parallel to the Au–N–C–N plane of significant extent can be recognized for all the complexes 
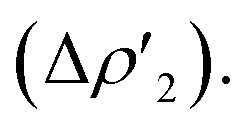
 Finally, two additional minor dative components (namely a σ back-donation and a perpendicular π back-donation) are found for all complexes (see Table S6† for the corresponding CT and Δ*E*_oi_^*k*^ values), coherently with previous literature results.^30,46^

Since Al and C belong to both different groups and periods in the periodic table one may ask if the interplay between dative and electron-sharing nature bonds with gold could be influenced by the change along the group or the period. From the results we recently reported on a comparative analysis within the same framework of gold–aluminyl, -gallyl and -indyl complexes, we unraveled that, although the Au(*δ*^+^)–X(*δ*^−^) polarization increases on descending along group 13, the Au–X bond (X = Al, Ga, In) is in all cases best described as an electron-sharing bond with different degrees of polarization,^45^ thus implying that the bond nature (and the electronic structure of the complexes) is mainly driven by a group effect. This is also confirmed by carrying out a comparative EDA using different fragmentation schemes on the boryl analogue of complex with VI (VI^B^) and the silylene analogue of VI^C^ (VI^Si^) as test cases (Tables S7 and S8 in the ESI†). The analysis reveals a predominant Au–B electron-sharing character for complex with VI^B^ and a dative Au–Si bond for complex with VI^Si^. Therefore, the electronic structure (and the gold–ligand bond nature) differences in the aluminyl *versus* carbene complexes can be inferred to be mainly dictated by a group factor (with group 14 ligand favoring dative and group 13 ligand favoring electron-sharing type bonds with gold), rather than by a period (2nd *versus* 3rd) effect.

From a quantitative perspective, structural modifications of the carbenes and aluminyls scaffolds appear to have different effect on the Au–X bond in terms of tunability. Comparison between the trends of the CD-NOCV results along the series of carbenes (see Table 2) and aluminyls (see Table 1) clearly points out that the Au–C bond is modulated more efficiently with respect to the Au–Al bond.

In detail, the C-to-Au σ donation in carbene complexes appears to have a similar nature with respect to the Al-to-Au CT 
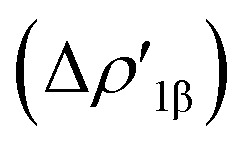
 in aluminyl complexes, since they both reflect the donor power of the aluminyl/carbene ligand (see the very similar related isosurfaces in Fig. S9†). However, these two components (the donor ability) display a different degree of variability along the corresponding series. We can quantitatively infer the degree of variability within each series by relying on the relative percentage variation values *ε*_*1*_ associated with Δ*E*_oi_^1β^ and Δ*E*_oi_^1^ for aluminyls and carbenes, respectively (we resort to the use of this quantity to make the comparison as meaningful as possible). As shown in Table 3, *ε*_*1*_ values vary in a much narrower range for aluminyls with respect to carbenes (0.0/2.7% *vs.* 0.0/8.6%, respectively), thus indicating an enhanced modulability of the Au–C bond. A similar pattern is observed for the π back-donation component, for which relative percentage variation values (*ε*_2_) clearly underline that this bond component is highly tunable upon structural modifications of the carbene ligand (*ε*_2_ varies in the −14.4/22.7% range). Conversely, also in agreement with the energetic trends observed for the FMOs of the isolated aluminyls, the degree of tunability is highly reduced in the case of aluminyls, with *ε*_2_ varying in a much narrower range (0.0/9.3%).

**Fig. 3:**
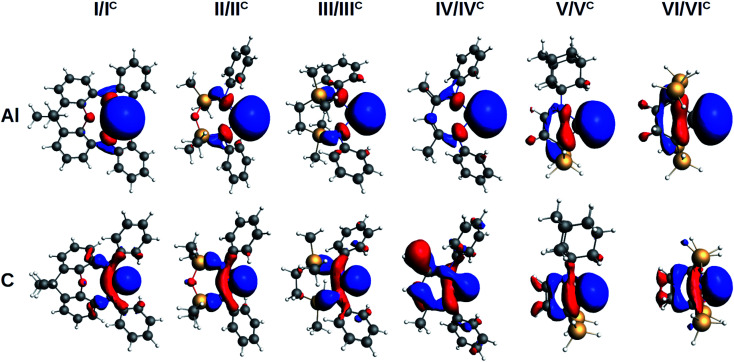
Isosurfaces of the HOMO of aluminyls I–VI (first row) and carbenes I^C^–VI^C^ (second row). Isovalue for all surfaces is 30 me a_0_^−3^.

The evaluation of the strength and basicity of the lone pair of the I–VI and I^C^–VI^C^ ligands also gives an idea of the different nature of these species.^45^ On the basis of the gas phase proton affinity and the corresponding HOMO energy and composition properties, the aluminyls emerge as more reactive and basic ligands than carbenes (proton affinities in the −349.6/−362.1 kcal mol^−1^ range for aluminyls and −267.9/−301.3 kcal mol^−1^ range for carbenes, see Table S9 in the ESI†), as expected. However, whereas for carbenes the proton affinities span a range of 33.4 kcal mol^−1^, for aluminyls they span a much more reduced range (12.5 kcal mol^−1^), thus suggesting once again a reduced tunability of aluminyls upon ligand scaffold change. The peculiarity of the aluminyl ligands lone pair with respect to that of carbenes is strikingly evident by inspection of the isodensity pictures of the corresponding HOMOs, depicted in Fig. 3.

The aluminyls lone pair HOMO is very diffuse, mainly centred at the Al site, and it is poorly delocalized on the ligand, whereas the carbenes lone pair HOMO is much less diffuse and delocalizes over the ligand scaffold to a greater extent (for HOMOs atomic composition and energy see Table S9 in the ESI†), which explains why the structure of the ligands represents a control factor on their electronic properties, while such control is much reduced in the case of aluminyls.

This comparative analysis clarifies that, despite being isoelectronic and isolobal, aluminyls and carbenes display a very different behavior as coordination ligands, with the highly covalent Au–Al bond being poorly tunable upon structural modifications of the aluminyl. In the following section we will discuss the impact of such reduced tunability of the bond in the reaction of complexes [^*t*^Bu_3_PAuI]-[^*t*^Bu_3_PAuVI] with CO_2_.

### Mechanistic study and electronic structure/reactivity relationship

**Fig. 4:**
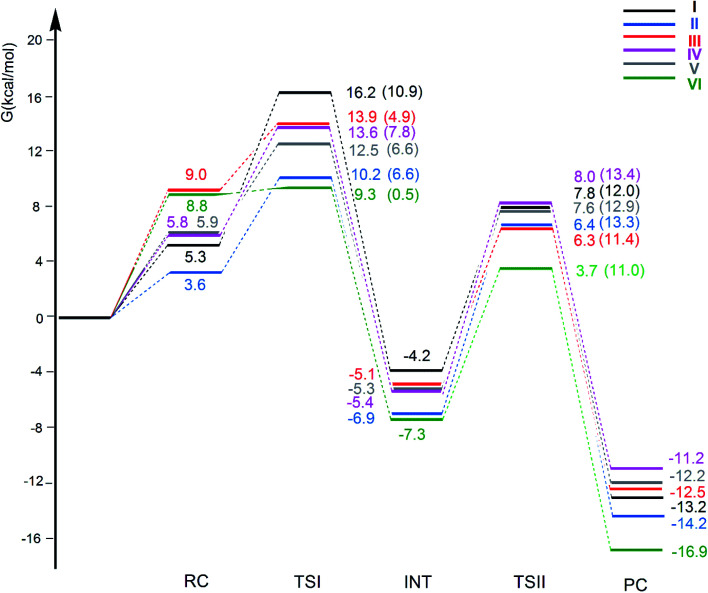
Free energy reaction profile for the CO_2_ insertion into the Au–Al bond in the [^*t*^Bu_3_PAuAl(NON′)] (I, black lines), [^*t*^Bu_3_PAuAl(^Si^NON′)] (II, blue lines), [^*t*^Bu_3_PAuAl(NCCN′)] (III, red lines), [^*t*^Bu_3_PAuAl(^Dipp^BDI-H′)] (IV, violet lines), [^*t*^Bu_3_PAuAl(CAA′)] (V, grey lines) and [^*t*^Bu_3_PAuAl{C(SiH_3_)_2_CH_2_}_2_] (VI, green lines) complexes. Δ*G* values refer to the energy of the separated reactants taken as zero. Activation free energy barriers are reported in parentheses. The results for [^*t*^Bu_3_PAuI] and [^*t*^Bu_3_PAuII] have been taken and adapted from ref. 33 and 45.

The free energy profiles for the CO_2_ insertion into the Au–Al bond in the [^*t*^Bu_3_PAuX] (X = I, II, III, IV, V and VI) complexes have been calculated using the same computational setup as that in ref. 33 and 45 (see Computational details section) for a consistent comparison with the gold-aluminyl [^*t*^Bu_3_PAuI] and [^*t*^Bu_3_PAuII] complex results. They are compared in Fig. 4. Optimized structures of all the compounds and corresponding reactant complexes (RC), transition states (TSI and TSII), intermediates (INT) and product complexes (PC) are reported in the ESI (Fig. S10–S15†).

**Fig. 5:**
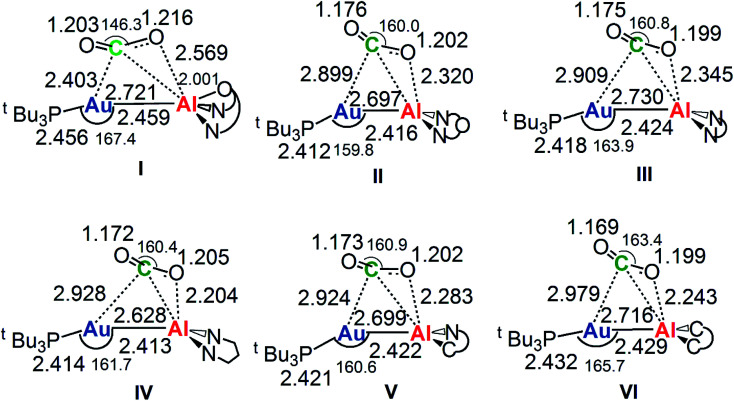
Selected interatomic distances (in Å) and bond angles (degrees) are given with the sketched TSI structures of [^*t*^Bu_3_PAuX] (X = I, II, III, IV, V, VI) complexes. The results for [^*t*^Bu_3_PAuI] and [^*t*^Bu_3_PAuII] have been taken and adapted from ref. 33 and 45.

The reaction profiles in Fig. 4 are qualitatively very similar. In the first step, the nucleophilic attack to the CO_2_ carbon atom by the Au–Al bond has a comparatively low activation free energy barrier, in the range 0.5–10.9 kcal mol^−1^. In absolute value, the six TSIs lie above the corresponding separated reactants in the range 16.2–9.3 kcal mol^−1^. Structures of transition states TSI for all the [^*t*^Bu_3_PAuX] (X = I, II, III, IV, V, VI) complexes are sketched with selected geometrical parameters in Fig. 5.

At the TSI geometry, a very similar bending of CO_2_ (160.0–163.4°) and asymmetry between the two C–O bonds (1.169/1.176 *vs.*1.199/1.205) can be observed for complexes with II, III, IV, V and VI anions, whereas both CO_2_ bending (146.3°), C–O asymmetry and elongation (1.203 *vs.* 1.216) are more pronounced for I. Interestingly, we recently reported that [^*t*^Bu_3_PAuI] features a peculiar topology of the potential energy surface (PES) around TSI which is very flat, leading to locate an almost degenerate TSI structure where the CO_2_ bending is 159.5° and C–O asymmetry is 1.175/1.199 Å, with an oxygen atom of CO_2_ at 2.314 Å distance from Al, which is completely in line with all the other TSI structures.^45^ It is interesting to observe that the free energies of TSIs (and the corresponding Δ*G*^#^ values) do not correlate with the CO_2_ bending, *i.e.* the free energy barrier range (10.9–0.5 kcal mol^−1^) corresponds to the same CO_2_ bending, which is around 160° for all the complexes. This result is in full agreement with the finding in ref. 45 that carbon dioxide bending is not a good indicator of its activation.

Formation of intermediate INT leads to a four-membered (Au–C–O–Al) cyclic structure for all the complexes, with an Au–Al bond slightly larger for [^*t*^Bu_3_PAuI] (2.623 Å *vs.* 2.602–2.572 Å, respectively) and a slightly higher stabilization (20.4 kcal mol^−1^*vs.* 19.0–16.6 kcal mol^−1^, respectively) with respect to complexes with II, III, IV, V and VI aluminyls. The CO_2_ bending and asymmetry between the two C–O bonds is very comparable in all the systems (see Fig. S10–S15†).

**Fig. 6:**
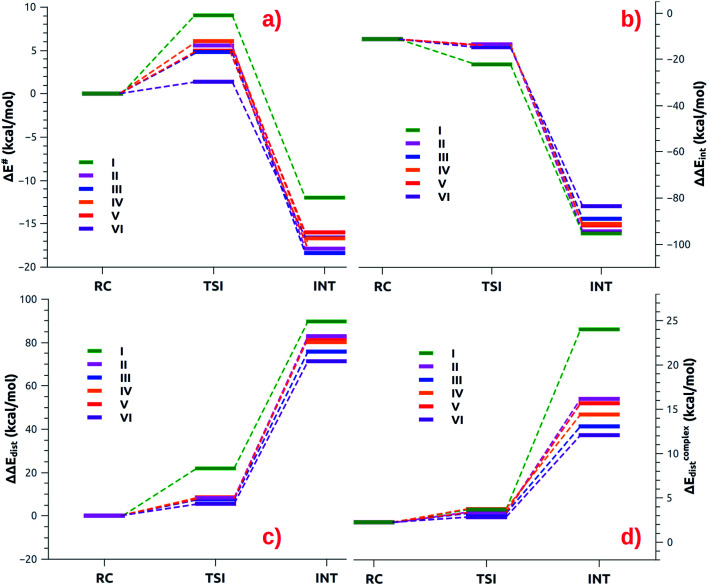
(a) ASM diagrams for the electronic energy variation (Δ*E*) along the reaction path connecting RC, TSI and INT structures for complexes [^*t*^Bu_3_PAuI]-[^*t*^Bu_3_PAuVI]. (b) ASM diagrams for the variation of the interaction energy stabilization (ΔΔ*E*_int_) along the reaction path connecting RC, TSI and INT structures for complexes [^*t*^Bu_3_PAuI]-[^*t*^Bu_3_PAuVI]. (c) ASM diagrams for the overall variation of the distortion energy penalty (ΔΔ*E*_dist_) along the reaction path connecting RC, TSI and INT structures for complexes [^*t*^Bu_3_PAuI]-[^*t*^Bu_3_PAuVI]. (d) ASM diagrams for the penalty due to the distortion of the complex (Δ*E*^complex^_dist_) along the reaction path connecting RC, TSI and INT structures for complexes [^*t*^Bu_3_PAuI]-[^*t*^Bu_3_PAuVI]. Results for [^*t*^Bu_3_PAuI] and [^*t*^Bu_3_PAuII] have been taken from ref. 33 and 45, respectively.

The small differences observed in the first step of the reaction path can be well rationalized by relying on the Activation Strain Model (ASM),^47–49^ which decomposes the energy along the reaction path in two contributions: one arising from the interaction between the fragments (ΔΔ*E*_int_) and the other representing the distortion of the interacting fragment from their relaxed geometry to their in-adduct configuration (ΔΔ*E*_dist_, see the Methodology section in the ESI† for a brief description of the approach). The numerical results of the ASM approach for complexes [^*t*^Bu_3_PAuI]-[^*t*^Bu_3_PAuVI] are reported in Tables S10–S11 in the ESI,† whereas the related Activation Strain diagrams (ASD) are depicted in Fig. 6.

As shown in Fig. 6a, most complexes feature similar electronic activation barriers involving TSI and similar stabilization extent of the intermediate INT. In particular, for complexes [^*t*^Bu_3_PAuII]-[^*t*^Bu_3_PAuV] narrow ranges for the electronic activation barriers are observed (4.8–6.1 kcal mol^−1^ range, see Table S10†) and for the corresponding INT stabilization (−16.0/−18.4 kcal mol^−1^ range, see Table S11†), consistently with the very similar nature of the Au–Al bond described in the previous sections. This is further confirmed by investigating the nature of the interaction between CO_2_ and complexes [^*t*^Bu_3_PAuI]–[^*t*^Bu_3_PAuVI] at both TSI and INT by relying on EDA-NOCV and CD approaches (the results are reported in Tables S12, S13, Fig. S16 and S17 in the ESI†). Unsurprisingly, the analysis reveals qualitative and quantitative analogies concerning the interactions taking place at TSI and INT in all cases. Indeed, consistently with the previously reported results for [^*t*^Bu_3_PAuI] and [^*t*^Bu_3_PAuII],^33,45^ in the first step of the reaction, each complex interacts with CO_2_ mainly through a charge transfer from the electron-rich Au–Al bond (which behaves as a nucleophilic site) towards the LUMO of CO_2_. The nature of this interaction can be clearly inferred by the isodensity pictures of the related NOCV deformation density 
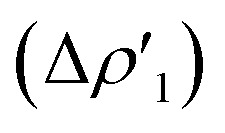
 in Fig. S16 and S17 in the ESI.† In addition, in all the cases, an inverse charge flux assists the reaction, namely the charge transfer from the HOMO of CO_2_ towards the empty valence 3p_*z*_ orbital of Al (see isosurfaces 
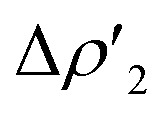
 in Fig. S16 and S17 in the ESI†).

Despite these analogies, the ASM analysis reveals two outliers in the series, *i.e.* complexes [^*t*^Bu_3_PAuI] and [^*t*^Bu_3_PAuVI]. For complex [^*t*^Bu_3_PAuI], as shown in Fig. 6a and consistently with the free energy profiles reported in Fig. 4, the first step features the highest activation barrier (10.9 and 9.0 kcal mol^−1^ in terms of Δ*G*^#^ and Δ*E*^#^, respectively) and the least stable intermediate (−4.2 and −12.0 kcal mol^−1^ in terms of Δ*G* and Δ*E*, respectively). By decomposing the reaction path we unravel that these differences mainly arise from an increased distortion penalty for [^*t*^Bu_3_PAuI]: while overall ΔΔ*E*_dist_ values for complexes [^*t*^Bu_3_PAuII]-[^*t*^Bu_3_PAuVI] at INT lie in a tight range (71.3–83.0 kcal mol^−1^, Table S8†), [^*t*^Bu_3_PAuI] reveals a higher distortion penalty along the path (89.9 kcal mol^−1^), which, as it can be inferred from the results displayed in Fig. 6d, arises from a higher distortion of the complex when constrained at the INT geometry (Δ*E*^complex^_dist_ values lie in the 11.4/16.2 kcal mol^−1^ range for complexes [^*t*^Bu_3_PAuII]-[^*t*^Bu_3_PAuVI], while the penalty associated with [^*t*^Bu_3_PAuI] is 25.3 kcal mol^−1^, Table S11†), consistently with [^*t*^Bu_3_PAuI] displaying the longest (and thus most distorted from its equilibrium value) Au–Al bond (2.623 Å) at INT. Despite its flexibility, the highly sterically hindered (NON′) ligand gets highly distorted at the strained geometry of INT, resulting in an increased distortion penalty.

On the other hand, complex [^*t*^Bu_3_PAuVI] represents an outlier on the opposite side. It features the lowest activation barrier (0.5 and 1.4 kcal mol^−1^ in terms of Δ*G*^#^ and Δ*E*^#^, respectively) and a very stable intermediate (−7.3 and −16.6 kcal mol^−1^ in terms of Δ*G* and Δ*E*, respectively). In this case, as shown in Fig. 6b and c and from the data in Tables S10 and S11,† the increased stability arises from an enhanced ability of counterbalancing the distortion penalty with favorable and stabilizing interactions. By relying on the EDA results (Tables S12 and S13 in the ESI†), we can see that this enhanced stabilization mainly arises from a reduced Pauli repulsion at both TSI and INT, where such effect becomes very clear. Complexes [^*t*^Bu_3_PAuI]-[^*t*^Bu_3_PAuV] display Δ*E*_Pauli_ values at INT in the 406.2–384.2 kcal mol^−1^ range, while complex [^*t*^Bu_3_PAuVI] has a much lower associated Δ*E*_Pauli_ (356.8 kcal mol^−1^). It is worth reminding that the decrease of Pauli repulsion has been found to be a stabilizing driving force in several catalytic processes involving organic substrates.^50^ In this case, this stabilization, arising from a reduced two-orbital-four-electron repulsion, is mainly due to the reduced steric hindrance and smaller dimension of the aluminyl VI, which should help diminishing the filled orbitals' repulsion (see Tables S14–S18 and Fig. S18–S21† in the ESI for the effect of modelling VI with simplified substituents). It is clear that we are discussing very small differences in a reactivity that, overall, is very similar for all complexes. We also should note that the first step of the reaction is not the rate-determining step (RDS). However, this analysis suggests that the steric hindrance, rather than the electronic properties, of the aluminyl may represent a control factor on the reactivity of these species towards CO_2_.

In the second step of the reaction, the oxygen atom of CO_2_ attack to the electrophilic Al center requires higher activation free energy barriers than those for the nucleophilic attack to the CO_2_ carbon atom, that, however, vary in a narrow range (Δ*G*^#^ = 11.0/13.4 kcal mol^−1^), and a stable product complex PC is formed for all the complexes. The TSII structures are all very similar (see Fig. S10–S15†) and the associated imaginary frequency is very small for all the complexes, indicating a very flat potential energy surface involving the bending of the two [^*t*^Bu_3_PAu] and [X] (X = I, II, III, IV, V, VI) fragments between which the CO_2_ insertion occurs. Overall, the insertion products have very similar relative energies (in the range between −11.2 and −16.9 kcal mol^−1^) and their stability can be related, as discussed in the previous works on [^*t*^Bu_3_PAuI] and [^*t*^Bu_3_PAuII],^33,45^ to a radical-like reactivity of the gold and aluminyl fragments that is able to stabilize the insertion product. Indeed, by analyzing the formation of the products starting from radical fragments (see Scheme S1 and Table S19 in the ESI†), we can clearly see that complexes [^*t*^Bu_3_PAuI]-[^*t*^Bu_3_PAuVI] have similarly stabilizing formation energies (Δ*E* in the range −106.0/−111.4 kcal mol^−1^) from radical fragments, in accordance with the picture of a radical-like reactivity.

We also computed the extrusion of CO from the INT or PC complexes and it has revealed to be endergonic for all the anions: the resulting oxide complex [^*t*^Bu_3_PAuOX] [CO] (X = I, II, III, IV, V, VI) has been calculated to be thermodynamically unstable with Δ*G* = 16.6, 14.9, 17.2, 16.9, 15.1 and 11.9 kcal mol^−1^, respectively. This mechanistic perspective supports the picture described in the first section for this series of complexes, *i.e.* that no particularly remarkable reactivity difference between the considered gold-aluminyl complexes can be observed, coherently with the poor tunability of their electronic structure and bonding features.

The Au–Al bond nature/reactivity relationship observed here is fully consistent with the results previously found by us in ref. 45 for the CO_2_ insertion reaction in gold-aluminyl (complex [^*t*^Bu_3_PAuII]), -gallyl and -indyl complexes, where the highly covalent, low polarity Au–Al bond favors the reaction, whereas the increased polarity of the covalent Au(*δ*^+^)–Ga(*δ*^−^) and Au(*δ*^+^)–In(*δ*^−^) bonds makes these complexes weaker nucleophiles in a radical-like mechanism, which appears to be key to stabilize the CO_2_ insertion product. Based on these findings, we expect that the different nature (which is mainly dative) of the Au–C bonds in carbene complexes with I^C^, II^C^, III^C^, IV^C^, V^C^ and VI^C^ ligands would impart a different reactivity with carbon dioxide. Although it is known that free NHCs can capture CO_2_ to form the corresponding imidazolium carboxylates (featuring a C–CO_2_ bond), examples of CO_2_ insertion chemistry have been only reported for f-block M-NHC (M = Y, Ce, U) complexes, where the M-NHC bond is very weak and CO_2_ can coordinate to NHC simply by displacing it from the metal.^51–54^ Indeed, in our case, the searching for INT- and PC-type structures using as an example complex [^*t*^Bu_3_PAuII^C^]^+^ failed, invariably leading to the highly unstable {[^*t*^Bu_3_PAuCO][OC(^Si^NON)]}^+^ fragments, where CO_2_ has been reduced to the CO coordinated to the gold fragment and the O atom coordinated to the carbene ligand (Δ*G* = 22.0 kcal mol^−1^ above the separated reactants). Analogously, optimization of a PC structure, with the CO_2_ carbon atom coordinated to the II^C^ C atom and one CO_2_ oxygen atom coordinated to Au, gives a thermodynamically unstable product (Δ*G* = 20.3 kcal mol^−1^ above the separated reactants), suggesting that an insertion of CO_2_ into the Au–C bonds in the carbenes complexes studied here is not thermodynamically feasible.

## Conclusions

Based on the apparent aluminyl/carbene isolobal analogy, the possibility of tuning the features of the metal–aluminyl bond and its reactivity by imposing structural modifications on the aluminyl scaffold, similarly to what has been well established in carbene chemistry, has been explored in this work. Since in the case of gold, an Au–Al complex has been surprisingly shown to insert CO_2_ at ambient conditions with a bimetallic radical-like reactivity, we have performed a comparative analysis of the electronic structure and reactivity of a series of gold-aluminyl complexes bearing experimentally characterized different aluminyl scaffolds to quantitatively assess this tunability.

The results very clearly demonstrate that aluminyls are different from carbenes when used as coordination ligands to gold. Indeed, while the Au–C bond in Au(i)–NHC complexes has been proven to be of a dative nature and electronically highly tunable through NHC structure modification, all the aluminyl complexes under investigation feature an electron-sharing, weakly polarized Au–Al bond which is poorly influenced by the structural variations of the ligand scaffold.

The computed CO_2_ reaction mechanisms for the series of gold-aluminyl complexes clearly reflect the scarce tunability of the Au–Al bond in the series. Indeed, the computed reaction profiles are highly similar from both a qualitative and quantitative perspective. In all the cases, the insertion of CO_2_ into the Au–Al bond features a bimetallic radical-like reactivity, with the Au–Al bond being the nucleophilic site and the vacant 3p_*z*_ orbital of Al assisting as an electrophilic site. In addition, despite some differences that can be explained in terms of steric hindrance of the aluminyl ligand, the two reaction steps (namely, the Au–Al nucleophilic attack to CO_2_ and CO_2_ attack to electrophilic Al) feature activation barriers of comparable amount and similarly stabilized intermediates/products, highlighting that this reactivity is poorly controlled by electronic effects associated with the different geometrical arrangement of the aluminyl scaffold, which is in line with recent experimental evidence.^65^

We mention that, although the nature of the M–Al bonds in X-type PAlP pincer aluminyl complexes and their reactivity have recently been the subject of several works,^66–69^ a study of the effect of systematic structural modifications of the monodentate anionic aluminyl (I) scaffold on the M–Al bond features and complexes reactivity was still lacking in the literature.

This work fits in the currently expanding literature concerning the aluminyl anions, exploiting their peculiar behavior when used as coordination ligands and it also fits in the framework of ligand design of efficient transition-metal-aluminyl complexes for the CO_2_ capture.

## Computational details

Aluminyl anions I–VI have been slightly simplified as follows: I by replacing the two *tert*-butyl groups at the peripheral positions of the dimethylxanthene moiety with hydrogen atoms and the two Dipp substituents on the nitrogen atoms with phenyl groups (denoted as NON′); II, III and IV by replacing the two Dipp substituents on the nitrogen atoms with phenyl groups (denoted as ^Si^NON′, NCCN′ and ^Dipp^BDI-H′, respectively); V and VI by replacing the methyl groups at the Si by hydrogen atoms (denoted as CAA′ and {C(SiH_3_)_2_CH_2_}_2_, respectively). The same modelling of the NON fragment has been used in ref. 33 and it has been shown to give good agreement with available experimental geometrical data for complex [^*t*^Bu_3_PAuI]. The effect of modelling I–VI with simplified substituents on the gold complexes reaction mechanism and electronic structure calculations has been quantitatively evaluated by using gold complex with VI (and the corresponding stationary points) as test case. As shown in Tables S14–S18 and Fig. S18–S21 in the ESI,† effects of the simplifications are found which are mainly associated with the modified (decreased) steric hindrance of the aluminyl ligand. The modelling of the aluminyl ligands negligibly affects the electronic structure results and the chemical insight presented here, while allowing to reduce the computational cost. Geometry optimizations and frequency calculations of free I–VI, I^C^–VI^C^ ligands and corresponding gold complexes and related minima and transition states for each reaction path (minima with zero imaginary frequencies and transition states with one imaginary frequency) have been carried out using the Amsterdam Density Functional (ADF) code^55,56^ in combination with the related Quantum-regions Interconnected by Local Description (QUILD) program.^57^ The PBE^58^ GGA exchange-correlation (XC) functional, the TZ2P basis set with a small frozen core approximation for all atoms, the ZORA Hamiltonian^59–61^ for treating scalar relativistic effects and the Grimme's D3-BJ dispersion correction were used.^62,63^ Solvent effects were modeled employing the Conductor-like Screening Model (COSMO) with the default parameters for toluene as implemented in the ADF code.^64^ The same computational setup has also been used for the EDA, CD-NOCV,^41^ and ASM calculations and for computing the radical reactions between [X], [CO_2_] and [^*t*^Bu_3_PAu] fragments. EDA, ETS-NOCV and CD-NOCV calculations have been carried out in gas phase, since inclusion of implicit solvation has no effect on their results (see Table S20 and Fig. S22 in the ESI†). This protocol has been used successfully in ref. 33 and 45 to study the [^*t*^Bu_3_PAuAl(NON)] and [^*t*^Bu_3_PAuCO_2_Al(NON)] complexes. The PBE performance has been tested by comparison with results obtained using the hybrid PBE0 functional (see Tables S21–S23, Fig. S23 and S24 in the ESI†). For further details and description of the methods used in this work, see the Methodology section in the ESI.†

## Data availability

All the relevant data (including methodology description, mechanistic, CD-NOCV, AMS and EDA data discussed in the manuscript) are provided within the ESI.†

## Author contributions

D. S., L. B. and P. B. conceived the project, D. S., L. B. and P. B. performed the research, D. S., L. B. and P. B. wrote the manuscript.

## Conflicts of interest

The authors declare no competing financial interests.

## Supplementary Material

SC-013-D2SC00630H-s001
