# Supplementary material for: Unraveling differences in aluminyl and carbene coordination chemistry: bonding in gold complexes and reactivity with carbon dioxide
Source: Chem Sci. 2022 Mar 31;13(16):4623–34. doi: 10.1039/d2sc00630h (PMC9020189; doi:10.1039/d2sc00630h)
Supplement: SC-013-D2SC00630H-s001 [file SC-013-D2SC00630H-s001.pdf]

Electronic Supplementary Material (ESI) for Chemical Science.
This journal is © The Royal Society of Chemistry 2022

Electronic Supplementary Material

Unraveling differences in aluminyl and carbene coordination chemistry: bonding in gold complexes and reactivity with carbon dioxide

Diego Sorbelli,*[ab] Leonardo Belpassi,*[b] and Paola Belanzoni*[ab]

[a]*Department of Chemistry, Biology and Biotechnologies, University of Perugia, Via Elce di Sotto, 8 – 06123, Perugia, Italy*

[b]*CNR Institute of Chemical Science and Technologies "Giulio Natta" (CNR-SCITEC), Via Elce di Sotto, 8 – 06123, Perugia, Italy*

Contents

Methodology S2
Tables S1-S4. Comparative EDA of [^{t}Bu$_3$PAuIII]-[^{t}Bu$_3$PAuVI] S6
Figures S1-S4. Isodensity surfaces from CD-NOCV analysis of [^{t}Bu$_3$PAuI]-[^{t}Bu$_3$PAuVI] S8
Figure S5. CD-NOCV curves of [^{t}Bu$_3$PAuI]-[^{t}Bu$_3$PAuVI] S12
Figure S6. FMOs of free carbenes and aluminyls S13
Figure S7. Structures of carbene complexes S13
Table S5. Comparative EDA of [^{t}Bu$_3$PAuI^C]$^+$-[^{t}Bu$_3$PAuVI^C]$^+$ S14
Table S6. Numerical results of the CD-NOCV analysis of [^{t}Bu$_3$PAuI^C]$^+$-[^{t}Bu$_3$PAuVI^C]$^+$ S15
Figure S8. CD-NOCV curves for complexes [^{t}Bu$_3$PAuI^C]$^+$-[^{t}Bu$_3$PAuVI^C]$^+$ S16
Figure S9. NOCV isosurfaces for [^{t}Bu$_3$PAuI^C]$^+$-[^{t}Bu$_3$PAuVI^C]$^+$ S17
Tables S7-S8. Comparative EDA of [^{t}Bu$_3$PAuVI^{Si}]-[^{t}Bu$_3$PAuVI^B] S18
Table S9. HOMOs and proton affinities of I-VI and I^C-VI^C S19
Figures S10-S15. Optimized structures of reaction paths involving complexes [^{t}Bu$_3$PAuI]-[^{t}Bu$_3$PAuVI] S20
Tables S10-S11. ASM results on complexes [^{t}Bu$_3$PAuI]-[^{t}Bu$_3$PAuVI] S26
Tables S12-S13. EDA results on TSI and INT for complexes [^{t}Bu$_3$PAuI]-[^{t}Bu$_3$PAuVI] S27
Figures S16-S17. NOCV isosurfaces for the main interaction at TSI and INT in complexes [^{t}Bu$_3$PAuI]-[^{t}Bu$_3$PAuVI] S28
Figures S18-S21 and Tables S14-S18. Modelling of the ligand S30
Scheme S1. Formation of insertion products via radical fragments S38
Table S19. Energies for the formation of the insertion products from radical fragments for complexes [^{t}Bu$_3$PAuI]-[^{t}Bu$_3$PAuVI] S38
Table S20 and Figure S22. Solvation effect on EDA and ETS-NOCV S39
Tables S21-S23 and Figures S23-S24. PBE vs. PBE0 results S40
References S44
xyz geometries S45

Methodology

- **Natural Orbitals for Chemical Valence and Charge Displacement analysis**

Natural Orbitals for Chemical Valence (NOCV)[1,2] represents a suitable approach for describing the chemical bond. This approach is based on the rearrangement of the electron density occurring when a chemical bond is formed and such rearrangement can be expressed as electron density difference between the formed adduct (AB) and sum of the densities of the two non-interacting fragments (A and B) frozen in their adduct geometry.

This deformation density can be brought into diagonal contributions in terms of NOCVs. In the NOCV scheme, the charge rearrangement taking place upon bond formation is obtained from the occupied orbitals of the two fragments suitably orthogonalized to each other and renormalized (*promolecule*). The resulting electron density rearrangement ($\Delta\rho'$) can be expressed in terms of NOCV pairs which are defined as the eigenfunctions of the so-called "valence operator"[3–5] as follows:

$$\boldsymbol{\Delta\rho'} = \sum_k \boldsymbol{\nu_k}(|\boldsymbol{\phi}_{+\mathrm{k}}|^2 - |\boldsymbol{\phi}_{-\mathrm{k}}|^2) = \sum_k \boldsymbol{\Delta\,\rho'_k} \qquad [S1]$$

where ϕ_{+k} and ϕ_{-k} are the NOCV pairs orbitals and $\nu_{\pm k}$ are the corresponding eigenvalues. Upon formation of the adduct from the promolecule, a fraction ν_k of electrons is transferred from the ϕ_{-k} to the ϕ_{-k} orbital (donor and acceptor orbitals, respectively).

The NOCV scheme can be coupled with the framework of the Charge Displacement (CD)[6] analysis. The CD analysis allows to quantify the amount of electronic charge that is transferred between the two fragments upon the formation of the A-B bond. The Charge Displacement function (Δq) can be defined as the partial progressive integration on a suitable z-axis of the deformation density $\Delta\rho'$:[7]

$$\boldsymbol{\Delta q(z)} = \int_{-\infty}^{z} \boldsymbol{dz'} \int_{-\infty}^{+\infty}\int_{-\infty}^{+\infty} \boldsymbol{\Delta\,\rho'(x,y,z')dxdy} \qquad [S2]$$

The CD function, $\Delta q(z)$, quantifies at each point of the chosen z-axis (which usually corresponds to the bond axis) the exact amount of electron charge that, upon formation of the bond, is transferred from the right to the left across a plane perpendicular to the bond axis through z.

When coupled with the NOCV scheme, the density rearrangement due to the bond formation between two fragments, ($\Delta\rho'$), is partitioned in different NOCV deformation densities ($\Delta\rho'_k$) and therefore one is able to quantify the charge transfer (CT) associated to the components. Note that only few of the

NOCV pairs contributes to the chemical bond. Therefore, when the CD-NOCV analysis is carried out, usually only the first *Δρ* $\Delta\rho_k$' components are investigated in order to understand which significant chemical contribution to the bond they represent.

Usually we choose to evaluate the charge transfer between A and B by taking the CD value at the "isodensity boundary", i.e. the z-point where equally valued isodensity surfaces of the isolated fragments become tangent.[7,8]

When we apply this scheme to both TSI and INT, with [^{t}Bu$_3$PAuX] (X=**I**, **II**, **III**, **IV**, **V**, **VI**) and [CO_2] as fragments, such approach becomes complicated, since the two fragments display multiple interactions with multiple atomic centres and thus it is clearly impossible to define a unique bond axis and it is very hard to rely on the isodensity boundary for the estimation of the charge transfer. In order to avoid any ambiguity in the definition of the z-axis, we recall an approach that may be useful for evaluating the charge transferred between the [^{t}Bu$_3$PAuX] and [CO_2] fragments at TSI and INT.[9]

Within this approach, the electron density rearrangement (*Δρ'*), which typically shows charge accumulation regions (positive values) and charge depletion regions (negative values), defines two different positive functions, $\Delta\rho^+$ and $\Delta\rho^-$, each equal to the magnitude of the appropriate portion, *i.e.*:

$$\boldsymbol{\Delta\rho^{+/-}(r) = max[\pm\Delta\rho(r)', 0]} \qquad [S3]$$

so that

$$\boldsymbol{\Delta\rho(r)' = \Delta\rho^{+}(r) - \Delta\rho^{-}(r)} \qquad [S4]$$

By defining two arbitrary regions that are associated with the interacting fragments, we can evaluate the charge transfer as follows:

$$\boldsymbol{CT = \int_A \Delta\,\rho(r)'dr = -\int_B \Delta\,\rho(r)'dr} \qquad [S5]$$

By combining Eqs. [S4] and [S5], CT can also be expressed as:

$$\boldsymbol{CT = \int_A \Delta\,\rho^{+}(r)dr - \int_A \Delta\,\rho^{-}(r)dr = -\int_B \Delta\,\rho^{+}(r)dr + \int_B \Delta\,\rho^{-}(r)} \qquad [S6]$$

Ultimately, this approach can also be expressed in the CD-NOCV framework. By combining Equations [S1] and [S5], we can use to this approach for calculating the charge transfer associated to each NOCV deformation density as follows:

$$CT_k = \int_A \Delta\,\rho_k(r)'dr = -\int_B \Delta\,\rho_k(r)'dr \quad [S7]$$

Despite the spatial regions associated to the two interacting fragments being defined arbitrarily, this approach is particularly suitable for the analysis of the interaction between the [^{t}Bu$_3$PAuX] and [CO_2] fragments at INT, being the two fragments well-separated in space.

- **Energy Decomposition Analysis and ETS-NOCV approach**

The Energy Decomposition Analysis (EDA)[10,11] has been used in this work to get additional and complementary insights into the interaction between carbon dioxide and the [^{t}Bu$_3$PAuX] complex in the transition state TSI and intermediate INT. With this approach, the interaction energy between the [^{t}Bu$_3$PAuX] and [CO_2] fragments can be decomposed in different contributions as follows:

$$\Delta E_{\text{int}} = \Delta E^{Pauli} + \Delta V_{elst} + \Delta E_{oi} + \Delta E_{disp} \quad [S8]$$

where ΔE^{Pauli} corresponds the Pauli repulsion interaction between occupied orbitals on the two fragments, ΔV_{elst} represents the quasiclassical electrostatic interaction between the unperturbed charge distribution of the fragments at their final positions, ΔE_{disp} takes into account the dispersion contribution and ΔE_{oi} is the orbital interaction, which arises from the orbital relaxation and the orbital mixing between the fragments, and accounts for electron pair bonding, charge transfer, and polarization.

The orbital interaction term ΔE_{oi} can be further decomposed within the ETS-NOCV[12] scheme into NOCV pairwise orbital contributions ($\Delta E_{oi} = \sum_k \Delta\, E_{oi}^k$) which associates an energy contribution (E_{oi}^k) to each NOCV deformation density ($\Delta\rho_k$).

- **Activation Strain Model**

The Activation Strain Model (ASM)[13–15] allows to get insights into the factors controlling the activation barrier of a process. Within this framework, the activation barrier ($\Delta E^{\#}$) can be decomposed as follows:

$$\Delta E^{\#} = \left[\Delta E_{\text{dist}}^{TSI} - \Delta E_{dist}^{RC}\right] + \left[\Delta E_{\text{int}}^{TSI} - \Delta E_{\text{int}}^{RC}\right] = \Delta\Delta E_{dist} + \Delta\Delta E_{\text{int}} \quad [S9]$$

where the "$\Delta E_{dist}{}^{TSI}$" and "$\Delta E_{dist}{}^{RC}$" terms represent the energy penalty due to the distortion of the fragments (i.e. [$^{t}Bu_3PAuX$] and CO_2) constrained in the structures of the transition state (TSI) and the reactant complex (RC) respectively, whereas "$\Delta E_{int}{}^{TSI}$" and "$\Delta E_{int}{}^{RC}$" represent the interaction energies between the fragments (with the geometries constrained at the ones assumed in the TSI and RC, respectively) in the two structures. These terms can be grouped in the "$\Delta\Delta E_{dist}$" and "$\Delta\Delta E_{int}$" terms, that represent the overall distortion and interaction contributions to the activation barrier, respectively.

Additionally, we can rearrange Equation [S9] in order to express the distortion contributions relatively to the two fragments as follows:

$$\boldsymbol{\Delta\Delta E}_{\text{dist}} = \boldsymbol{E}^{\boldsymbol{TS}}_{\boldsymbol{CO2}} - \boldsymbol{E}^{\boldsymbol{RC}}_{\boldsymbol{CO2}} + \boldsymbol{E}^{\boldsymbol{TS}}_{\boldsymbol{AuX}} - \boldsymbol{E}^{\boldsymbol{RC}}_{\boldsymbol{AuX}}$$
$$\boldsymbol{\Delta\Delta E}_{\text{dist}} = \boldsymbol{\Delta E}^{\boldsymbol{CO2}}_{\text{dist}} + \boldsymbol{\Delta E}^{\boldsymbol{AuX}}_{\text{dist}} \qquad \text{[S10]}$$

where "$\Delta E_{dist}{}^{CO2}$" represent the distortion penalty (or stabilization) due to CO_2 rearranging from its structure in the RC going into TSI and the "$\Delta E_{dist}{}^{comp}$" term represents the same distortion contribution concerning the rearrangement of the [$^{t}Bu_3PAuX$] complexes.

Since, for the sake of comparison, we analysed in the detail the electronic structure of intermediates INT, in order to exploit the factors controlling the different degrees of stabilization, we extended the ASM scheme as follows:

$$\boldsymbol{\Delta E^{\#}} = \left[\boldsymbol{\Delta E}^{\text{INT}}_{\text{dist}} - \boldsymbol{\Delta E}^{\boldsymbol{TSI}}_{\boldsymbol{dist}}\right] + \left[\boldsymbol{\Delta E}^{\text{INT}}_{\text{int}} - \boldsymbol{\Delta E}^{\boldsymbol{TSI}}_{\text{int}}\right] = \boldsymbol{\Delta\Delta E}_{\boldsymbol{dist}} + \boldsymbol{\Delta\Delta E}_{\text{int}} \qquad \text{[S11]}$$

$$\boldsymbol{\Delta\Delta E}_{\text{dist}} = \boldsymbol{E}^{\text{INT}}_{\boldsymbol{CO2}} - \boldsymbol{E}^{\boldsymbol{TSI}}_{\boldsymbol{CO2}} + \boldsymbol{E}^{\text{INT}}_{\boldsymbol{AuX}} - \boldsymbol{E}^{\text{TSI}}_{\boldsymbol{AuX}}$$
$$\boldsymbol{\Delta\Delta E}_{\text{dist}} = \boldsymbol{\Delta E}^{\boldsymbol{CO2}}_{\text{dist}} + \boldsymbol{\Delta E}^{\boldsymbol{AuX}}_{\text{dist}} \qquad \text{[S12]}$$

where the "$\Delta E_{dist}{}^{INT}$" term represents the energy penalty due to the distortion of the fragments (i.e. [LAuX] and CO_2) constrained in the structures of the intermediate (INT), whereas "$\Delta E_{int}{}^{INT}$" represents the interaction energy between the fragments (with the geometries constrained at the ones assumed in the INT).

	$[^tBu_3PAu]^{\cdot}$ - $[Al(NCCN')]^{\cdot}$	$[^tBu_3PAu]^{+}$- $[Al(NCCN')]^{-}$	$[^tBu_3PAu]^{-}$- $[Al(NCCN')]^{+}$
**ΔE_{Pauli}**	168.31	232.44	194.11
**ΔE_{Elst}**	-171.35	-295.76	-240.22
**ΔE_{Steric}**	-3.05	-63.31	-46.11
**ΔE_{oi}**	-73.34	-101.36	-181.26
**ΔE_{disp}**	-8.28	-8.28	-8.28
**ΔE**	-84.66	-172.95	-235.65

Table S1. Energy Decomposition Analysis (EDA) of the interaction energy between $[^tBu_3PAu]$ and [Al(NCCN')] fragments in complex $[^tBu_3PAu$**III**] using different fragmentations, i.e. doublet open shell neutral fragments (first column), $[^tBu_3PAu]^+$ singlet and $[Al(NCCN')]^-$ singlet fragments (second column) and $[^tBu_3PAu]^-$ singlet and $[Al(NCCN')]^+$ singlet fragments (third column). Energies are reported in kcal/mol.

	$[^tBu_3PAu]^{\cdot}$ - $[Al(^{Dipp}BDI\text{-}H')]^{\cdot}$	$[^tBu_3PAu]^{+}$- $[Al(^{Dipp}BDI\text{-}H')]^{-}$	$[^tBu_3PAu]^{-}$- $[Al(^{Dipp}BDI\text{-}H')]^{+}$
**ΔE_{Pauli}**	186.43	219.62	168.11
**ΔE_{Elst}**	-184.80	-284.18	-235.92
**ΔE_{Steric}**	1.63	-64.57	-67.82
**ΔE_{oi}**	-84.61	-96.06	-186.80
**ΔE_{disp}**	-7.76	-7.76	-7.76
**ΔE**	-90.74	-168.39	-262.37

Table S2. Energy Decomposition Analysis (EDA) of the interaction energy between $[^tBu_3PAu]$ and $[Al(^{Dipp}BDI\text{-}H')]$ fragments in complex $[^tBu_3PAu$**IV**] using different fragmentations, i.e. doublet open shell neutral fragments (first column), $[^tBu_3PAu]^+$ singlet and $[Al(^{Dipp}BDI\text{-}H')]^-$ singlet fragments (second column) and $[^tBu_3PAu]^-$ singlet and $[Al(^{Dipp}BDI\text{-}H')]^+$ singlet fragments (third column). Energies are reported in kcal/mol.

	$[^tBu_3PAu]\cdot$ - $[Al(CAA')]\cdot$	$[^tBu_3PAu]^+$- $[Al(CAA')]^-$	$[^tBu_3PAu]^-$- $[Al(CAA')]^+$
ΔE_{Pauli}	156.45	224.78	189.78
ΔE_{Elst}	-161.06	-288.00	-241.51
ΔE_{Steric}	-4.61	-63.21	-51.73
ΔE_{oi}	-72.08	-98.71	-184.46
ΔE_{disp}	-6.80	-6.80	-6.80
ΔE	-83.49	-168.73	-242.99

Table S3. Energy Decomposition Analysis (EDA) of the interaction energy between $[^tBu_3PAu]$ and [Al(CAA')] fragments in complex $[^tBu_3PAu$**V**] using different fragmentations, i.e. doublet open shell neutral fragments (first column), $[^tBu_3PAu]^+$ singlet and $[Al(CAA')]^-$ singlet fragments (second column) and $[^tBu_3PAu]^-$ singlet and $[Al(CAA')]^+$ singlet fragments (third column). Energies are reported in kcal/mol.

	$[^tBu_3PAu]\cdot$ - $[Al\{C(SiH_3)_2CH_2\}_2]\cdot$	$[^tBu_3PAu]^+$- $[Al\{C(SiH_3)_2CH_2\}_2]^-$	$[^tBu_3PAu]^-$- $[Al\{C(SiH_3)_2CH_2\}_2]^+$
ΔE_{Pauli}	155.71	214.56	148.11
ΔE_{Elst}	-160.60	-277.53	-217.39
ΔE_{Steric}	-4.89	-62.97	-69.28
ΔE_{oi}	-72.80	-97.64	-171.75
ΔE_{disp}	-5.61	-5.61	-5.61
ΔE	-83.30	-166.23	-246.64

Table S4. Energy Decomposition Analysis (EDA) of the interaction energy between $[^tBu_3PAu]$ and $[Al\{C(SiH_3)_2CH_2\}_2]$ fragments in complex $[^tBu_3PAu$**VI**] using different fragmentations, i.e. doublet open shell neutral fragments (first column), $[^tBu_3PAu]^+$ singlet and $[Al\{C(SiH_3)_2CH_2\}_2]^-$ singlet fragments (second column) and $[^tBu_3PAu]^-$ singlet and $[Al\{C(SiH_3)_2CH_2\}_2]^+$ singlet fragments (third column). Energies are reported in kcal/mol.

$\Delta\rho_{1\alpha}$' (Au-to-Al CT)

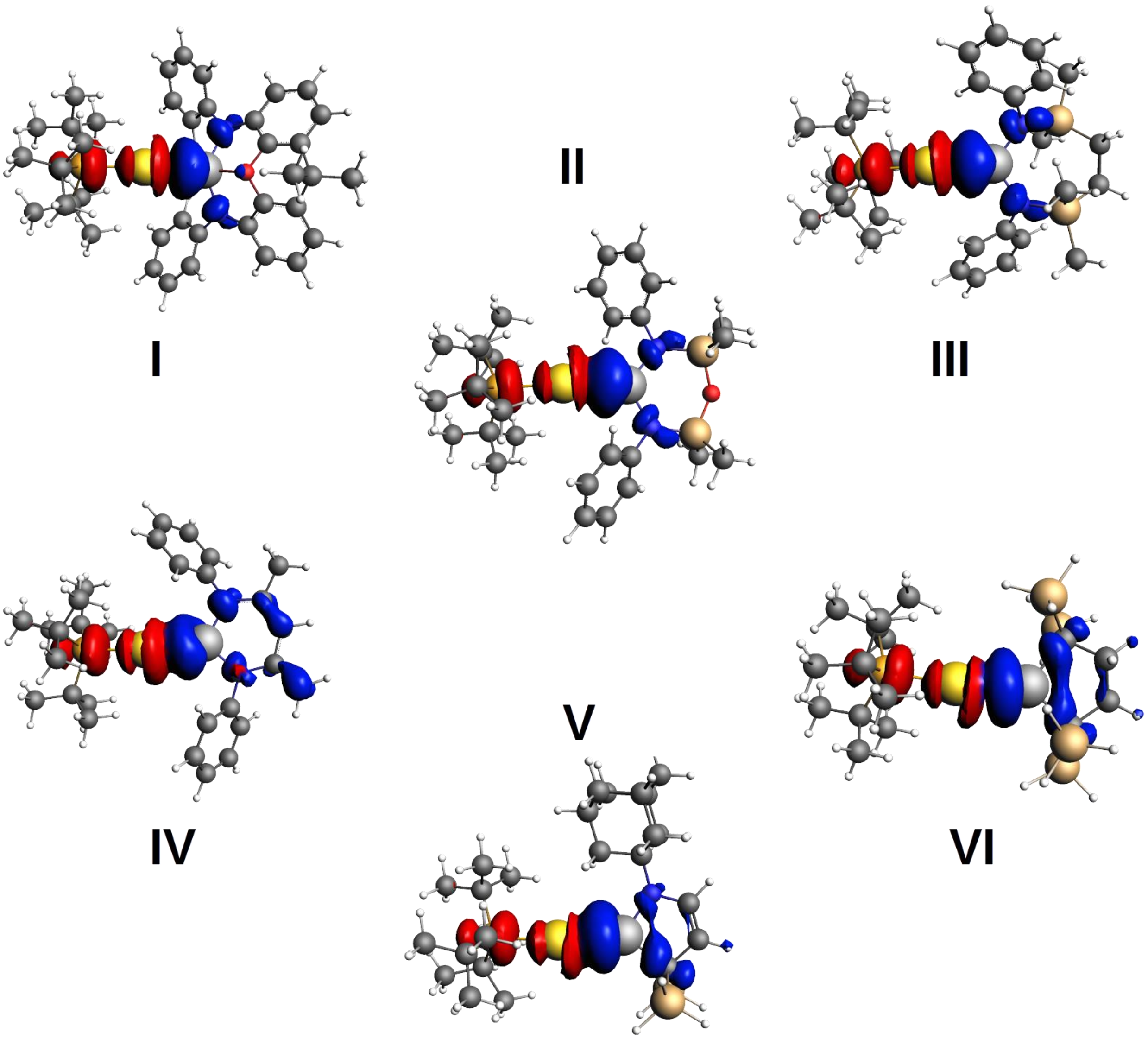

Figure S1. Isodensity surfaces of the $\Delta\rho_{1\alpha}$' NOCV deformation density for the interaction between doublet [^{t}Bu$_3$PAu]· and [X]· (X=Al(NON'), Al(^{Si}NON'), Al(NCCN'), Al(^{Dipp}BDI-H'), Al(CAA'), Al({C(SiH$_3$)$_2$CH$_2$}$_2$)) for complexes [^{t}Bu$_3$PAu**I**]-[^{t}Bu$_3$PAu**VI**]. Blue regions indicate electron density accumulation areas, whereas red regions indicate depletion areas. Isodensity value is 1 me/a_0^3.

$\Delta\rho_{1\beta}$' (Al-to-Au CT)

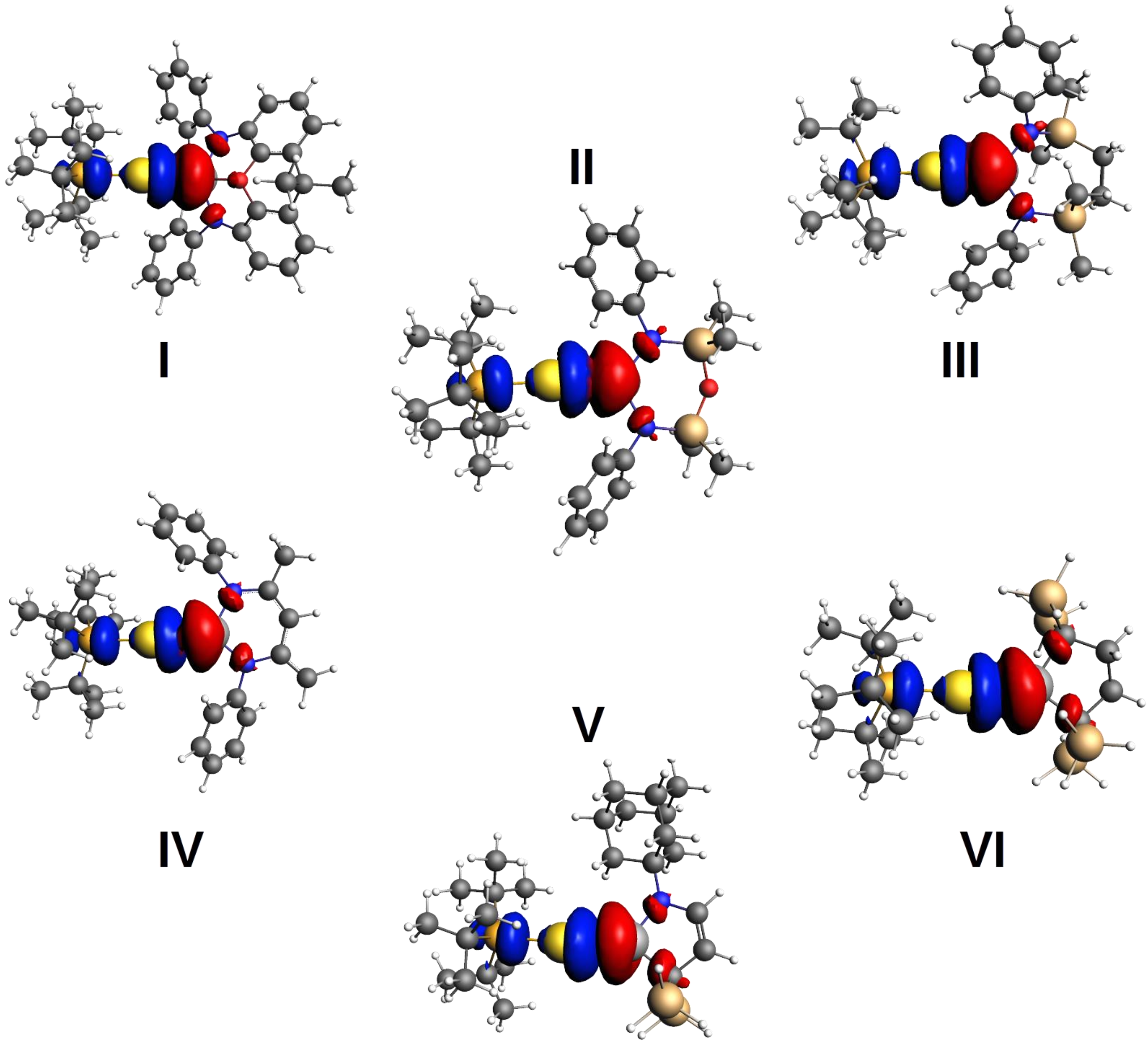

Figure S2. Isodensity surfaces of the $\Delta\rho_{1\beta}$' NOCV deformation density for the interaction between doublet [$^{t}Bu_3PAu$]· and [X]· (X=Al(NON'), Al(^{Si}NON'), Al(NCCN'), Al(^{Dipp}BDI-H'), Al(CAA'), Al({$C(SiH_3)_2CH_2$}$_2$)) for complexes [$^{t}Bu_3PAu$**I**]-[$^{t}Bu_3PAu$**VI**]. Blue regions indicate electron density accumulation areas, whereas red regions indicate depletion areas. Isodensity value is 1 me/a_0^3.

Δρ₂'

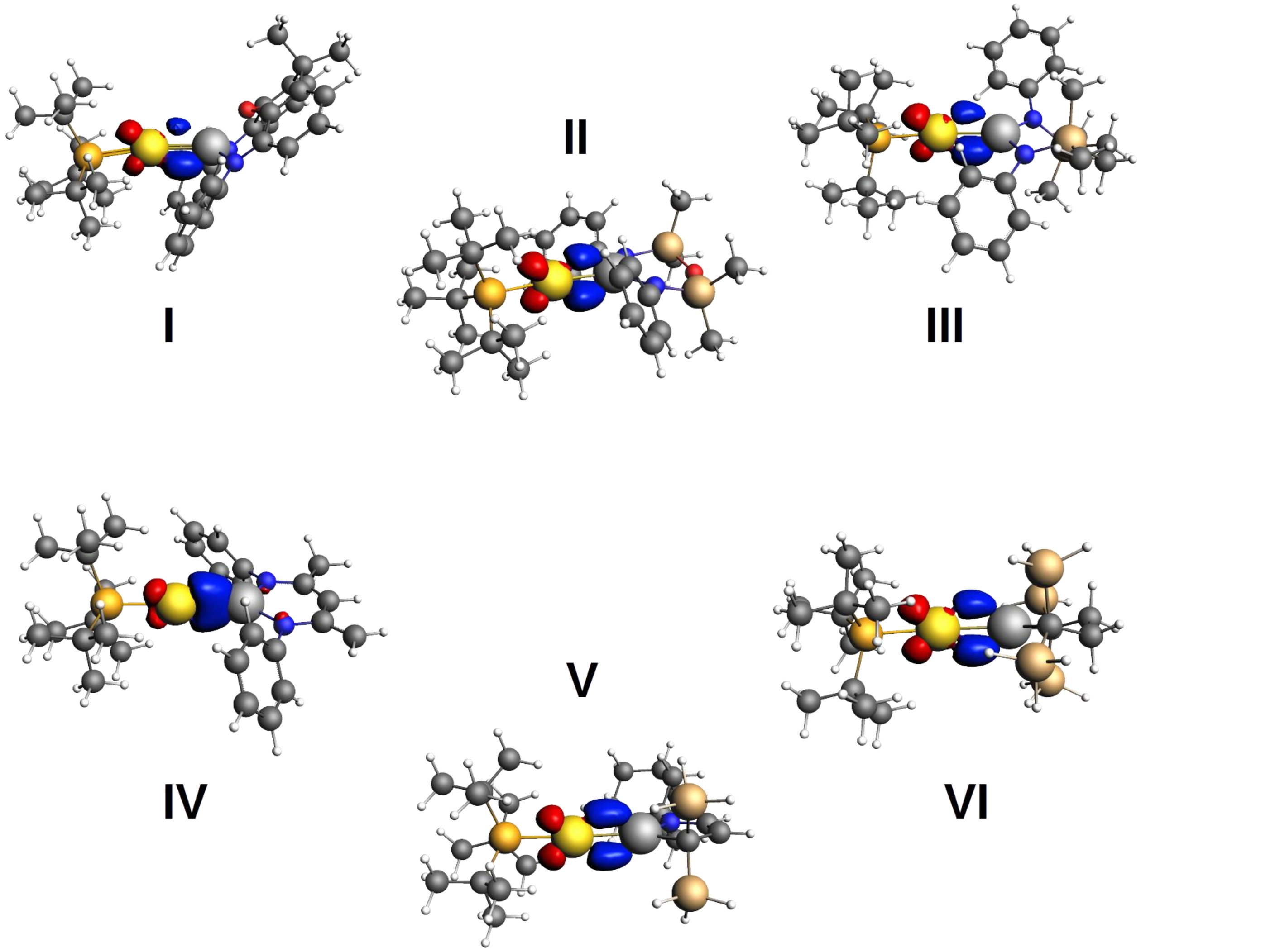

Figure S3. Isodensity surfaces of the $\Delta\rho_2$' NOCV deformation density for the interaction between doublet [^{t}Bu$_3$PAu]· and [X]· (X=Al(NON'), Al(^{Si}NON'), Al(NCCN'), Al(^{Dipp}BDI-H'), Al(CAA'), Al({C(SiH$_3$)$_2$CH$_2$}$_2$)) for complexes [^{t}Bu$_3$PAu**I**]-[^{t}Bu$_3$PAu**VI**]. Blue regions indicate electron density accumulation areas, whereas red regions indicate depletion areas. Isodensity value is 1 me/a_0^3.

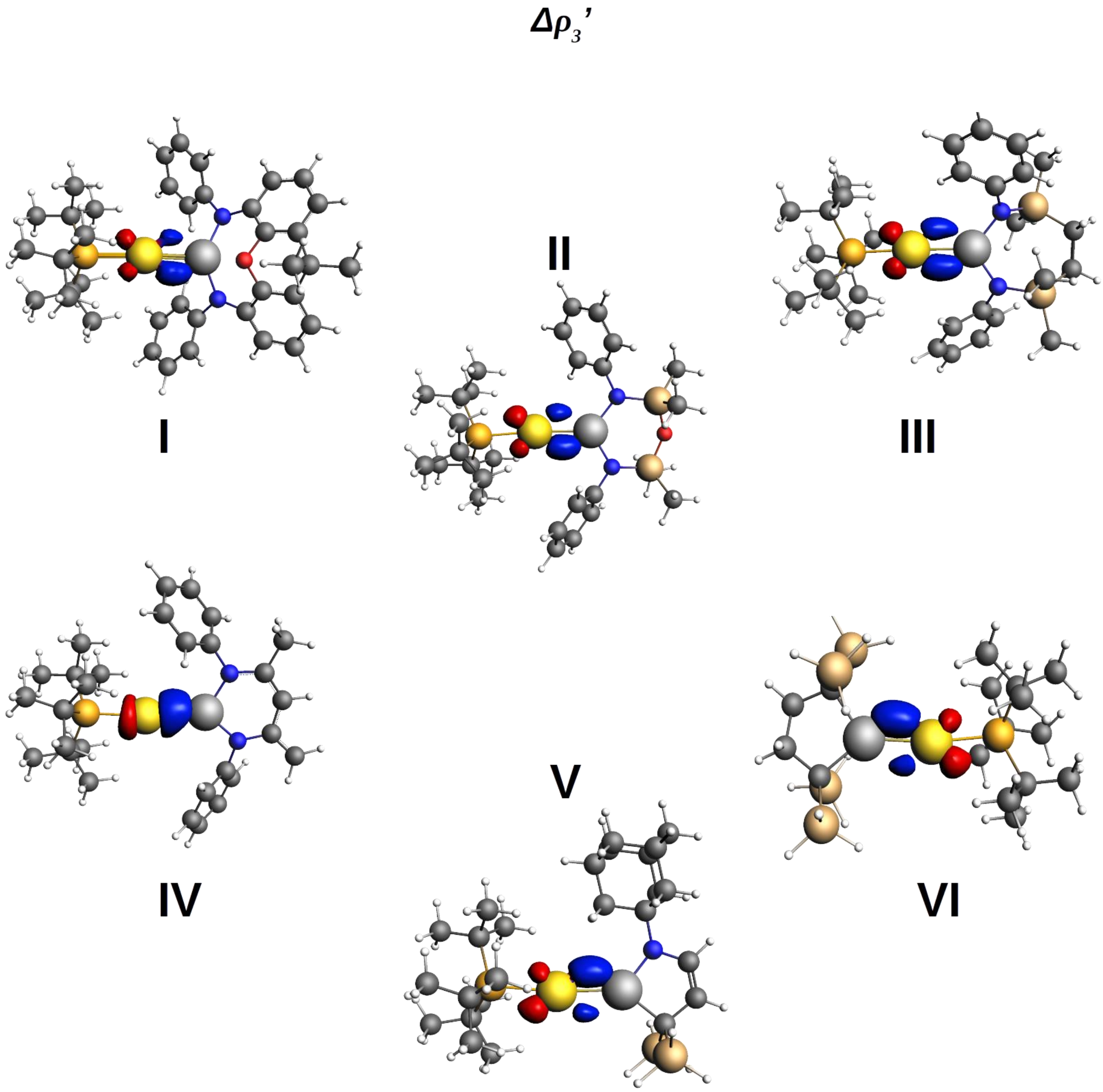

Figure S4. Isodensity surfaces of the *$\Delta\rho_3$'* NOCV deformation density for the interaction between doublet [^{t}Bu$_3$PAu]· and [X]· (X=Al(NON'), Al(^{Si}NON'), Al(NCCN'), Al(^{Dipp}BDI-H'), Al(CAA'), Al({C(SiH$_3$)$_2$CH$_2$}$_2$)) for complexes [^{t}Bu$_3$PAu**I**]-[^{t}Bu$_3$PAu**VI**]. Blue regions indicate electron density accumulation areas, whereas red regions indicate depletion areas. Isodensity value is 1 me/a$_0^3$.

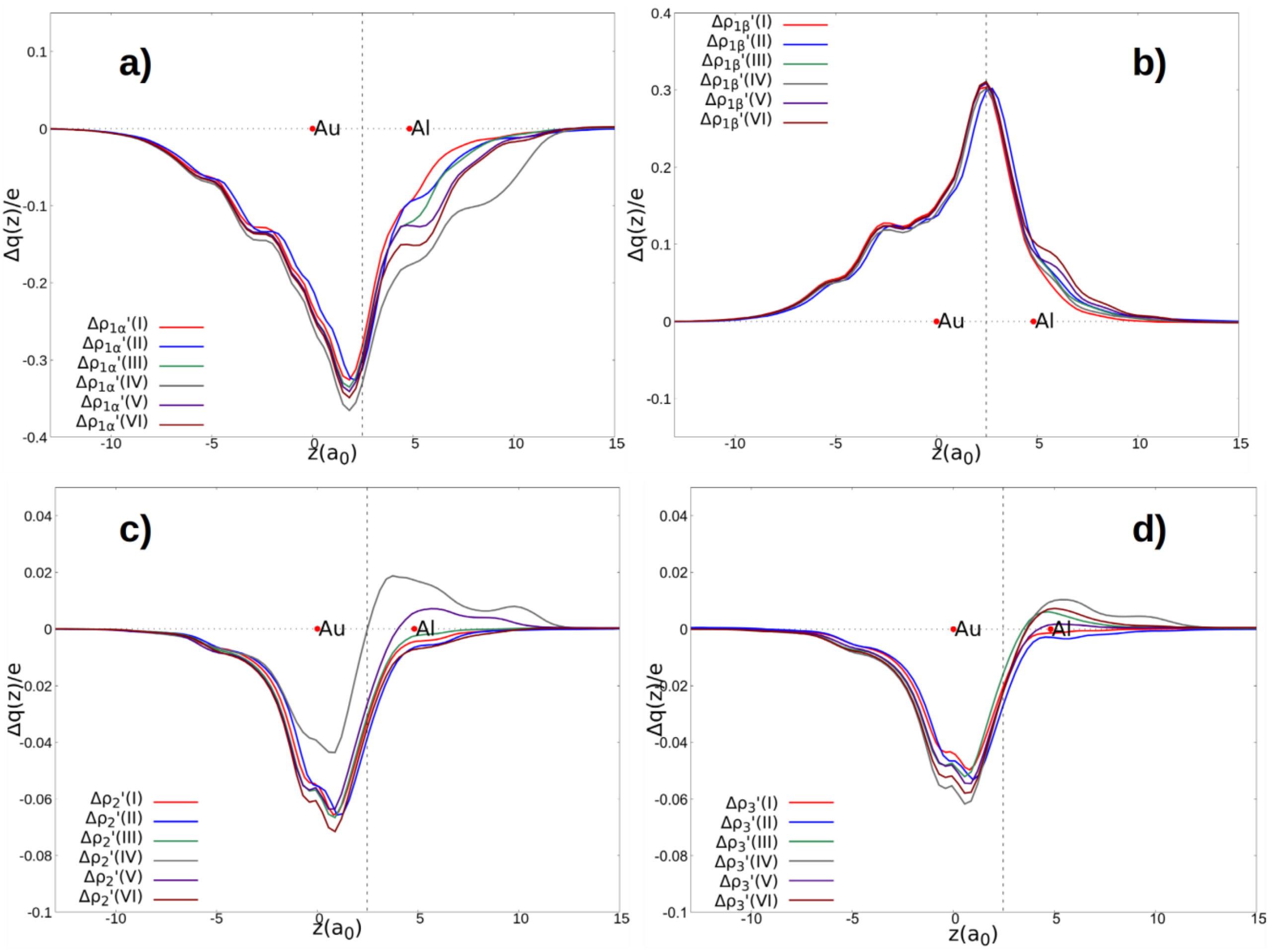

Figure S5. Charge Displacement (CD-NOCV) curves associated with the $\Delta\rho_{1\alpha}'$ (**a**), $\Delta\rho_{1\beta}'$ (**b**), $\Delta\rho_{2}'$ (**c**) *and* $\Delta\rho_{3}'$ (**d**) NOCV deformation densities for the interaction between doublet [^{t}Bu$_3$PAu]· and [X]· (X=Al(NON'), Al(^{Si}NON'), Al(NCCN'), Al(^{Dipp}BDI-H'), Al(CAA'), Al({C(SiH$_3$)$_2$CH$_2$}$_2$)) fragments for complexes [^{t}Bu$_3$PAu**I**]-[^{t}Bu$_3$PAu**VI**]. Red dots indicate the average position of the nuclei along the z axis. Positive (negative) values of the curve indicate right-to-left (left-to-right) charge transfer. The black dashed line indicates the average position of the isodensity boundary.

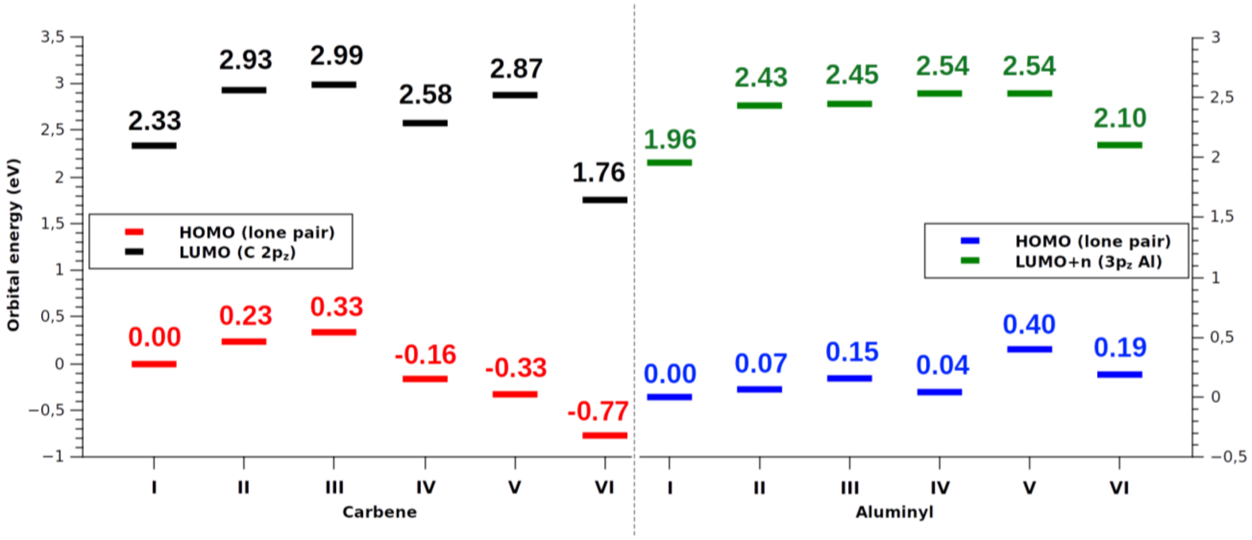

Figure S6. Calculated HOMO (associated with the lone pair on C/Al) and LUMO+n (associated with the vacant 2p/3p orbital on C/Al) energies for free carbenes **I**$^{\mathbf{C}}$-**VI**$^{\mathbf{C}}$ (left) and aluminyls **I-VI** (right). For carbenes, the 2p orbital of C is associated with the LUMO in all the cases, while for aluminyls the vacant 3p orbital of Al is associated with the LUMO (**VI**), LUMO+1 (**I**, **V**), LUMO+2 (**II**, **III**), LUMO+4 (**IV**). The energies (in eV) have been shifted in each case according to the HOMO energy of **I**$^{\mathbf{c}}$/**I**.

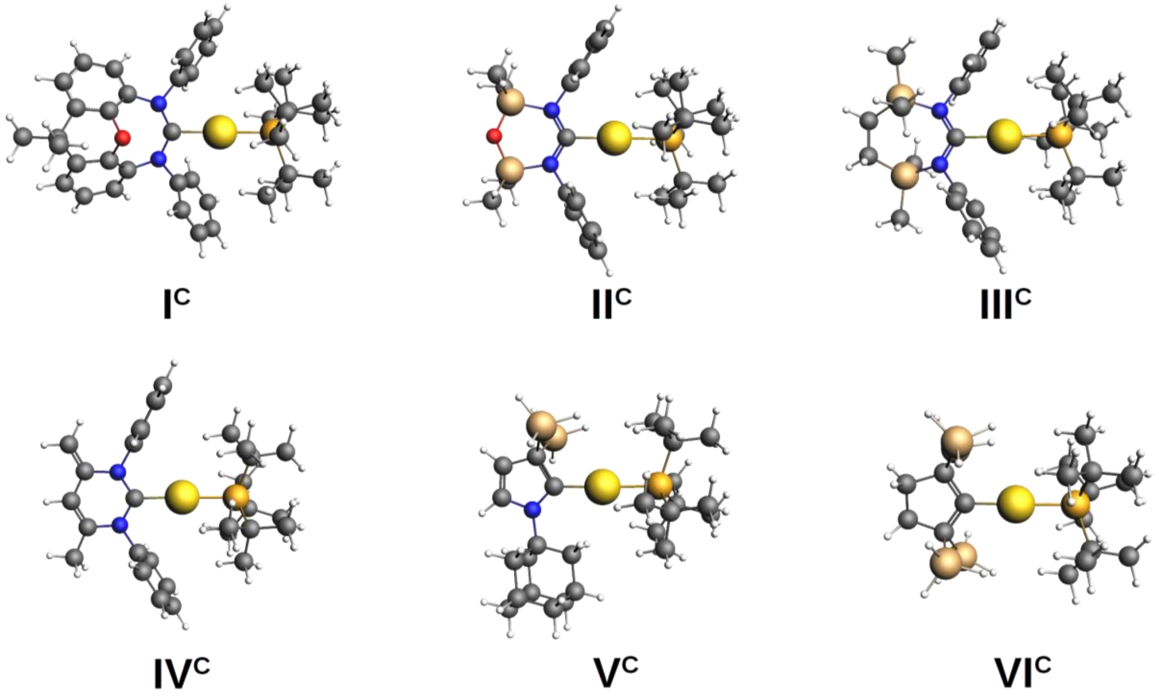

Figure S7. Optimized structures of [^{t}Bu$_3$PAuY]$^{+}$complexes (Y=**I**$^{\mathbf{C}}$-**VI**$^{\mathbf{C}}$).

	ΔE_{Pauli}	ΔE_{Elst}	ΔE_{Steric}	ΔE_{oi}	ΔE_{disp}	ΔE
**[^{t}Bu$_3$PAu]$^+$ - [I^C]**	179.6	-175.7	4.1	-82.0	-14.4	-92.4
**[^{t}Bu$_3$PAu]· - [I^C]$^{+\cdot}$**	213.7	-164.3	49.4	-173.0	-14.4	-138.0
**[^{t}Bu$_3$PAu]$^+$ - [II^C]**	194.2	-197.2	-3.0	-82.6	-13.4	-99.1
**[^{t}Bu$_3$PAu]· - [II^C]$^{+\cdot}$**	210.1	-168.7	41.4	-154.6	-13.4	-126.6
**[^{t}Bu$_3$PAu]$^+$ - [III^C]**	200.0	-203.1	-3.1	-84.2	-14.0	-101.3
**[^{t}Bu$_3$PAu]· - [III^C]$^{+\cdot}$**	221.4	-172.7	48.6	-155.1	-14.0	-120.5
**[^{t}Bu$_3$PAu]$^+$ - [IV^C]**	189.3	-188.9	0.4	-78.9	-12.7	-91.2
**[^{t}Bu$_3$PAu]· - [IV^C]$^{+\cdot}$**	276.0	-201.1	74.8	-191.0	-12.7	-128.9
**[^{t}Bu$_3$PAu]$^+$ - [V^C]**	205.4	-202.4	3.0	-83.0	-10.7	-90.7
**[^{t}Bu$_3$PAu]· - [V^C]$^{+\cdot}$**	225.0	-176.7	48.3	-174.8	-10.7	-137.2
**[^{t}Bu$_3$PAu]$^+$ - [VI^C]**	219.7	-214.7	5.0	-85.4	-9.6	-90.0
**[^{t}Bu$_3$PAu]· - [VI^C]$^{+\cdot}$**	213.8	-173.6	40.1	-165.9	-9.6	-135.4

Table S5. Energy Decomposition Analysis (EDA) of the interaction energy between [^{t}Bu$_3$PAu] and [Y] (Y=**I^C**-**VI^C**) fragments in complexes [^{t}Bu$_3$PAu**I^C**]$^+$-[^{t}Bu$_3$PAu**VI^C**]$^+$ using different fragmentations, *i.e.* closed shell [^{t}Bu$_3$PAu]$^+$ and [Y] singlet fragments and doublet open shell [^{t}Bu$_3$PAu]· and [Y]$^{+\cdot}$ fragments. Energies are reported in kcal/mol.

Complex	I^C	II^C	III^C	IV^C	V^C	VI^C
ΔE_{oi}^3	-4.7	-6.5	-6.7	-6.4	-7.3	-8.3
CT^3	-0.014	-0.025	-0.026	-0.025	-0.029	-0.037
ΔE_{oi}^4	-4.2	-3.4	-3.4	-3.8	-4.4	-4.6
CT^4	-0.005	-0.001	-0.005	-0.010	-0.010	-0.010
CT^{net}	0.292	0.290	0.298	0.264	0.304	0.270

Table S6. Orbital interaction energies (ΔE_{oi}^k) and charge transfer (CT^k) associated with the third and fourth NOCV deformation densities for the interaction between closed-shell $[^tBu_3PAu]^+$ and [Y] fragments (Y=C(NON'), C(^{Si}NON'), C(NCCN'), C(^{Dipp}BDI-H'), C(CAA'), C({C(SiH$_3$)$_2$CH$_2$}$_2$) for complexes $[^tBu_3PAu\mathbf{I^C}]^+$-$[^tBu_3PAu\mathbf{VI^C}]^+$. Energies are reported in kcal/mol, CT values are reported in electrons.

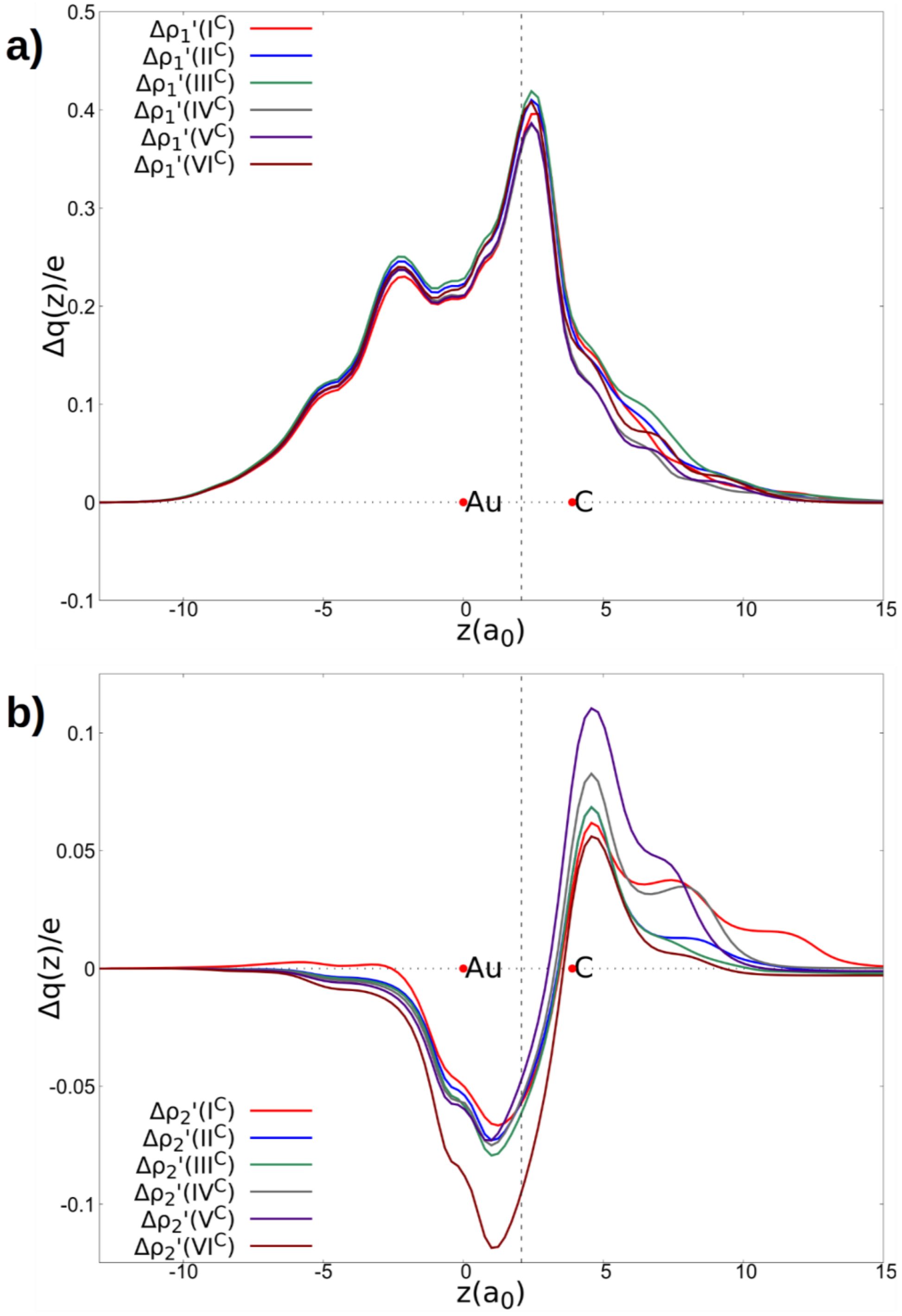

Figure S8. Charge Displacement (CD-NOCV) curves associated with the *Δρ₁'* (**a**) and *Δρ₂'* (**b**) NOCV deformation densities for the interaction between singlet [^{t}Bu$_3$PAu]$^+$ and [Y] (Y=C(NON'), C(^{Si}NON'), C(NCCN'), C(^{Dipp}BDI-H'), C(CAA'), C({C(SiH$_3$)$_2$CH$_2$}$_2$)) fragments for complexes [^{t}Bu$_3$PAu**I**$^{\mathbf{C}}$]$^+$-[^{t}Bu$_3$PAu**VI**$^{\mathbf{C}}$]$^+$. Red dots indicate the average position of the nuclei along the z axis. Positive (negative) values of the curve indicate right-to-left (left-to-right) charge transfer. The black dashed line indicates the average position of the isodensity boundary.

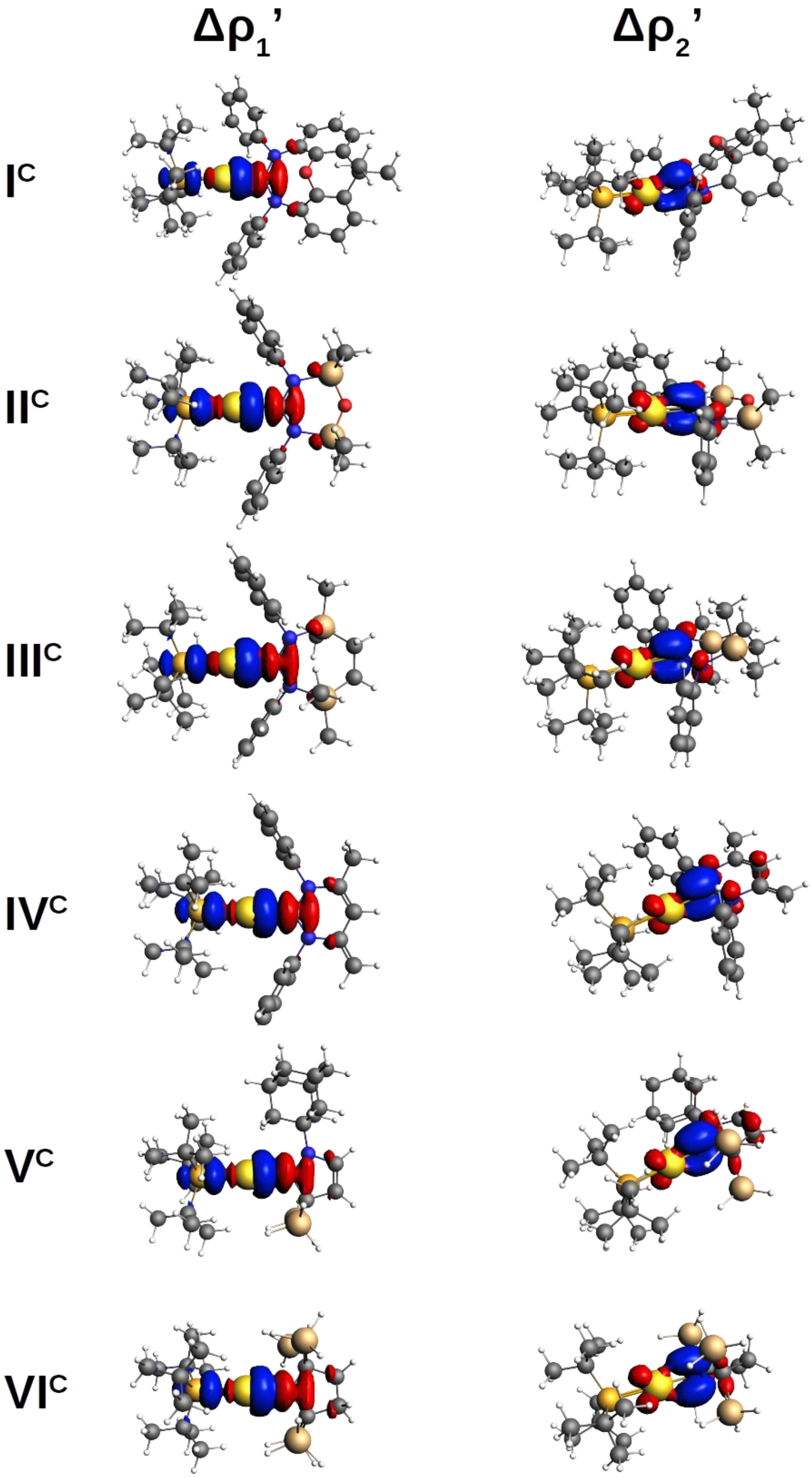

Figure S9. Isodensity surfaces of the *Δρ₁'* and *Δρ₂'* NOCV deformation densities for the interaction between singlet [$^{t}Bu_3PAu]^+$ and [Y] (Y=C(NON'), C(^{Si}NON'), C(NCCN'), C(^{Dipp}BDI-H'), C(CAA'), C({C($SiH_3)_2CH_2\}_2$)) fragments for complexes [$^{t}Bu_3PAu$**I**$^{C}]^+$-[$^{t}Bu_3PAu$**VI**$^{C}]^+$. Blue regions indicate electron density accumulation areas, whereas red regions indicate depletion areas. Isodensity value is 2 me/a_0^3 for *$\Delta\rho_1$'* and 1 me/a_0^3 for *$\Delta\rho_2$'*.

	$[^tBu_3PAu]^+$- $[Si\{C(SiH_3)_2CH_2\}_2]$	$[^tBu_3PAu]\cdot$ - $[Si\{C(SiH_3)_2CH_2\}_2]^{+\cdot}$
**ΔE_{Pauli}**	174.14	173.31
**ΔE_{Elst}**	-163.61	-159.94
**ΔE_{Steric}**	10.53	13.38
**ΔE_{oi}**	-79.63	-129.75
**ΔE_{disp}**	-7.44	-7.44
**ΔE**	-76.54	-123.81

Table S7. Energy Decomposition Analysis (EDA) of the interaction energy between $[^tBu_3PAu]$ and $[Si\{C(SiH_3)_2CH_2\}_2]$ fragments in complex $[^tBu_3PAuSi\{C(SiH_3)_2CH_2\}_2]^+$ using different fragmentations, i.e. $[^tBu_3PAu]^+$ singlet and $[Si\{C(SiH_3)_2CH_2\}_2]$ fragments (first column) and doublet open shell $[^tBu_3PAu]\cdot$ and $[Si\{C(SiH_3)_2CH_2\}_2]^{\cdot+}$ fragments (second column). Energies are reported in kcal/mol.

	$[^tBu_3PAu]^+$- $[B\{C(SiH_3)_2CH_2\}_2]^-$	$[^tBu_3PAu]\cdot$ - $[B\{C(SiH_3)_2CH_2\}_2]\cdot$
**ΔE_{Pauli}**	293.22	215.02
**ΔE_{Elst}**	-365.49	-204.40
**ΔE_{Steric}**	-72.28	10.62
**ΔE_{oi}**	-113.66	-100.47
**ΔE_{disp}**	-8.54	-8.54
**ΔE**	-194.48	-98.39

Table S8. Energy Decomposition Analysis (EDA) of the interaction energy between $[^tBu_3PAu]$ and $[B\{C(SiH_3)_2CH_2\}_2]$ fragments in complex $[^tBu_3PAuB\{C(SiH_3)_2CH_2\}_2]$ using different fragmentations, i.e. $[^tBu_3PAu]^+$ singlet and $[B\{C(SiH_3)_2CH_2\}_2]^-$ fragments (first column) and doublet open shell $[^tBu_3PAu]\cdot$ and $[B\{C(SiH_3)_2CH_2\}_2]\cdot$ fragments (second column). Energies are reported in kcal/mol.

	**I**	**II**	**III**	**IV**	**V**	**VI**
**% Al 3s**	59.3	59.0	57.8	62.9	47.7	40.4
**% Al 3p$_z$**	18.8	22.4	21.1	20.0	28.1	29.5
**%Al total**	78.1	81.4	78.9	82.9	75.8	69.9
**ΔE^{PA} (kcal/mol)**	-362.1	-354.5	-355.2	-349.6	-353.3	-349.4
	**I^C**	**II^C**	**III^C**	**IV^C**	**V^C**	**VI^C**
**% C 2s**	23.4	23.3	26.7	12.5	27.1	50.3
**% C 2p$_z$**	43.8	35.7	42.6	41.3	49.1	18.4
**%C total**	67.2	59.0	69.3	53.8	76.2	68.7
**ΔE^{PA} (kcal/mol)**	-282.9	-287.7	-288.2	-301.3	-277.5	-267.9

Table S9. Contributions from Al/C ns and np$_z$ (n=2,3) atomic orbitals to the HOMOs of isolated aluminyls **I-VI** and carbenes **I^C-VI^C**, respectively. The proton affinity (ΔE^{PA}) for each species is also reported.

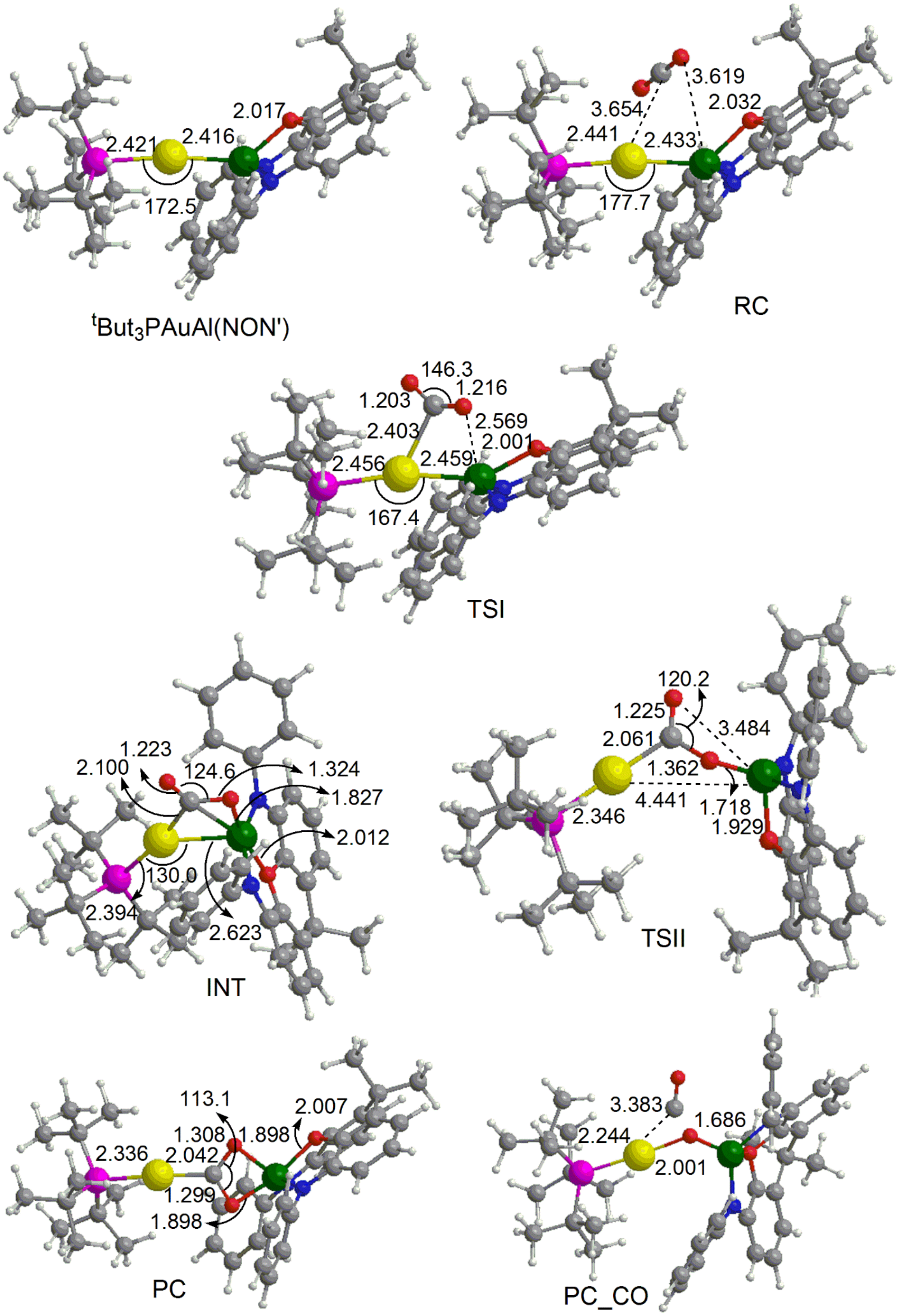

Figure S10. Optimized structures of [^{t}Bu$_3$PAuAl(NON')] (**I**), RC, TSI, INT, TSII, PC and PC_CO complexes. Main geometrical parameters are reported (bond in Å, angles in degree).

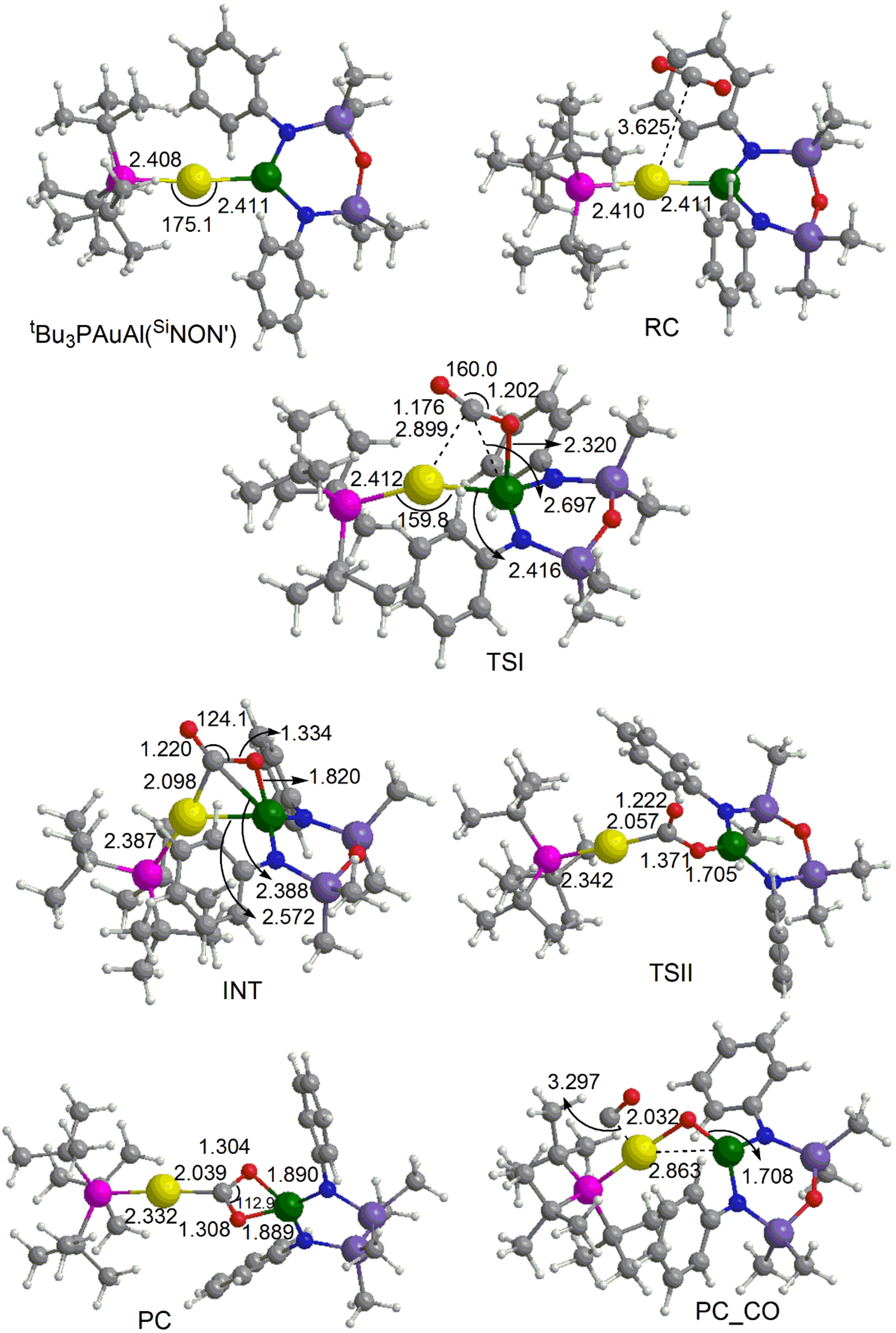

Figure S11. Optimized structures of [$^{t}Bu_3PAuAl(^{Si}NON')$] (**II**), RC, TSI, INT, TSII, PC and PC_CO complexes. Main geometrical parameters are reported (bond in Å, angles in degree).

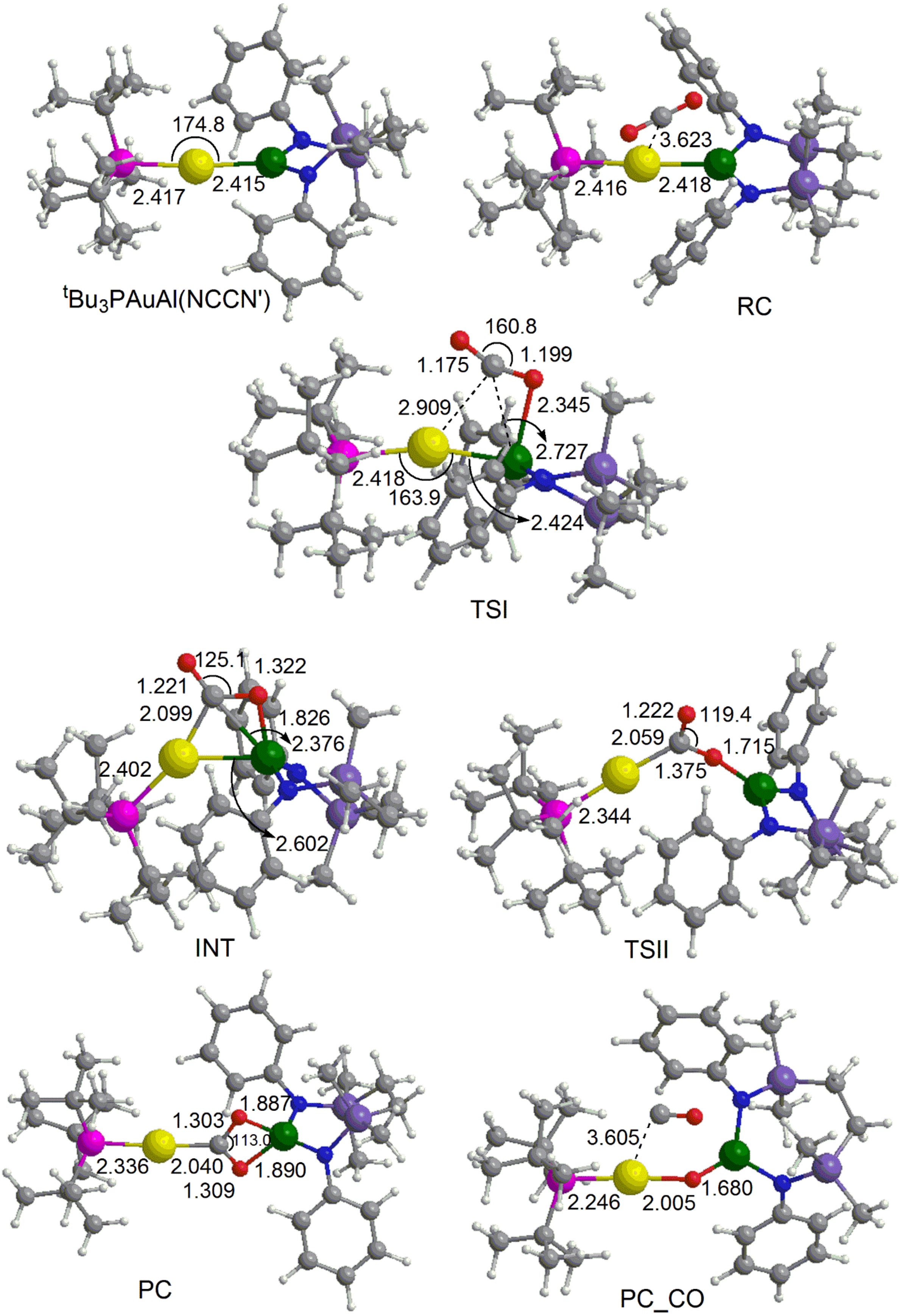

Figure S12. Optimized structures of [$^{t}Bu_3PAuAl(NCCN')$] (**III**), RC, TSI, INT, TSII, PC and PC_CO complexes. Main geometrical parameters are reported (bond in Å, angles in degree).

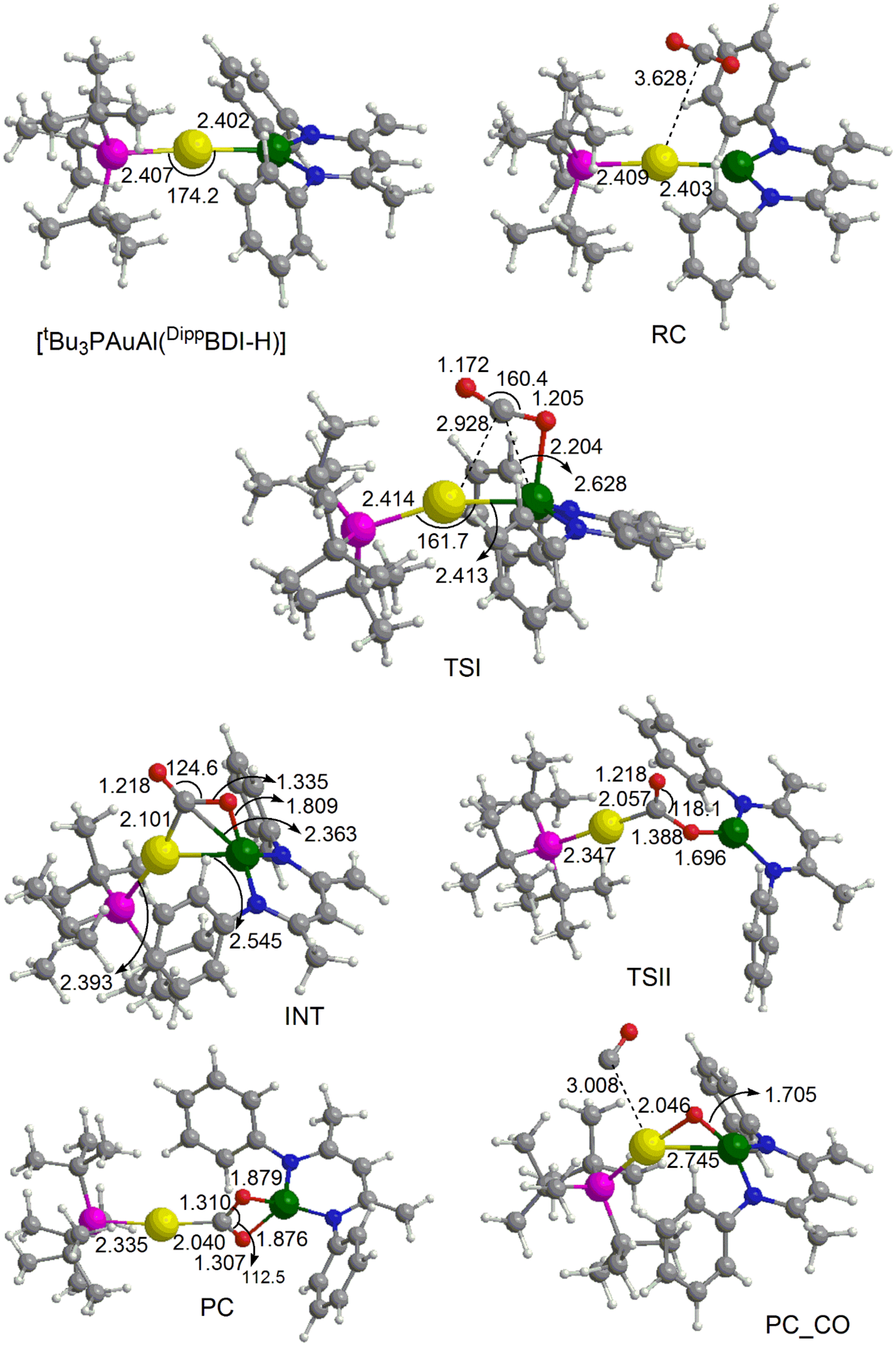

Figure S13. Optimized structures of [^{t}Bu$_3$PAuAl(^{Dipp}BDI-H')] (**IV**), RC, TSI, INT, TSII, PC and PC_CO complexes. Main geometrical parameters are reported (bond in Å, angles in degree).

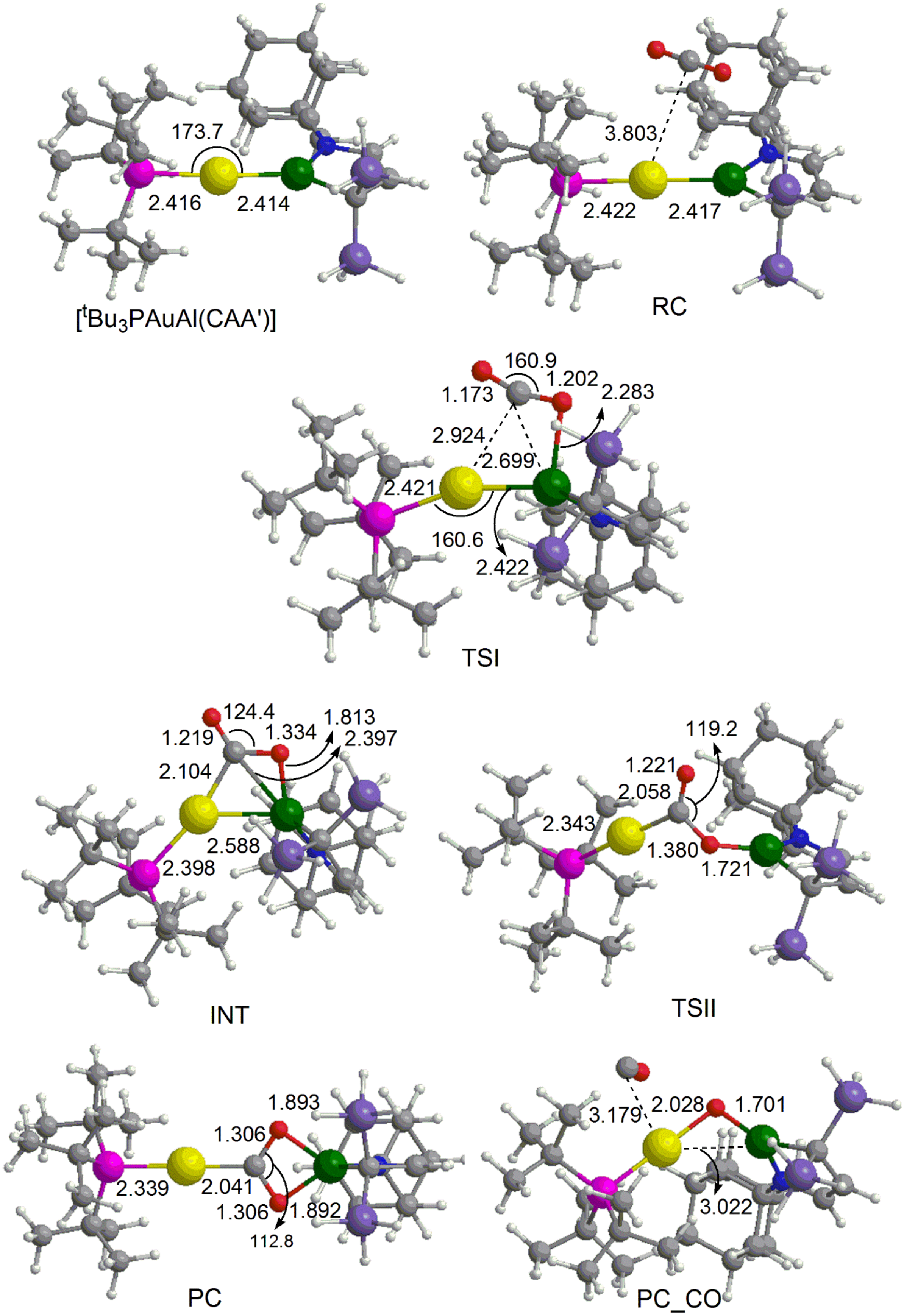

Figure S14. Optimized structures of [^{t}Bu$_3$PAuAl(CAA')] (**V**), RC, TSI, INT, TSII, PC and PC_CO complexes. Main geometrical parameters are reported (bond in Å, angles in degree).

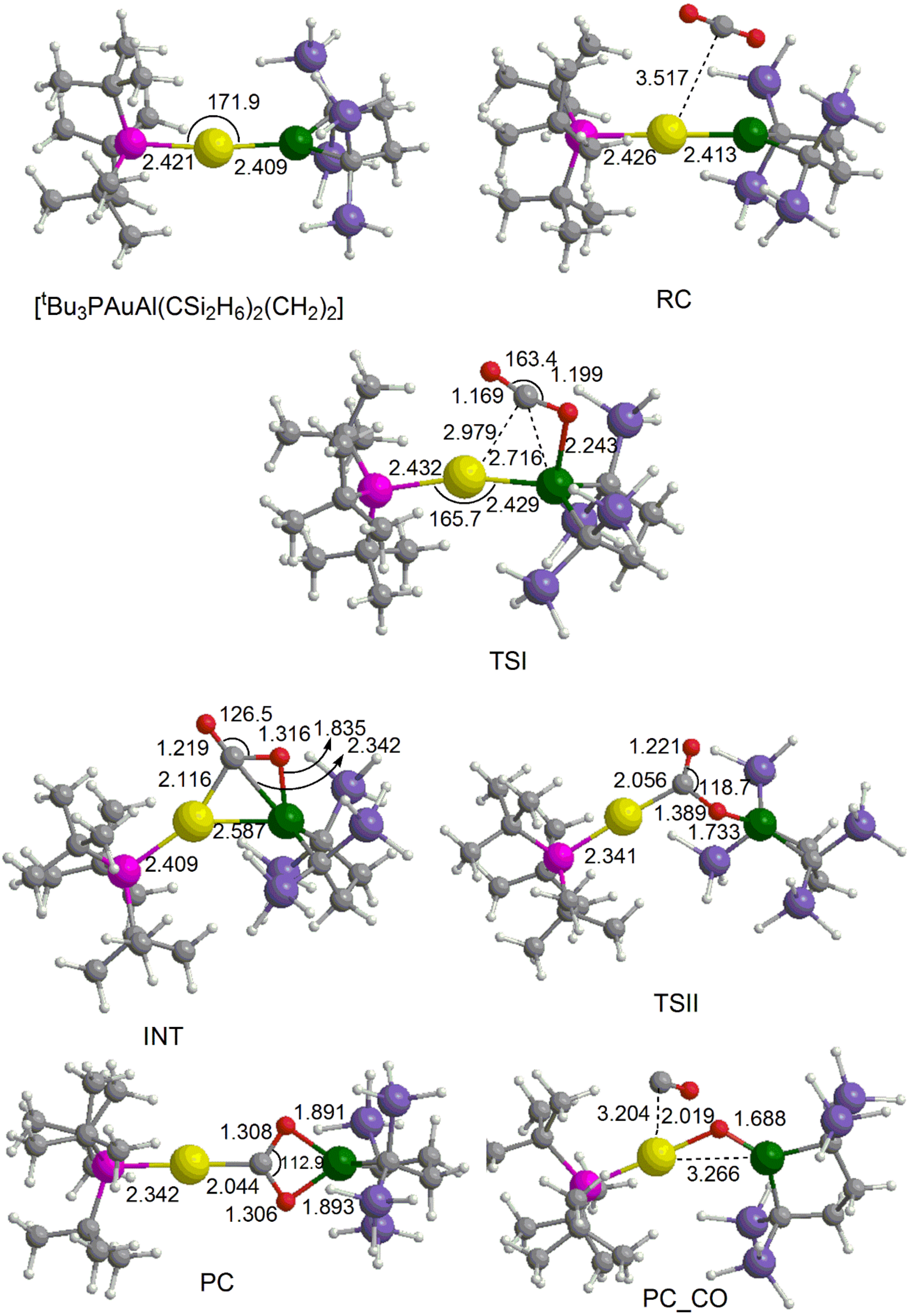

Figure S15. Optimized structures of [$^{t}Bu_3PAuAl\{(C(SiH_3)_2CH_2)_2\}$] (**VI**), RC, TSI, INT, TSII, PC and PC_CO complexes. Main geometrical parameters are reported (bond in Å, angles in degree).

	I	II	III	IV	V	VI
**$\Delta E^{\#}$**	9.0	5.6	4.8	6.1	5.0	1.4
**ΔE_{INT}^{RC}**	-5.1	-5.8	-5.7	-5.0	-4.3	-5.2
**ΔE_{INT}^{TS}**	-18.0	-8.5	-8.5	-7.7	-7.1	-9.4
**$\Delta\Delta E_{INT}$**	-12.9	-2.7	-2.8	-2.7	-2.8	-4.2
**ΔE_{DIST}^{CO2}**	20.3	7.1	6.5	7.1	6.6	5.0
**ΔE_{DIST}^{comp}**	1.6	1.2	1.1	1.7	1.2	0.6
**$\Delta\Delta E_{DIST}$**	21.9	8.3	7.6	8.8	7.8	5.6

Table S10. Results of the Activation Strain Model (ASM) analysis of the [CO_2]-[^{t}Bu$_3$PAuX] (X=Al(NON'), Al(^{Si}NON'), Al(NCCN'), Al(^{Dipp}BDI-H'), Al(CAA'), Al({C(SiH$_3$)$_2$CH$_2$}$_2$)) interaction for the electronic energy activation barrier involving complexes [^{t}Bu$_3$PAu**I**]-[^{t}Bu$_3$PAu**VI**]. All energies are expressed in kcal/mol.

	I	II	III	IV	V	VI
**ΔE^{INT}**	-12.0	-17.9	-18.4	-16.7	-16.0	-16.6
**ΔE_{INT}^{RC}**	-5.1	-5.8	-5.7	-5.0	-4.3	-5.2
**ΔE_{INT}^{INT}**	-107.0	-106.7	-100.0	-101.9	-102.1	-93.1
**$\Delta\Delta E_{INT}$**	-101.9	-100.9	-94.3	-96.9	-97.8	-87.9
**ΔE_{DIST}^{CO2}**	64.6	66.8	63.3	66.0	66.2	59.9
**ΔE_{DIST}^{comp}**	25.3	16.2	12.6	14.4	15.6	11.4
**$\Delta\Delta E_{DIST}$**	89.9	83.0	75.9	80.2	81.8	71.3

Table S11. Results of the Activation Strain Model (ASM) analysis of the [CO_2]-[^{t}Bu$_3$PAuX] (X=Al(NON'), Al(^{Si}NON'), Al(NCCN'), Al(^{Dipp}BDI-H'), Al(CAA'), Al({C(SiH$_3$)$_2$CH$_2$}$_2$)) interaction for the INT stabilization involving complexes [^{t}Bu$_3$PAu**I**]-[^{t}Bu$_3$PAu**VI**]. All energies are expressed in kcal/mol.

	I	II	III	IV	V	VI
**ΔE_{Pauli}**	102.7	65.4	62.2	75.5	69.7	60.5
**ΔE_{elst}**	-60.3	-42.3	-40.9	-47.8	-45.0	-39.8
**ΔE_{Steric}**	42.4	23.1	21.4	27.6	24.7	20.7
**ΔE_{oi}**	-55.8	-28.4	-26.4	-32.4	-28.8	-27.3
**ΔE_{oi}^1**	-41.2	-18.2	-16.7	-20.4	-17.8	-15.9
**ΔE_{oi}^2**	-4.0	-5.4	-5.0	-6.2	-5.6	-5.9
**ΔE_{disp}**	-5.5	-4.7	-5.1	-4.4	-4.4	-4.6
**ΔE**	-18.9	-10.0	-10.1	-9.3	-8.5	-11.1

Table S12. Results of the Energy Decomposition Analysis (EDA) of the [CO_2]-[$^tBu_3AuX$] (X=Al(NON’), Al(^{Si}NON’), Al(NCCN’), Al(^{Dipp}BDI-H’), Al(CAA’), Al({$C(SiH_3)_2CH_2$}$_2$)) interaction for the TSI involving complexes [$^tBu_3PAu$**I**]-[$^tBu_3PAu$**VI**]. All energies are expressed in kcal/mol.

	I	II	III	IV	V	VI
**ΔE_{Pauli}**	390.6	406.2	384.2	405.7	388.1	356.8
**ΔE_{elst}**	-227.3	-234.3	-222.3	-232.4	-222.9	-207.1
**ΔE_{Steric}**	163.3	171.9	161.9	173.3	165.2	149.7
**ΔE_{oi}**	-262.9	-272.9	-256.2	-270.4	-262.0	-238.1
**ΔE_{oi}^1**	-215.8	-225.3	-209.4	-222.8	-214.8	-191.9
**$\lvert CT^1\rvert$**	0.66	0.67	0.65	0.65	0.66	0.64
**ΔE_{oi}^2**	-10.8	-11.1	-11.4	-9.7	-10.5	-12.2
**$\lvert CT^2\rvert$**	0.07	0.07	0.07	0.07	0.07	0.07
**ΔE_{disp}**	-5.7	-4.9	-4.9	-4.5	-4.3	-4.3
**ΔE**	-105.3	-105.9	-99.2	-101.6	-101.1	-92.8

Table S13. Results of the Energy Decomposition Analysis (EDA) of the [CO_2]-[$^tBu_3AuX$] (X=Al(NON’), Al(^{Si}NON’), Al(NCCN’), Al(^{Dipp}BDI-H’), Al(CAA’), Al({$C(SiH_3)_2CH_2$}$_2$)) interaction for the INT involving complexes [$^tBu_3PAu$**I**]-[$^tBu_3PAu$**VI**]. All energies are expressed in kcal/mol.

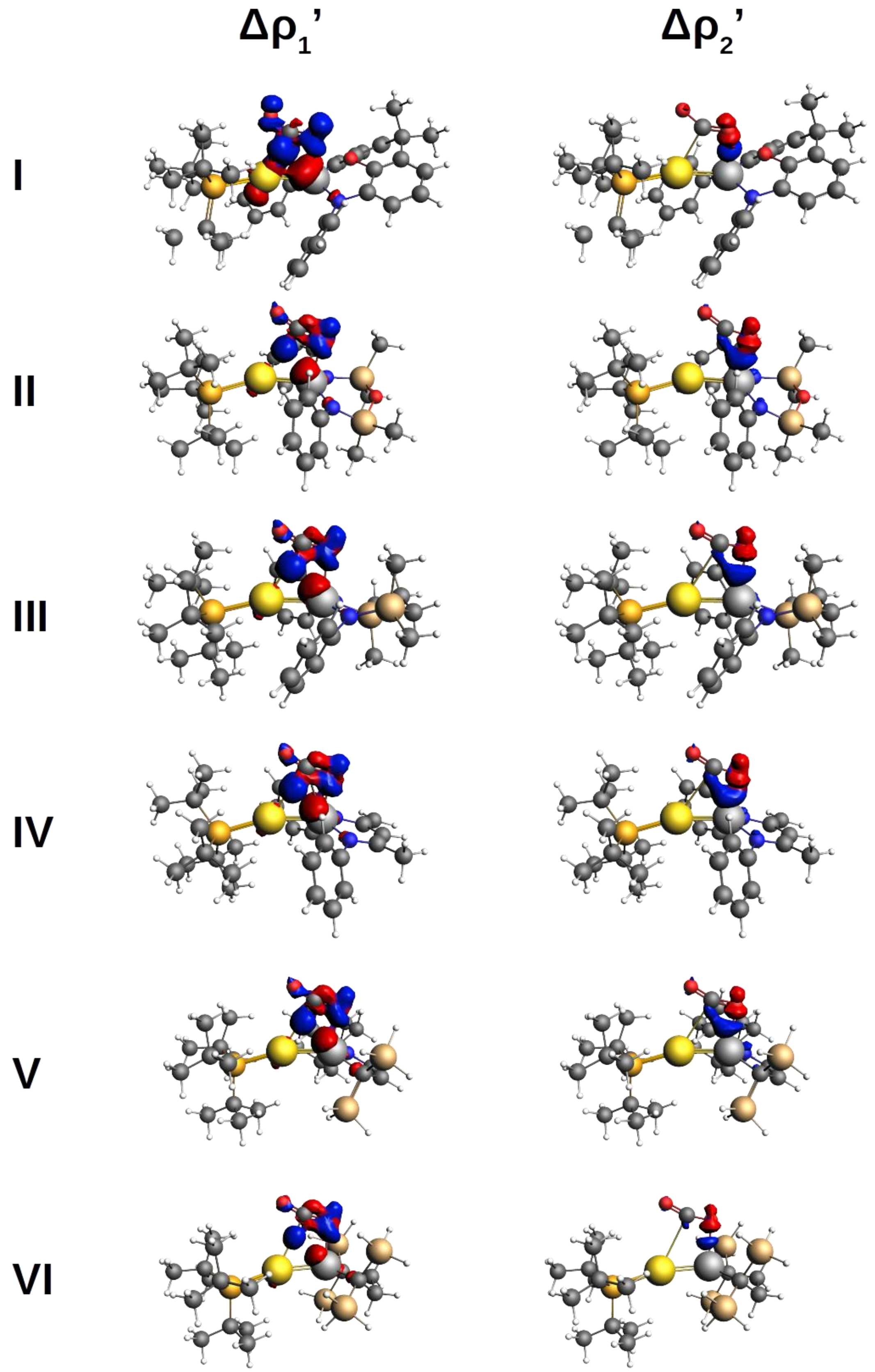

Figure S16. Isodensity surfaces of the *Δρ1'* and *Δρ2'* NOCV deformation densities for the $[CO_2]$-$[^tBu_3AuX]$ (X=Al(NON'), Al(^{Si}NON'), Al(NCCN'), Al(^{Dipp}BDI-H'), Al(CAA'), Al($\{C(SiH_3)_2CH_2\}_2$)) interaction for the TSI involving complexes [^{t}Bu$_3$PAu**I**]-[^{t}Bu$_3$PAu**VI**]. Blue regions indicate electron density accumulation areas, whereas red regions indicate depletion areas. Isovalue for all the surfaces is 2 me/a$_0$.

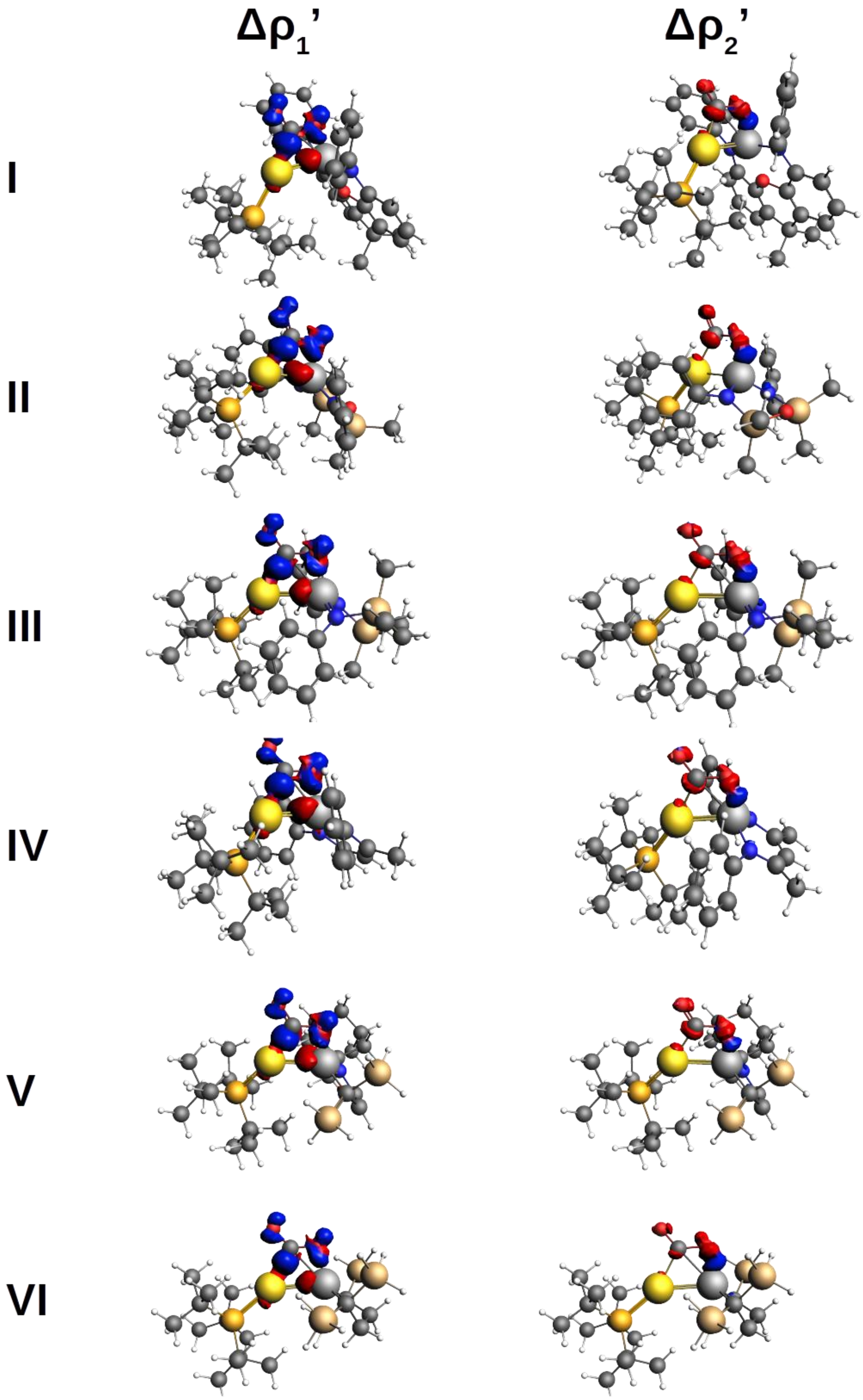

Figure S17. Isodensity surfaces of the $\Delta\rho_1$' and $\Delta\rho_2$' NOCV deformation densities for the $[CO_2]$-[$^tBu_3AuX$] (X=Al(NON'), Al(^{Si}NON'), Al(NCCN'), Al(^{Dipp}BDI-H'), Al(CAA'), Al({$C(SiH_3)_2CH_2$}$_2$)) interaction for the INT involving complexes [$^tBu_3PAu$**I**]-[$^tBu_3PAu$**VI**]. Blue regions indicate electron density accumulation areas, whereas red regions indicate depletion areas. Isovalues for the surfaces are 8 me/a_0 for $\Delta\rho_1$' and 3 me/a_0 for $\Delta\rho_2$'.

Modelling of the ligand

The choice of using slightly simplified structures for anions **I-VI** was made for achieving the best compromise between computational cost and accuracy. Surely we do expect an influence of the modified steric hindrance of the ligand on the actual reactivity (as we also mention in the text). However, we also expect much smaller deviations in terms of the electronic effects (which are the focus of our work) induced by such modifications. On this matter, we mention that in our previous work on gold complex with **I**,[16] we showed that the simplified geometry optimized at the PBE/TZ2P level is comparable to the non-simplified (real) one optimized at the same level of theory and also in good agreement with the experimentally determined one via X-Ray diffraction.[17]

Nonetheless, we recognize that the effect of such structural simplifications on the calculations we carried out (particularly their influence on the electronic properties under study) has not been assessed quantitatively. For this reason, we have selected compound with **VI** as a test case study, since the anion in this compound features the most simplified structure along the series (thus one may expect for complex with **VI** the largest deviations due to structural simplifications, i.e. i.e. 12 methyl groups replaced by 12 hydrogen atoms).

We optimized the real structure of complex with **VI**, together with the following stationary points along the reaction path: RC, TSI, INT, PC. This allows us to evaluate not only the properties of the complex but also the first activation barrier (which is key for understanding the interaction between the complex and carbon dioxide), the stabilization of the intermediate and the thermodynamics of the overall process.

A first insight on the influence of such modifications can be obtained by analysing the optimized geometries of the real complex with **VI** (**VI real**) as well as the corresponding stationary points (see Figures S18-S19). It is immediately clear that the applied simplifications slightly affect the optimized structures, with the main geometrical parameters involved in the interaction with carbon dioxide only marginally differing from those of the simplified complex. The most remarkable difference can be found for RC, where, due to the steric hindrance of the 12 Me groups, the incoming CO_2 is oriented at about 90º with respect to the Au-Al bond (compare Figure S15 and S18). As for the reaction mechanism, a comparison between the two reaction profiles (for complexes with **VI** and **VI real**) shows that the TSI, INT and PC are destabilized with respect to those of complex with **VI**, whereas RC is stabilized (see Figure S19), consistently with the ASM results on complex with **VI real**, which indicate a larger distortion of the complex mainly due to the larger steric hindrance of the real aluminyl ligand (see below).

Indeed, we also assessed the effects of such simplifications on the ASM, EDA and CD-NOCV calculations by comparing the findings for complexes with **VI** and **VI**$^{\mathbf{real}}$ (and the corresponding stationary points). Concerning the ASM analysis, the results (see Tables S14-S15 and Figure S20), highlight only marginal differences between the simplified and the real system. In detail, we observe an increase of the electronic energy activation barrier for the real complex (1.4 vs. 4.9 kcal/mol) and a destabilization of INT (-16.6 vs. -10.4 kcal/mol). Upon inspection of the ASM terms, we find that the distortion component is increased for complex with **VI**$^{\mathbf{real}}$ mainly due to an increased distortion associated to the complex (0.6 vs 4.0 kcal/mol at TS1 and 11.4 vs 17.5 kcal/mol at INT), which could be expected in the case of complex with **VI**$^{\mathbf{real}}$ due to the increased steric hindrance of the aluminyl. Importantly, the interaction stabilization terms remain practically unaltered upon ligand simplification, which indicate that this component (which is the essential one for the purpose of the electronic structure analysis) should not be affected by the structural modelling introduced.

We also quantitatively assessed this finding by carrying out EDA and CD-NOCV calculations for the complex with **VI**$^{\mathbf{real}}$ and the corresponding TSI and INT structures. In all the cases, the results confirm quantitatively that the electronic properties of complex with **VI**$^{\mathbf{real}}$ are negligibly affected by such simplifications. In particular, results of the EDA and ETS-NOCV analysis at TS1 (Table S16) reveal that dispersion is the only term that differs (as could be expected) for more than 1 kcal/mol upon simplification (-4.6 vs. -6.5 kcal/mol). All the other EDA and ETS-NOCV terms are practically left unchanged. The electronic structure analysis of complex with **VI**$^{\mathbf{real}}$ leads to analogous conclusions, as it can be inferred by the EDA results (Table S17), CD-NOCV calculated stabilization energies and charge transfer values (Table S18) and clearly by the CD-NOCV curves, which practically overlap for the simplified and real complexes with **VI** (Figure S21).

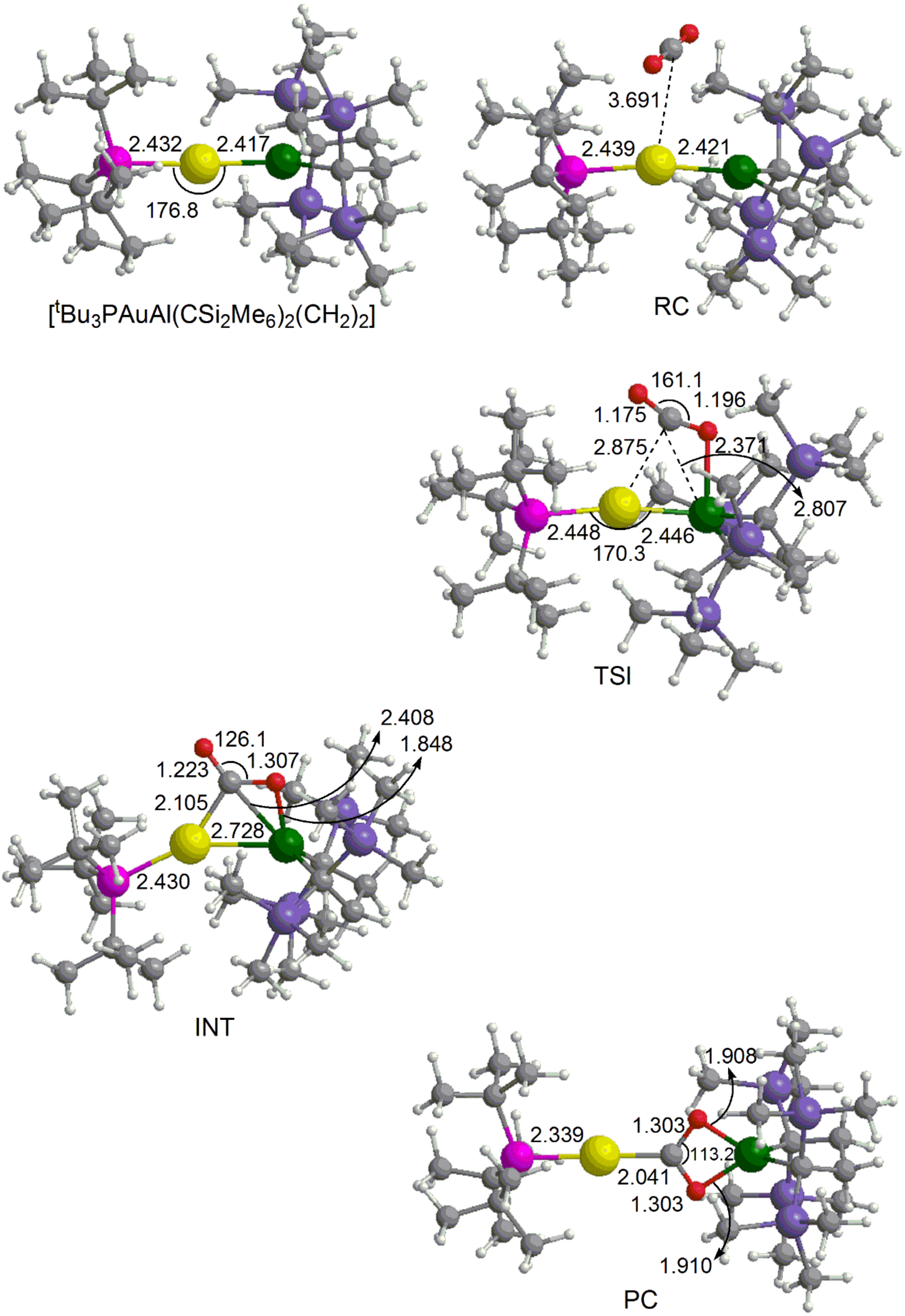

Figure S18. Optimized structures of [^{t}Bu$_3$PAuAl{(C(SiM$_3$)$_2$CH$_2$)$_2$}] (**VI real**), RC, TSI, INT and PC complexes. Main geometrical parameters are reported (bond in Å, angles in degree).

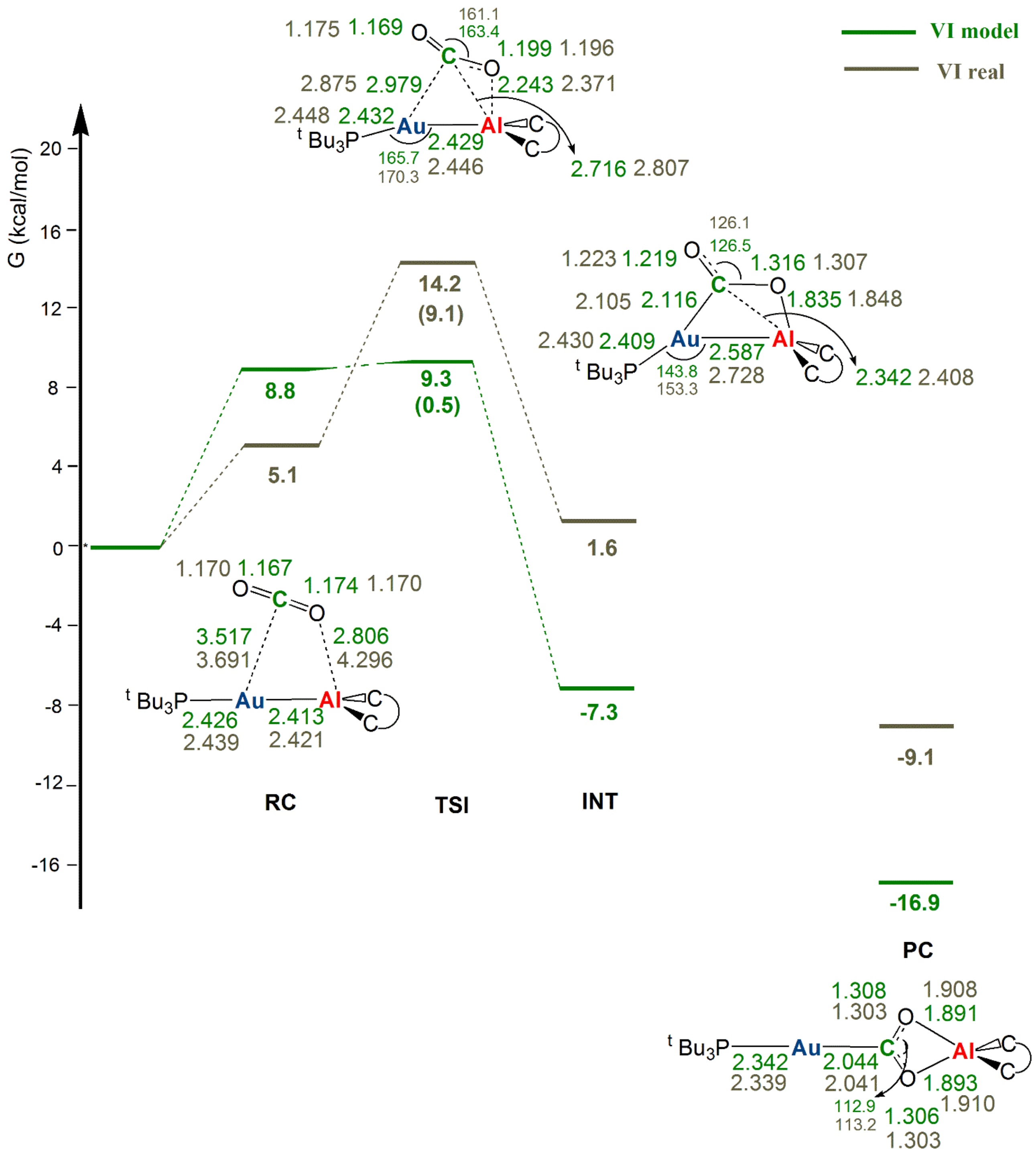

Figure S19. Free energy reaction profile for the first step of the CO_2 insertion into the Au-Al bond in the [$^tBu_3PAuAl\{C(SiH_3)_2CH_2\}_2$] (**VI** model, green lines) and [$^tBu_3PAuAl\{C(SiMe_3)_2CH_2\}_2$] (**VI real**, grey lines) complexes. PC stationary point is also reported. ΔG values refer to the energy of the separated reactants taken as zero. Activation free energy barriers are reported in parentheses. Sketches of RC, TSI, INT and PC structures are depicted with main geometrical parameters for **VI** model (in green) and for **VI** real (in grey) for comparison (bond in Å, angles in degree).

	**VI**	**VI^{real}**
**$\Delta E^{\#}$**	1.4	4.9
**ΔE_{INT}^{RC}**	-5.2	-4.8
**ΔE_{INT}^{TS}**	-9.4	-10.1
**$\Delta\Delta E_{INT}$**	-4.2	-5.3
**ΔE_{DIST}^{CO2}**	5.0	6.2
**ΔE_{DIST}^{comp}**	0.6	4.0
**$\Delta\Delta E_{DIST}$**	5.6	10.2

Table S14. Effect of the geometrical simplification of anion **VI** on the Activation Strain Model (ASM) analysis of the [CO_2]-[$^tBu_3PAuX$] (X=**VI**, **VI^{real}**) for the electronic energy activation barrier involving TSI. All energies are expressed in kcal/mol.

	**VI**	**VI^{real}**
**ΔE**	-16.6	-10.4
**ΔE_{INT}^{RC}**	-5.2	-4.8
**ΔE_{INT}^{INT}**	-93.1	-92.3
**$\Delta\Delta E_{INT}$**	-87.9	-87.5
**ΔE_{DIST}^{CO2}**	59.9	59.6
**ΔE_{DIST}^{comp}**	11.4	17.5
**$\Delta\Delta E_{DIST}$**	71.3	77.1

Table S15. Effect of the geometrical simplification of anion **VI** on the Activation Strain Model (ASM) analysis of the [CO_2]-[$^tBu_3PAuX$] (X=**VI**, **VI^{real}**) for the INT stabilization. All energies are expressed in kcal/mol.

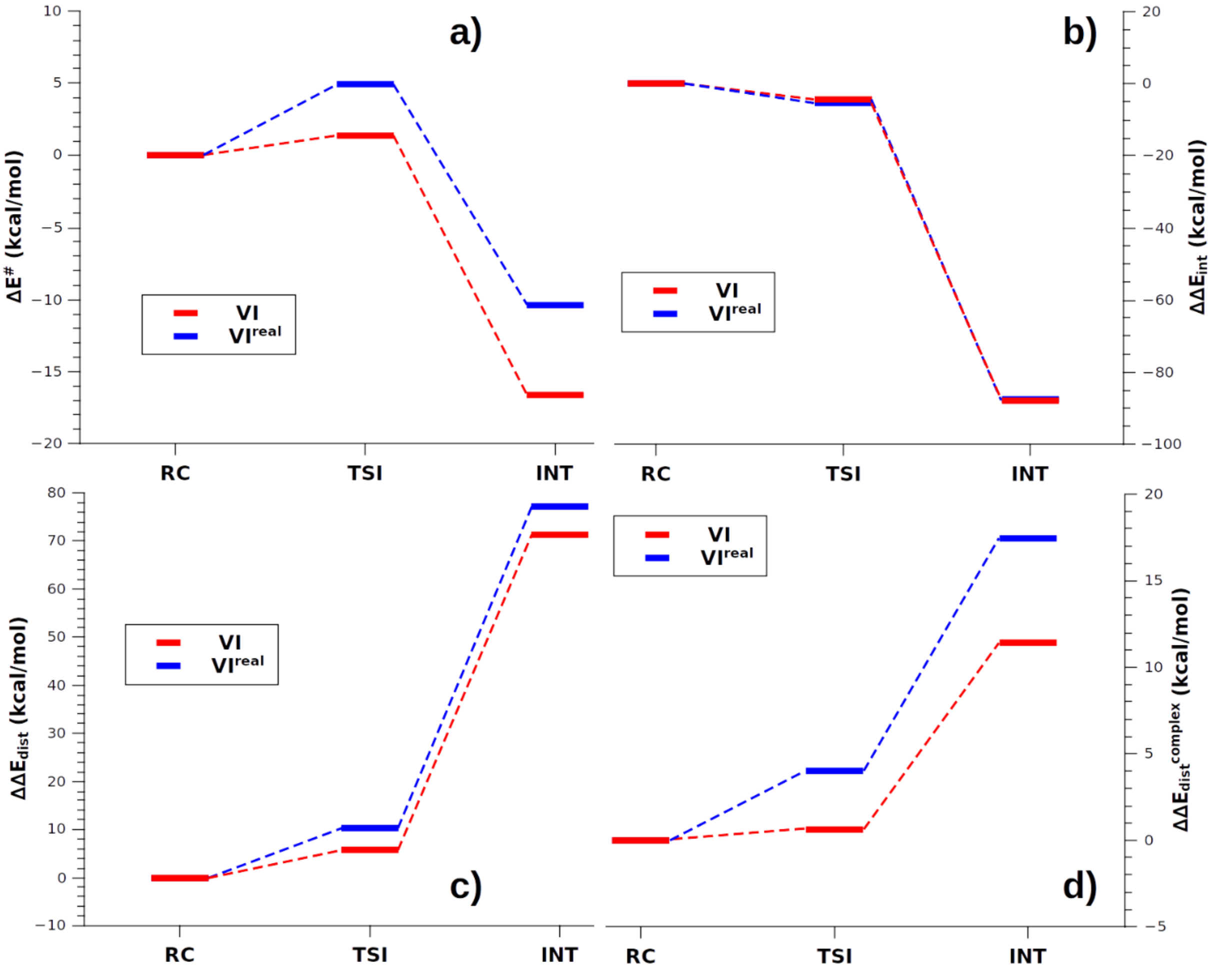

Figure S20. *a)* ASM diagrams for the electronic energy variation (*ΔE*) along the reaction path connecting RC, TSI and INT structures for complexes with **VI**/**VI**^{real}. *b)* ASM diagrams for the variation of the interaction energy stabilization ($\Delta\Delta E_{int}$) along the reaction path connecting RC, TSI and INT structures for complexes with **VI**/**VI**^{real}. *c)* ASM diagrams for the overall variation of the distortion energy penalty ($\Delta\Delta E_{dist}$) along the reaction path connecting RC, TSI and INT structures for complexes with **VI**/**VI**^{real}. *d)* ASM diagrams for the penalty due to the distortion of the complex ($\Delta E_{dist}^{complex}$) along the reaction path connecting RC, TSI and INT structures for complexes with **VI**/**VI**^{real}.

	**VI**	**VI^{real}**
**ΔE_{Pauli}**	60.5	59.1
**ΔE_{elst}**	-39.8	-38.1
**ΔE_{Steric}**	20.7	21.0
**ΔE_{oi}**	-27.3	-25.9
**ΔE_{oi}^1**	-15.9	-14.9
**ΔE_{oi}^2**	-5.9	-5.3
**ΔE_{disp}**	-4.6	-6.5
**ΔE**	-11.1	-11.4

Table S16. Effect of the geometrical simplification of anion **VI** on the Energy Decomposition Analysis (EDA) of the [CO_2]-[^{t}Bu$_3$AuX] (X=**VI**, **VI^{real}**) interaction occurring at TSI. All energies are expressed in kcal/mol.

	**VI**	**VI^{real}**
**ΔE_{Pauli}**	155.71	181.00
**ΔE_{Elst}**	-160.60	-183.14
**ΔE_{Steric}**	-4.89	-2.14
**ΔE_{oi}**	-72.80	-72.33
**ΔE_{disp}**	-5.61	-10.39
**ΔE**	-83.30	-84.86

Table S17. Effect of the geometrical simplification of anion **VI** on the Energy Decomposition Analysis (EDA) for the interaction between neutral doublet [^{t}Bu$_3$PAu]· and [X]· (X=**VI**, **VI^{real}**) for complexes [^{t}Bu$_3$PAuX]. Energies are reported in kcal/mol.

Complex	VI	VI^{real}
$CT^{1\alpha}$	-0.298	-0.284
$\Delta E_{oi}^{1\alpha}$	-34.6	-32.3
$CT^{1\beta}$	0.307	0.306
$\Delta E_{oi}^{1\beta}$	-23.8	-24.9
CT^{1}	0.009	0.022
ΔE_{oi}^{2}	-4.7	-4.4
CT^{2}	-0.032	-0.031
ΔE_{oi}^{3}	-3.5	-3.5
CT^{3}	-0.020	-0.016
ΔE_{oi}	-72.8	-72.3
ΔE	-83.3	-84.8

Table S18. Effect of the geometrical simplification of anion **VI** on the orbital interaction energies (ΔE_{oi}^{k}) and charge transfer (CT^{k}) associated to the first three NOCV deformation densities for the interaction between neutral doublet [$^{t}Bu_3PAu$]· and [X]· (X=**VI**, **VI^{real}**) for complexes [$^{t}Bu_3PAuX$]. The overall ΔE_{oi} and ΔE values from the EDA are also reported. Energies are reported in kcal/mol.

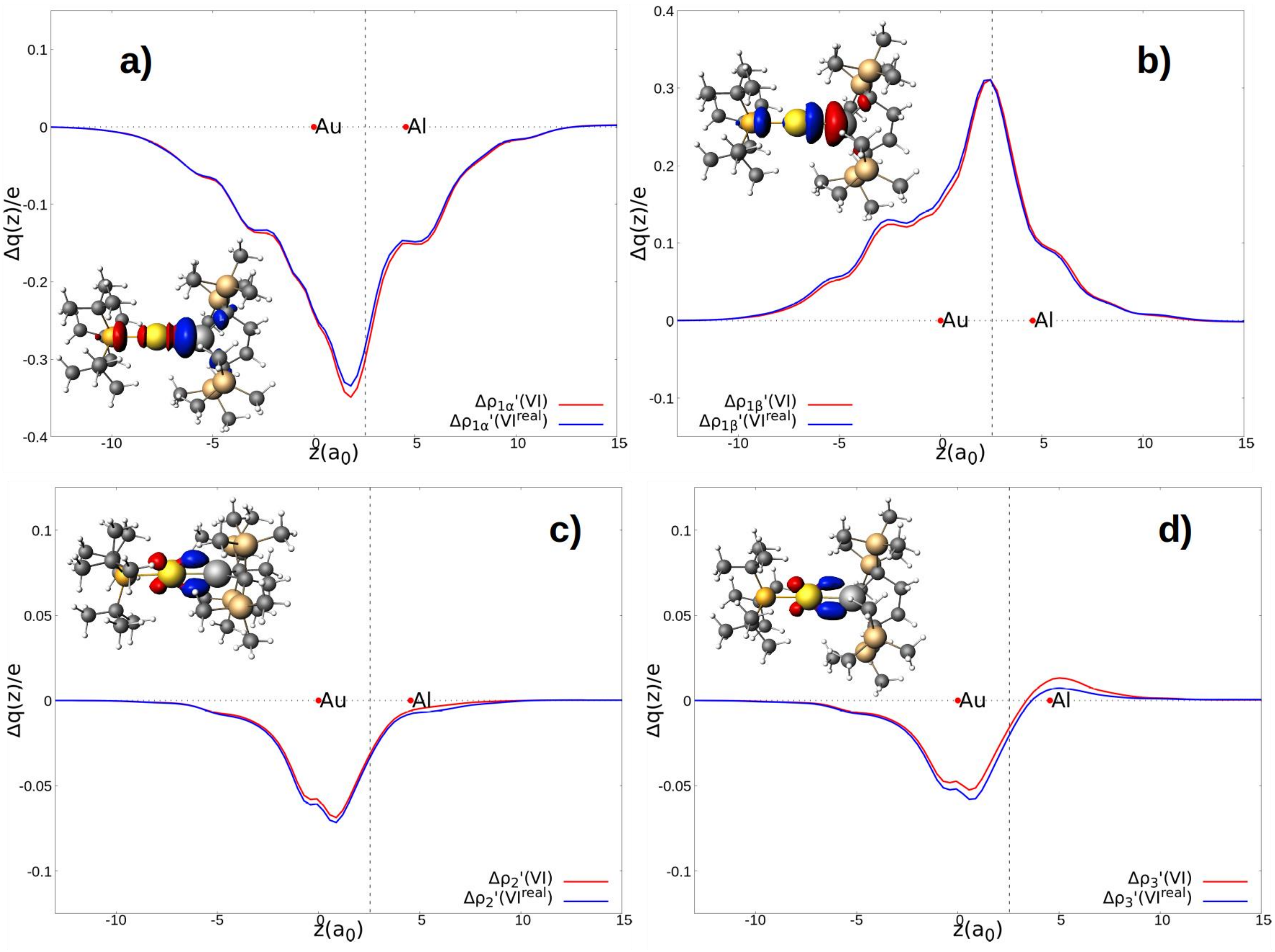

Figure S21. Charge Displacement (CD-NOCV) curves and related isosurfaces (isovalue 3 me/a_0^3) associated to the $\Delta\rho_{1\alpha}$'(a), $\Delta\rho_{1\beta}$'(b), $\Delta\rho_2$'(c) and $\Delta\rho_3$'(d) NOCV deformation densities for the interaction between doublet [^{t}Bu$_3$PAu]· and [X]· (X=Al({C(SiH$_3$)$_2$CH$_2$}$_2$), Al({C(Si(Me$_3$)$_2$CH$_2$}$_2$)) fragments for complexes with **VI** and **VI^{real}**, respectively. Red dots indicate the average position of the nuclei along the z axis. Positive (negative) values of the curve indicate right-to-left (left-to-right) charge transfer. The black dashed line indicates the average position of the isodensity boundary.

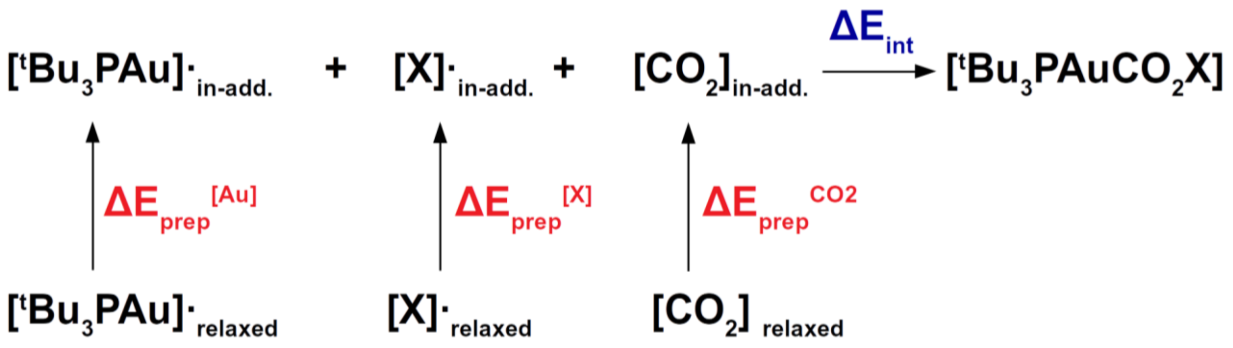

Scheme S1. Scheme for the formation of the insertion products [^{t}Bu$_3$PAuCO$_2$X] starting from relaxed radical [X]· and [^{t}Bu$_3$PAu]· fragments and CO_2.

	ΔE_{int} (kcal/mol)	ΔE_{prep}^{CO2} (kcal/mol)	$\Delta E_{prep}^{[X]}$ (kcal/mol)	$\Delta E_{prep}^{[Au]}$ (kcal/mol)	ΔE_{prep} (kcal/mol)	ΔE (kcal/mol)
**I**	-200.7	94.4	0.1	0.3	94.8	-106.0
**II**	-205.8	95.3	0.7	0.3	96.3	-109.5
**III**	-206.2	94.9	0.6	0.3	95.8	-110.4
**IV**	-208.8	96.8	0.3	0.3	97.4	-111.4
**V**	-202.3	95.5	0.1	0.2	95.9	-106.5
**VI**	-205.3	95.6	0.8	0.2	96.6	-108.8

Table S19. Interaction energy (ΔE_{int}) and preparation energy of the [P^{t}Bu$_3$Au] ($\Delta E_{prep}^{[Au]}$), aluminyl ($\Delta E_{prep}^{[X]}$) and CO_2 (ΔE_{prep}^{CO2}) fragments considered for the formation of PCs [^{t}Bu$_3$PAuCO$_2$X] (X = **I**, **II**, **III**, **IV**, **V**, **VI**). The overall preparation (ΔE_{prep}) and formation (ΔE) energies are also reported. All energies are expressed in kcal/mol.

	Gas phase	COSMO solvation
**ΔE_{Pauli}**	60.5	59.9
**ΔE_{elst}**	-39.8	-40.2
**ΔE_{Steric}**	20.7	19.8
**ΔE_{oi}**	-27.3	-26.5
**ΔE_{oi}^1**	-15.9	-15.4
**ΔE_{oi}^2**	-5.9	-5.9
**ΔE_{disp}**	-4.6	-4.6
**ΔE_{solv}**	/	-2.1
**ΔE**	-11.1	-13.4

Table S20. Effect of the inclusion of implicit solvation (COSMO, toluene solvent) on the EDA and ETS-NOCV analysis of the $[CO_2]$-$[^tBu_3AuX]$ (X=Al($\{C(SiH_3)_2CH_2\}_2$) interaction for the TSI involving complex with **VI**. All energies are expressed in kcal/mol.

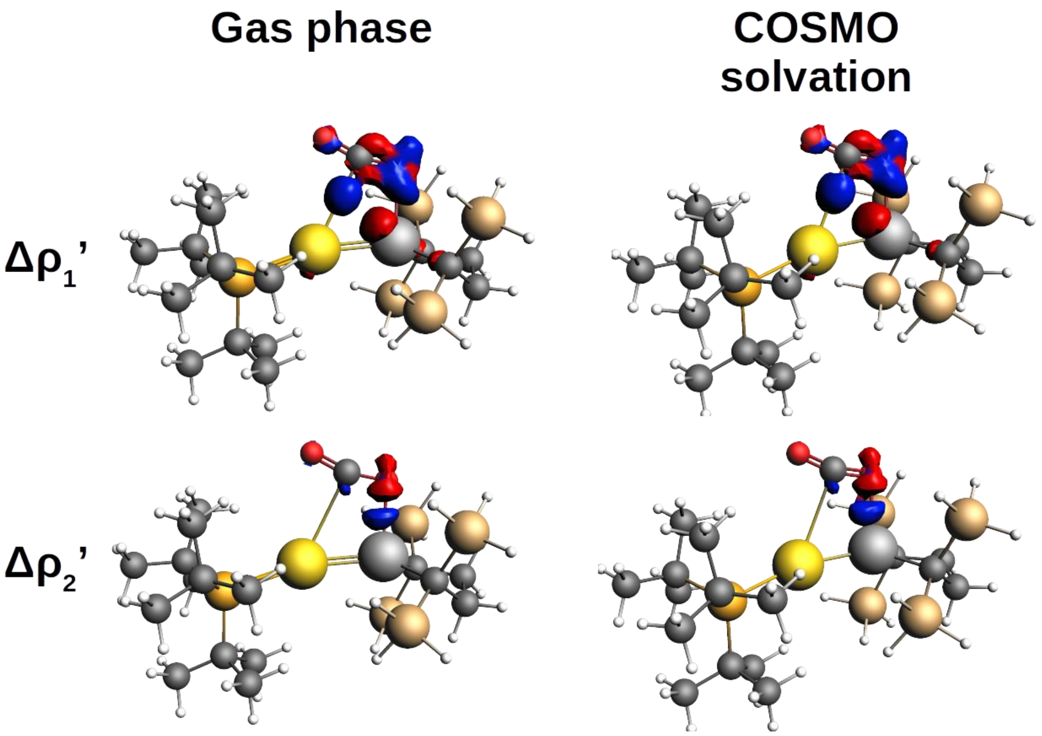

Figure S22. Isodensity surfaces of the *Δρ₁'* and *Δρ₂'* NOCV deformation densities for the interaction between $[^tBu_3PAuAl(\{C(SiH_3)_2CH_2\}_2)]$ and $[CO_2]$ fragments calculated without solvent (left) and including solvation effects (toluene) with COSMO (right). Blue regions indicate electron density accumulation areas, whereas red regions indicate depletion areas. Isodensity value is 2 me/a_0^3.

PBE vs. PBE0 functional results

The choice of using the PBE functional for all calculations is based on the combined experimental/theoretical work where complex with **I** was originally reported.[17] In that framework, DFT calculations using PBE and PBE0 were benchmarked against the reference DLPNO-CCSD(T) approach and the authors concluded that both PBE and PBE0 were accurate in reproducing the magnitude and the trends in bond energies and thus they could be considered reliable. We also mention that we already discussed in a previous work how the PBE-D3(BJ)/TZ2P optimized geometry for compound with **I** is in good agreement with the experimental one.[16]
However, it is clear that a proper assessment/benchmark of the actual performance of various XC functionals in this context would be highly desirable. Despite an extensive benchmark would be far from the scope of this work, we have tested the effect of using a hybrid functional for the energy reaction profile and electronic structure calculations we present. We selected the PBE0 functional since it belongs to a different familiy of density functionals (hybrid) from the one we used (GGA) and because of its good performance in this context when benchmarked against DLPNO-CCSD(T). In particular, we used the PBE0 functional for calculating the energies of compounds with **I-VI** and of the corresponding most significant stationary points along the reaction paths (RC, TSI, PC), which should give an idea of the effect on the functional on both the kinetics and thermodynamics of the reaction. Furthermore, we tested the effect of the functional on the EDA, ETS-NOCV and CD-NOCV analyses using compound with **VI** as a test case.
Our comparative analysis reveals that, overall, the effect of the functional is not remarkable for the discussion we present here: the results are unbiased with respect to the use of PBE/PBE0 functionals.
Concerning the reaction mechanism, the electronic energy profiles (see Table S21 and Figure S23) obtained with PBE0 are in agreement with the PBE ones: in most cases, the energy of RC, TSI and the relative electronic activation barrier differ by 1 kcal/mol ca. In the worst case scenario, represented by compound with **VI**, we observe larger deviations for RC and TS1 (deviation of -5.4 kcal/mol on both RC and TSI) but overall the electronic barrier is unchanged upon using PBE0, thus highlighting that the relative stationary points are only shifted at lower energies. Concerning the stability of PC, we find that, PBE0 yields more stable PC in all cases (on average there is a deviation of 7 kcal/mol ca); however, the shift is found to be practically systematic, as it can be clearly inferred from the profiles reported in Figure S23. Overall, we can conclude that both the kinetics and thermodynamics trends of the reactions under study are unbiased upon choice of a different density functional.
Concerning the impact of the functional on the electronic structure calculations, the effect of using PBE0 functional is even less remarkable. The analysis of the Au-Al bond in complex **VI** leads to practically identical results for PBE and PBE0, as demonstrated by the small variations reported for the EDA terms (Table S22), CD-NOCV terms (Table S23) as well as the CD-NOCV curves (which practically overlap, see Figure S24) and related deformation densities.

	Functional	Reactants	RC	TSI	ΔE[#]	PC
**I**	**PBE**	0.0	-5.1	3.7	8.8	-27.2
	**PBE0**	0.0	-5.1	5.3	10.4	-34.8
**II**	**PBE**	0.0	-5.7	-0.4	5.3	-26.9
	**PBE0**	0.0	-6.0	0.1	6.1	-34.5
**III**	**PBE**	0.0	-4.2	0.4	4.6	-29.6
	**PBE0**	0.0	-4.2	1.0	5.2	-36.9
**IV**	**PBE**	0.0	-4.7	1.2	5.9	-25.8
	**PBE0**	0.0	-5.0	2.0	7.0	-32.7
**V**	**PBE**	0.0	-3.9	0.9	4.8	-25.7
	**PBE0**	0.0	-4.1	1.6	5.7	-33.0
**VI**	**PBE**	0.0	-4.5	-2.3	2.2	-29.7
	**PBE0**	0.0	-9.9	-7.7	2.2	-36.0

Table S21. Relative energies for the formation of RC, TSI and PC structures from separated reactants (CO_2 and complexes with **I-VI**, taken as reference) using a GGA (PBE) and a hybrid (PBE0) exchange-correlation functional. All energies are in kcal/mol.

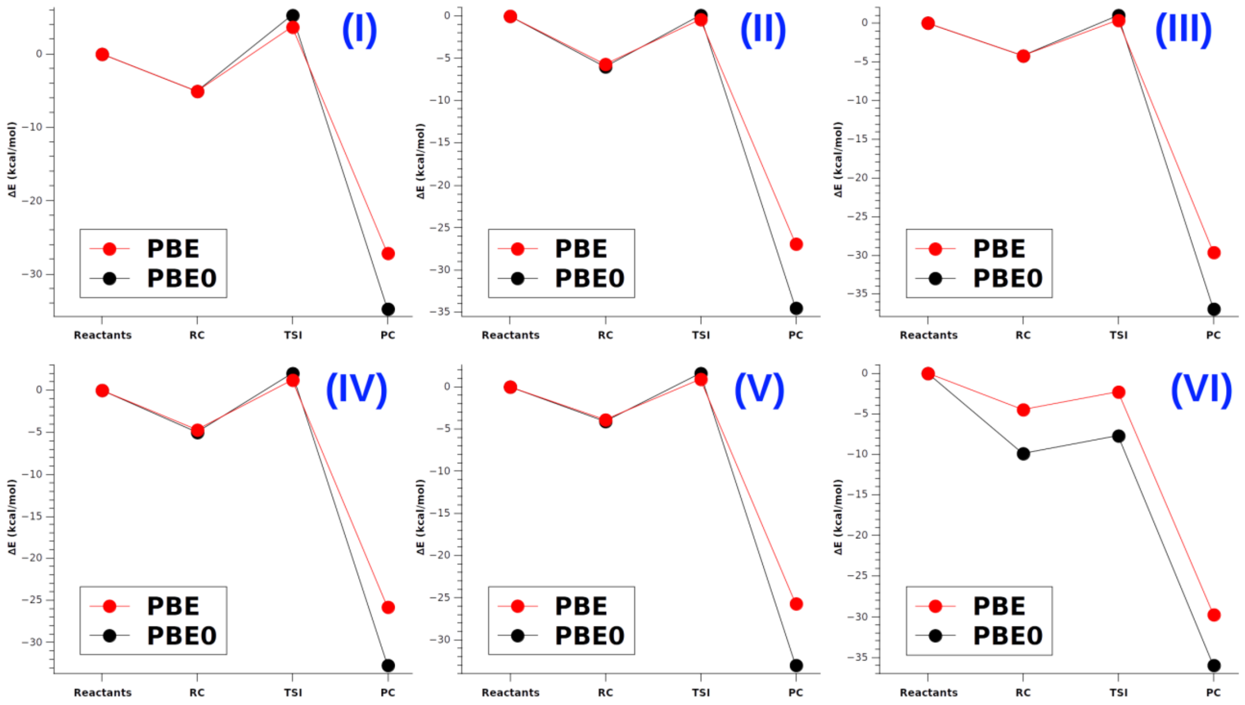

Figure S23. Electronic energy profiles for the formation of RC, TSI and PC structures from separated reactants (CO_2 and complexes with **I-VI**, taken as reference) using a PBE (red) and an PBE0 (black) exchange-correlation functional. All energies are in kcal/mol.

	PBE	PBE0
**ΔE_{Pauli}**	155.71	168.88
**ΔE_{Elst}**	-160.60	-178.74
**ΔE_{Steric}**	-4.89	-9.86
**ΔE_{oi}**	-72.80	-69.68
**ΔE_{disp}**	-5.61	-5.61
**ΔE**	-83.30	-85.15

Table S22. Effect of the exchange-correlation functional on the orbital interaction energies (ΔE_{oi}^{k}) and charge transfer (CT^{k}) associated to the first three NOCV deformation densities for the interaction between neutral doublet [$^{t}Bu_3PAu$]· and [X]· (X=**VI**) for complexes [$^{t}Bu_3PAuX$] using the GGA PBE and hybrid PBE0 functionals. The overall ΔE_{oi} and ΔE values from the EDA are also reported. Energies are reported in kcal/mol.

**Complex**	**PBE**	**PBE0**
$CT^{1\alpha}$	-0.298	-0.276
$\Delta E_{oi}^{1\alpha}$	-34.6	-34.2
$CT^{1\beta}$	0.307	0.281
$\Delta E_{oi}^{1\beta}$	-23.8	-21.1
CT^{1}	0.009	0.005
ΔE_{oi}^{2}	-4.7	-4.5
CT^{2}	-0.032	-0.028
ΔE_{oi}^{3}	-3.5	-4.0
CT^{3}	-0.020	-0.019

Table S23. Effect of the exchange-correlation functional on the orbital interaction energies (ΔE_{oi}^{k}) and charge transfer (CT^{k}) associated to the first three NOCV deformation densities for the interaction between neutral doublet [$^{t}Bu_3PAu$]· and [X]· (X=**VI**) for complexes [$^{t}Bu_3PAuX$] using the GGA PBE and hybrid PBE0 functionals. Energies are reported in kcal/mol.

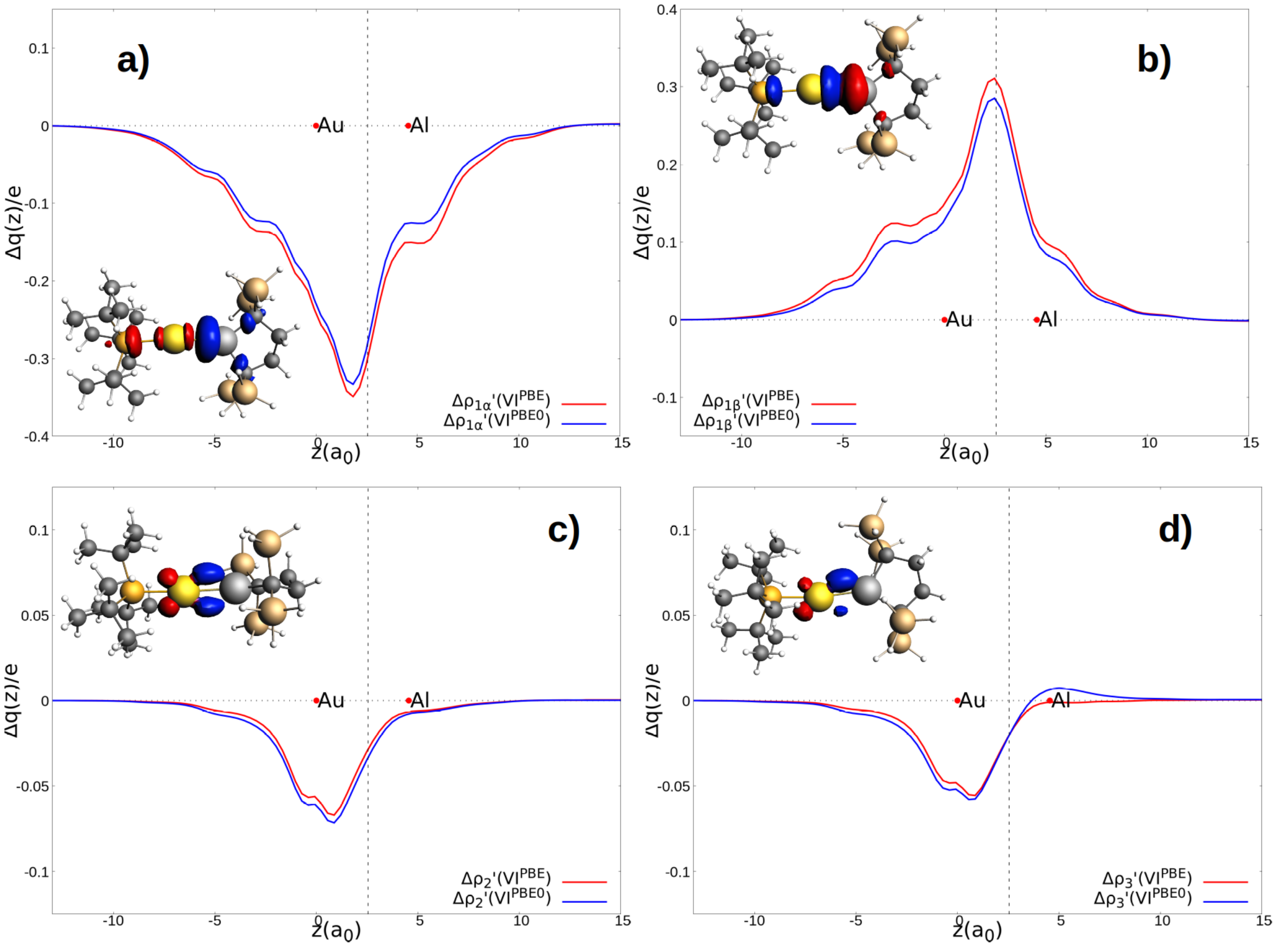

Figure S24. Charge Displacement (CD-NOCV) curves and related isosurfaces (isovalue 3 me/a_0^3) associated to the *Δρ$_{1α}$'*(a), *Δρ$_{1β}$'*(b), *Δρ$_2$'*(c) and *Δρ$_3$'*(d) NOCV deformation densities for the interaction between doublet [^{t}Bu$_3$PAu]· and [X]· (X=Al({C(SiH$_3$)$_2$CH$_2$}$_2$)) fragments for complex with **VI** calculated using PBE and PBE0 density functionals, respectively. Red dots indicate the average position of the nuclei along the z axis. Positive (negative) values of the curve indicate right-to-left (left-to-right) charge transfer. The black dashed line indicates the average position of the isodensity boundary.

References

1 M. Mitoraj and A. Michalak, *J. Mol. Model.*, 2007, **13**, 347–355.

2 A. Michalak, M. Mitoraj and T. Ziegler, *J. Phys. Chem. A*, 2008, **112**, 1933–1939.

3 R. F. Nalewajski and J. Mrozek, *Int. J. Quantum Chem.*, 1994, **51**, 187–200.

4 R. F. Nalewajski, J. Mrozek and A. Michalak, *Int. J. Quantum Chem.*, 1997, **61**, 589–601.

5 T. Lu and F. Chen, *J. Phys. Chem. A*, 2013, **117**, 3100–3108.

6 G. Bistoni, S. Rampino, F. Tarantelli and L. Belpassi, *J. Chem. Phys.*, 2015, **142**, 084112.

7 L. Belpassi, I. Infante, F. Tarantelli and L. Visscher, *J. Am. Chem. Soc.*, 2008, **130**, 1048–1060.

8 N. Salvi, L. Belpassi and F. Tarantelli, *Chem. - A Eur. J.*, 2010, **16**, 7231–7240.

9 G. Bistoni, L. Belpassi and F. Tarantelli, *J. Chem. Theory Comput.*, 2016, **12**, 1236–1244.

10 T. Ziegler and A. Rauk, *Theor. Chim. Acta*, 1977, **46**, 1–10.

11 K. Morokuma, *J. Chem. Phys.*, 1971, **55**, 1236–1244.

12 M. P. Mitoraj, A. Michalak and T. Ziegler, *J. Chem. Theory Comput.*, 2009, **5**, 962–975.

13 I. Ferná and F. M. Bickelhaupt, *Chem. Soc. Rev*, 2014, **43**, 4953.

14 F. M. Bickelhaupt and K. N. Houk, *Angew. Chemie - Int. Ed.*, 2017, **56**, 10070–10086.

15 P. Vermeeren, S. C. C. van der Lubbe, C. Fonseca Guerra, F. M. Bickelhaupt and T. A. Hamlin, *Nat. Protoc.*, 2020, **15**, 649–667.

16 D. Sorbelli, L. Belpassi and P. Belanzoni, *J. Am. Chem. Soc.*, 2021, **143**, 14433–14437.

17 J. Hicks, A. Mansikkamäki, P. Vasko, J. M. Goicoechea and S. Aldridge, *Nat. Chem.*, 2019, **11**, 237–241.

xyz geometries

90

Complex [$^{t}Bu_3PAu$-**III**]

Al	1.507428	0.177348	0.239264
Au	-0.906347	0.130312	0.171744
C	3.412071	3.482829	1.448684
C	2.397251	2.812577	0.735162
C	1.104530	3.377793	0.760164
C	0.840754	4.547127	1.470083
C	1.860995	5.204751	2.163113
C	3.148925	4.664174	2.140798
N	2.652873	1.616564	0.032571
Si	3.976519	1.514975	-1.128214
C	3.351391	0.492411	-2.589641
N	2.520492	-1.364160	0.344822
Si	4.150921	-1.421901	1.015749
C	4.183595	-0.341146	2.561541
P	-3.304206	-0.049828	-0.069675
C	1.910545	-2.543225	-0.148734
C	0.711170	-3.022561	0.414303
C	0.073260	-4.151444	-0.097645
C	0.622650	-4.847570	-1.176545
C	1.821445	-4.396492	-1.734909
C	2.452878	-3.258054	-1.234231
C	5.545311	0.708241	-0.433944
C	5.480833	-0.813049	-0.193957
C	4.409924	3.242065	-1.748074
C	4.557045	-3.200510	1.490010
H	-0.170681	4.958087	1.468442
H	1.656162	6.125953	2.709081
H	3.958988	5.161974	2.676564
H	3.503748	3.775901	-2.068040
H	5.085018	3.162242	-2.612805
H	4.907087	3.850974	-0.982069
H	3.002273	-0.502843	-2.280479
H	4.156310	0.347568	-3.325996
H	2.520310	1.009374	-3.090801
H	6.362893	0.951220	-1.136102
H	5.798058	1.228790	0.504907
H	6.449872	-1.179862	0.188844
H	5.322019	-1.340494	-1.149601
H	3.864838	0.686566	2.339885
H	5.200501	-0.296966	2.979612
H	3.514466	-0.751612	3.331255
H	3.770139	-3.622209	2.131339
H	5.506278	-3.230466	2.044573
H	4.655366	-3.848135	0.608481
H	-0.855474	-4.496368	0.360310
H	0.126776	-5.733628	-1.573918
H	2.266538	-4.930770	-2.576056
H	0.308837	2.879126	0.203488
H	4.418168	3.060585	1.458835
H	0.293273	-2.491184	1.269524
H	3.378544	-2.903216	-1.690448
C	-4.025842	-1.131967	1.339996
C	-3.658537	-0.877863	-1.763812
C	-4.100413	1.693611	-0.017553
C	-3.758330	2.424047	-1.326922
C	-5.620401	1.714551	0.187084
C	-3.408624	2.496751	1.103070
C	-2.692549	-0.271015	-2.802171
C	-3.282208	-2.366361	-1.667161
C	-5.103120	-0.749592	-2.263676
C	-5.438971	-1.674049	1.091213
C	-3.053354	-2.303495	1.582678
C	-4.021473	-0.309899	2.639690
H	-4.054667	3.477835	-1.214741
H	-2.679626	2.400233	-1.531747
H	-4.295368	2.022565	-2.192258
H	-3.747910	3.541831	1.035851
H	-3.642477	2.134915	2.107035
H	-2.316262	2.481735	0.979847
H	-5.966035	2.759570	0.149760
H	-6.154565	1.160468	-0.592854
H	-5.915906	1.309711	1.161523
H	-4.262139	-0.989226	3.471184
H	-3.032426	0.123784	2.839225
H	-4.768656	0.490192	2.642335
H	-5.764298	-2.228383	1.985497
H	-6.168455	-0.876193	0.911645
H	-5.474362	-2.370920	0.246491
H	-3.390444	-2.851523	2.475740
H	-3.009321	-3.011334	0.752132
H	-2.035775	-1.933596	1.769216
H	-2.785830	-0.848760	-3.734505
H	-2.904705	0.774685	-3.036073
H	-1.650856	-0.342647	-2.457019
H	-3.315741	-2.788980	-2.682627
H	-2.261772	-2.503468	-1.283963
H	-3.978332	-2.943779	-1.050088
H	-5.197277	-1.304384	-3.210314
H	-5.830340	-1.169219	-1.559576
H	-5.381705	0.290357	-2.468368

93

RC (III)

C	1.069837	-3.308932	-0.202742
C	1.914195	-2.737878	0.769727
C	1.852089	-3.265749	2.074826
C	0.993391	-4.317901	2.387884
C	0.159763	-4.869095	1.411442
C	0.203721	-4.354490	0.114736
N	2.741589	-1.635640	0.438420
SI	4.446287	-1.660690	0.895397
C	5.018565	-3.440686	1.150926
AL	1.734526	-0.160263	-0.046033
AU	-0.681642	-0.062690	-0.039119
P	-3.075565	0.220596	-0.206936
C	-3.662844	1.566559	1.027181
C	-2.895644	1.352903	2.347908
N	2.672048	1.344408	-0.600525
SI	4.142692	1.401086	-1.568609
C	4.681130	3.188239	-1.849223
C	1.840200	2.488060	-0.512257
C	1.345409	2.901094	0.741416
C	0.478304	3.987322	0.856170
C	0.084636	4.700997	-0.277202
C	0.561339	4.301600	-1.529052
C	1.416920	3.208758	-1.646658
C	3.884919	0.525493	-3.223905
C	5.578648	0.563689	-0.671587
C	5.531831	-0.961796	-0.487054
C	4.740040	-0.641941	2.458685
C	-3.973885	-1.433249	0.164747
C	-3.913056	-1.694430	1.679409
C	-3.468778	0.781987	-1.998799
C	-4.872213	1.362602	-2.212585
C	-5.432414	-1.508151	-0.304712
C	-3.154986	-2.568474	-0.482326
C	-2.405002	1.817945	-2.416268
C	-3.272385	-0.421046	-2.936415
C	-3.219437	2.944811	0.510115
C	-5.173307	1.593626	1.292819
H	0.248808	4.836258	-2.427659
H	-0.591690	5.551527	-0.188764

H	0.109642	4.277787	1.841299
H	4.017910	3.738676	-2.527480
H	5.693147	3.194767	-2.280269
H	4.713818	3.733578	-0.895043
H	3.548878	-0.510462	-3.067534
H	4.815635	0.493353	-3.809845
H	3.119126	1.036632	-3.824717
H	6.464413	0.839785	-1.272181
H	5.713262	1.070777	0.298276
H	6.542072	-1.335854	-0.240690
H	5.256225	-1.466146	-1.428196
H	4.362990	0.382223	2.322538
H	5.810967	-0.582108	2.703261
H	4.213776	-1.076282	3.320245
H	4.527436	-3.933873	1.998624
H	6.103596	-3.449305	1.330697
H	4.817614	-4.039846	0.250980
H	0.965431	-4.700642	3.409457
H	-0.517010	-5.686762	1.660120
H	-0.440641	-4.771119	-0.660989
H	1.745450	2.882314	-2.634063
H	1.663648	2.355984	1.631984
H	2.475551	-2.824458	2.853683
H	1.110965	-2.918284	-1.220545
H	-3.392706	3.677255	1.313592
H	-2.149004	2.966157	0.270012
H	-3.790968	3.276785	-0.362887
H	-3.151779	2.176740	3.031868
H	-3.136485	0.412238	2.848652
H	-1.811272	1.379967	2.168670
H	-5.395162	2.433409	1.970039
H	-5.753851	1.745688	0.375528
H	-5.531426	0.679973	1.779833
H	-4.273414	-2.718649	1.859437
H	-2.884376	-1.633879	2.056863
H	-4.548545	-1.016499	2.258260
H	-5.852502	-2.476919	0.008917
H	-6.055124	-0.719332	0.132174
H	-5.522066	-1.454190	-1.395624
H	-3.586131	-3.528358	-0.158271
H	-3.170774	-2.548611	-1.574688
H	-2.107638	-2.537186	-0.151767
H	-2.528166	2.019626	-3.491666
H	-2.488934	2.769271	-1.886316
H	-1.388419	1.433258	-2.250922
H	-3.314971	-0.054091	-3.973103
H	-2.290555	-0.890099	-2.786499
H	-4.051790	-1.181962	-2.824776
H	-4.996759	1.595853	-3.281841
H	-5.664642	0.659724	-1.932148
H	-5.025760	2.294697	-1.657398
C	0.242371	-0.693189	3.406560
O	1.222280	-0.055492	3.336058
O	-0.737905	-1.323152	3.507774

93

TSI (III)

C	1.277413	-2.079455	-1.972412
C	1.924178	-2.335173	-0.746622
C	1.715387	-3.594050	-0.151160
C	0.909090	-4.554317	-0.759630
C	0.276700	-4.284609	-1.976351
C	0.463426	-3.038226	-2.575815
N	2.707918	-1.324155	-0.131353
SI	4.362249	-1.693656	0.344452
C	4.978950	-3.213731	-0.587246
AL	1.626124	0.116138	0.305128
AU	-0.786564	-0.110269	0.373358
P	-3.134996	-0.135704	-0.203883
C	-4.108145	1.077455	0.916454
C	-3.562948	0.927965	2.351358
N	2.268172	1.799909	-0.075536
SI	3.885748	2.099445	-0.698090
C	4.283381	3.936019	-0.528611
C	1.297034	2.827866	-0.083570
C	0.588711	3.153696	1.090172
C	-0.398004	4.138948	1.089195
C	-0.702740	4.836589	-0.081374
C	-0.007533	4.528145	-1.253566
C	0.971517	3.536619	-1.256655
C	4.059003	1.574187	-2.504897
C	5.153432	1.148050	0.347424
C	5.524280	-0.274595	-0.109032
C	4.515078	-1.998147	2.204465
C	-3.827018	-1.911411	-0.000574
C	-3.975543	-2.204408	1.501978
C	-3.275458	0.418735	-2.034253
C	-4.685620	0.819592	-2.486421
C	-5.163112	-2.176985	-0.705290
C	-2.758880	-2.895955	-0.516268
C	-2.298189	1.592599	-2.249443
C	-2.764955	-0.722238	-2.930240
C	-3.776058	2.517488	0.493522
C	-5.630040	0.888889	0.909525
H	-0.238750	5.055529	-2.180602
H	-1.472704	5.608535	-0.081285
H	-0.928839	4.366907	2.015040
H	3.589091	4.566833	-1.098450
H	5.302984	4.130196	-0.892401
H	4.231749	4.244063	0.525867
H	3.751959	0.525341	-2.625725
H	5.098545	1.668993	-2.853116
H	3.425200	2.192038	-3.156781
H	6.053793	1.787456	0.330228
H	4.813742	1.159322	1.397059
H	6.496681	-0.569748	0.325468
H	5.671500	-0.301010	-1.201077
H	4.244169	-1.103576	2.781945
H	5.548772	-2.271246	2.464162
H	3.854705	-2.815391	2.528446
H	4.485004	-4.139351	-0.265940
H	6.060030	-3.326834	-0.416842
H	4.813074	-3.099854	-1.668038
H	0.762186	-5.518929	-0.270989
H	-0.358634	-5.035407	-2.446578
H	-0.021610	-2.810068	-3.526352
H	1.485170	3.282798	-2.184894
H	0.841657	2.631228	2.013017
H	2.178282	-3.803758	0.814151
H	1.439098	-1.112907	-2.454232
H	-4.193885	3.195939	1.253099
H	-2.693529	2.692622	0.453740
H	-4.218350	2.793066	-0.469301
H	-4.024920	1.707373	2.976735
H	-3.785587	-0.040560	2.805528
H	-2.473819	1.073317	2.371099
H	-6.083078	1.667725	1.543009
H	-6.057416	0.988125	-0.094900
H	-5.934062	-0.080840	1.319282
H	-4.198598	-3.275761	1.617161
H	-3.044121	-1.997195	2.046075
H	-4.793360	-1.646462	1.969577
H	-5.480803	-3.207237	-0.480152
H	-5.958276	-1.502908	-0.367375
H	-5.080097	-2.094030	-1.794983
H	-3.077923	-3.916634	-0.254928
H	-2.617442	-2.860100	-1.598231
H	-1.782441	-2.712381	-0.046612

H	-2.273711	1.824352	-3.325463
H	-2.578735	2.504595	-1.718141
H	-1.280301	1.324593	-1.932430
H	-2.666318	-0.329935	-3.953771
H	-1.776117	-1.075656	-2.608836
H	-3.452736	-1.573362	-2.969285
H	-4.652504	1.066919	-3.559121
H	-5.413093	0.010437	-2.354758
H	-5.056968	1.705918	-1.960178
C	0.738100	-0.370738	2.837008
O	1.852802	0.023964	2.636941
O	-0.207452	-0.786553	3.396329

93

INT (III)

C	-2.256125	-2.902075	1.552007
C	-1.913197	-2.405003	0.279614
C	-0.967765	-3.128785	-0.472021
C	-0.405011	-4.305284	0.020396
C	-0.762454	-4.790696	1.280092
C	-1.692459	-4.079418	2.041690
N	-2.527680	-1.250355	-0.253556
SI	-4.273038	-1.259049	-0.505550
C	-4.884179	-3.024917	-0.716626
AL	-1.620184	0.240478	-0.788071
AU	0.965217	-0.021709	-0.922701
P	2.740196	-0.349636	0.661953
C	3.826096	1.229055	0.637533
C	3.899379	1.737627	-0.817086
N	-1.986735	1.740380	0.206505
SI	-3.198955	1.786238	1.506552
C	-3.551430	3.588304	1.949021
C	-1.137951	2.862228	0.016510
C	-0.929118	3.402207	-1.267948
C	-0.073403	4.485540	-1.463903
C	0.580428	5.081221	-0.383677
C	0.375407	4.565013	0.897529
C	-0.455623	3.463761	1.092392
C	-2.645583	0.845821	3.047282
C	-4.897852	1.115905	0.977922
C	-5.172595	-0.398183	0.913259
C	-4.606183	-0.264941	-2.074277
C	3.763701	-1.850418	0.051224
C	4.541792	-1.451189	-1.213960
C	2.059681	-0.671209	2.416008
C	3.104534	-0.533970	3.531350
C	4.738233	-2.414065	1.093812
C	2.784384	-2.954467	-0.387391
C	0.900446	0.310306	2.664108
C	1.444683	-2.078790	2.461653
C	3.120518	2.338001	1.431488
C	5.240424	1.030927	1.198519
H	0.883656	5.010852	1.754230
H	1.240417	5.935099	-0.537332
H	0.070156	4.874890	-2.472955
H	-2.700574	4.104726	2.407685
H	-4.392568	3.617885	2.657041
H	-3.843204	4.152981	1.051585
H	-2.334059	-0.176070	2.791841
H	-3.472943	0.782404	3.769902
H	-1.799931	1.343305	3.541491
H	-5.568457	1.577968	1.725234
H	-5.163390	1.597533	0.022246
H	-6.254735	-0.580344	0.789258
H	-4.892955	-0.883759	1.862575
H	-4.196423	0.755324	-2.035070
H	-5.692384	-0.167899	-2.221182
H	-4.177241	-0.760362	-2.955336
H	-4.325501	-3.534413	-1.514200
H	-5.949040	-3.019967	-0.990478
H	-4.769540	-3.609572	0.205725
H	0.310849	-4.853960	-0.593201
H	-0.325776	-5.714110	1.660873
H	-1.981677	-4.442810	3.029196
H	-0.576582	3.047834	2.092825
H	-1.455372	2.964319	-2.115646
H	-0.711173	-2.770572	-1.470111
H	-2.981073	-2.352479	2.153552
H	3.666702	3.276424	1.253139
H	2.090466	2.493834	1.089740
H	3.120700	2.158464	2.511469
H	4.472571	2.677074	-0.819341
H	4.391888	1.045475	-1.503633
H	2.896341	1.960672	-1.208711
H	5.752688	2.005164	1.192771
H	5.231875	0.667932	2.232652
H	5.842899	0.343653	0.595090
H	4.993758	-2.364164	-1.629127
H	3.879668	-1.033628	-1.984605
H	5.352737	-0.742902	-1.019110
H	5.313179	-3.227844	0.625954
H	5.453408	-1.665929	1.452680
H	4.217397	-2.839803	1.958602
H	3.372544	-3.782441	-0.811317
H	2.179939	-3.353784	0.428474
H	2.099498	-2.592411	-1.166447
H	0.416551	0.030708	3.611216
H	1.221736	1.350730	2.745155
H	0.143891	0.248704	1.870853
H	0.909879	-2.179558	3.417287
H	0.712125	-2.235548	1.660237
H	2.195521	-2.874451	2.421650
H	2.619126	-0.778808	4.488391
H	3.949215	-1.219550	3.403362
H	3.495165	0.486031	3.616110
C	-0.161615	0.310596	-2.662634
O	-1.471896	0.472387	-2.593497
O	0.498933	0.355899	-3.689070

93

TSII (III)

C	2.354495	-4.255794	0.699727
C	3.054926	-3.373927	-0.139856
C	3.455701	-3.835983	-1.403182
C	3.170690	-5.138982	-1.812759
C	2.487738	-6.011235	-0.963340
C	2.081134	-5.561086	0.295222
N	3.333607	-2.032430	0.260338
SI	4.410863	-1.710499	1.633449
C	5.900855	-0.640026	1.150683
C	5.697940	0.865919	0.891139
SI	4.650806	1.308372	-0.620536
C	5.208913	0.251259	-2.079617
N	2.934069	0.958390	-0.357656
AL	2.330896	-0.733625	-0.481450
C	2.052453	2.038488	-0.059564
C	2.127292	2.706424	1.175342
C	1.295313	3.790169	1.453485
C	0.361934	4.222794	0.509140
C	0.269754	3.557462	-0.714438
C	1.106526	2.478656	-0.999336
C	4.846311	3.135649	-1.013322
AU	-1.845862	-0.043576	-0.633187
P	-3.609620	0.583419	0.778121
C	-2.872393	0.948296	2.511535
C	-2.111052	2.282466	2.446913
C	-4.873183	-0.851350	0.897308
C	-6.231660	-0.467036	1.497923

C	-4.468166	2.151823	0.090181
C	-3.374100	3.094348	-0.448417
C	-5.074005	-1.434122	-0.515797
C	-4.251955	-1.981962	1.734745
C	5.110341	-3.339698	2.264546
C	3.433514	-0.838483	2.993072
C	-5.317717	1.756446	-1.129279
C	-5.350605	2.899539	1.097835
C	-3.898949	1.004182	3.650333
C	-1.807018	-0.123097	2.824625
H	1.535423	-6.228329	0.964065
H	2.267820	-7.030656	-1.281168
H	3.488448	-5.473868	-2.801116
H	4.336492	-3.996886	2.680287
H	5.846180	-3.132712	3.055261
H	5.619295	-3.887303	1.458846
H	2.952955	0.073723	2.610316
H	4.085642	-0.547491	3.829975
H	2.644492	-1.496523	3.384488
H	6.614611	-0.779555	1.982746
H	6.375478	-1.118533	0.277499
H	6.677317	1.358346	0.756709
H	5.242112	1.350380	1.770953
H	5.035755	-0.818880	-1.891687
H	6.286392	0.379136	-2.261413
H	4.669265	0.533727	-2.994353
H	4.202000	3.425608	-1.854947
H	5.889637	3.348207	-1.287022
H	4.581922	3.764390	-0.152521
H	-0.455440	3.882747	-1.461095
H	-0.288936	5.070163	0.726776
H	1.375589	4.297031	2.416200
H	2.011960	-3.897716	1.671465
H	4.000125	-3.158245	-2.063223
H	1.041920	1.971102	-1.961665
H	2.854535	2.368469	1.915005
H	-4.904251	-2.863852	1.648627
H	-3.258888	-2.262270	1.359126
H	-4.175170	-1.733091	2.797917
H	-5.687389	-2.343613	-0.425807
H	-5.587050	-0.752903	-1.198502
H	-4.111708	-1.712736	-0.967354
H	-6.850913	-1.374675	1.568671
H	-6.142370	-0.047126	2.506083
H	-6.776016	0.249171	0.872539
H	-5.651701	2.682373	-1.621045
H	-4.732300	1.183994	-1.860771
H	-6.212845	1.185980	-0.861065
H	-5.832396	3.743333	0.579986
H	-6.144543	2.267484	1.511194
H	-4.772085	3.316387	1.929731
H	-3.868572	3.942919	-0.945579
H	-2.719217	3.494844	0.328268
H	-2.744872	2.580608	-1.187934
H	-1.294674	0.171104	3.753377
H	-2.222221	-1.122376	2.974505
H	-1.058673	-0.178397	2.021821
H	-1.548300	2.395375	3.385770
H	-1.384641	2.300046	1.624664
H	-2.773752	3.149421	2.357012
H	-3.374087	1.280205	4.578035
H	-4.679861	1.752961	3.476582
H	-4.381838	0.036453	3.826448
C	-0.221242	-0.553395	-1.791367
O	-0.053635	-0.357017	-2.985524
O	0.773638	-1.158241	-1.060606

93

PC (III)

C	2.756483	3.546840	0.169173
C	2.275848	2.430303	-0.542438
C	0.990975	2.522398	-1.111234
C	0.216470	3.671088	-0.959292
C	0.708092	4.771185	-0.252754
C	1.986766	4.701116	0.305240
N	3.040781	1.245219	-0.669107
SI	4.678326	1.318412	-1.331768
C	4.840193	2.869992	-2.390482
AL	2.370211	-0.307568	0.022385
AU	-1.909027	-0.382569	0.223633
P	-4.215328	-0.011442	0.208985
C	-5.143951	-1.669065	-0.026512
C	-4.379250	-2.507272	-1.070495
N	3.679774	-1.451866	0.586085
SI	4.877004	-0.738864	1.678594
C	5.532456	-2.047116	2.864752
C	3.798979	-2.775679	0.121116
C	5.048500	-3.341075	-0.210206
C	5.152128	-4.655005	-0.664406
C	4.013035	-5.447062	-0.820060
C	2.765927	-4.899472	-0.507397
C	2.659688	-3.592297	-0.038069
C	3.970052	0.580826	2.679455
C	6.331268	0.066324	0.764324
C	6.024840	1.376083	0.008403
C	4.942990	-0.190766	-2.429764
C	-4.599357	1.176742	-1.244918
C	-4.478715	0.396881	-2.565137
C	-4.709399	0.797858	1.872265
C	-6.216946	0.794521	2.155410
C	-5.979718	1.841642	-1.171856
C	-3.502571	2.259865	-1.295079
C	-3.962002	0.069278	3.007521
C	-4.192278	2.246911	1.891637
C	-5.075940	-2.467443	1.286295
C	-6.611145	-1.521011	-0.449865
H	1.862269	-5.501259	-0.617951
H	4.094837	-6.472756	-1.180081
H	6.136127	-5.058274	-0.909441
H	4.699529	-2.587222	3.336755
H	6.118794	-1.560088	3.657722
H	6.176839	-2.784014	2.368909
H	3.521961	1.366261	2.054631
H	4.685662	1.072608	3.355552
H	3.175076	0.133468	3.292130
H	7.126096	0.234781	1.512914
H	6.742151	-0.679370	0.064225
H	6.940465	1.747988	-0.485154
H	5.737553	2.164490	0.724212
H	4.816557	-1.135784	-1.885150
H	5.962171	-0.178517	-2.844814
H	4.233583	-0.180009	-3.269572
H	4.016608	2.929542	-3.116119
H	5.787776	2.837352	-2.947749
H	4.828366	3.789260	-1.790566
H	-0.777587	3.711232	-1.407612
H	0.104385	5.672072	-0.139686
H	2.387021	5.549881	0.862323
H	1.679961	-3.189074	0.219199
H	5.949761	-2.734265	-0.122193
H	0.611742	1.675709	-1.684977
H	3.744768	3.495736	0.628489
H	-5.461938	-3.477689	1.084393
H	-4.043415	-2.572017	1.644942
H	-5.689523	-2.035258	2.083053
H	-4.850720	-3.500423	-1.119005
H	-4.399790	-2.079369	-2.075232
H	-3.329156	-2.641311	-0.776337
H	-7.060384	-2.524587	-0.499024

H	-7.196677	-0.930213	0.263370
H	-6.716529	-1.069292	-1.442301
H	-4.533536	1.122795	-3.389835
H	-3.513845	-0.121565	-2.642210
H	-5.286805	-0.326868	-2.711280
H	-6.120283	2.449490	-2.078677
H	-6.796908	1.113043	-1.131278
H	-6.072223	2.514876	-0.312533
H	-3.664475	2.863990	-2.200629
H	-3.514173	2.935677	-0.437264
H	-2.503532	1.807201	-1.359923
H	-4.157072	0.612050	3.944681
H	-4.283542	-0.964915	3.149263
H	-2.877362	0.072665	2.831878
H	-4.328086	2.637434	2.911051
H	-3.120834	2.298213	1.656565
H	-4.741468	2.906332	1.212059
H	-6.393905	1.328264	3.101627
H	-6.793058	1.303828	1.375053
H	-6.616717	-0.218554	2.274963
C	0.125881	-0.518398	0.194480
O	0.874009	-0.016921	1.135510
O	0.816234	-0.976883	-0.818715

93

PC_CO (III)

C	2.522241	3.471507	0.775896
C	1.902756	2.458160	0.020050
C	0.578255	2.669863	-0.397765
C	-0.102837	3.840317	-0.067571
C	0.525764	4.840132	0.678297
C	1.844837	4.647724	1.094669
N	2.581262	1.254310	-0.301269
SI	4.085643	1.340840	-1.239474
C	4.127555	3.001727	-2.131742
AL	1.992798	-0.317914	0.406317
AU	-1.465784	-0.335419	0.536374
P	-3.649038	-0.089406	0.070865
C	-4.517311	-1.791016	0.260953
C	-3.605465	-2.881189	-0.336474
N	3.363866	-1.532834	0.509964
SI	4.763641	-1.018594	1.453570
C	5.545790	-2.458525	2.383146
C	3.281785	-2.746849	-0.186731
C	4.426643	-3.434463	-0.644023
C	4.316921	-4.631369	-1.349853
C	3.065579	-5.178029	-1.642133
C	1.920858	-4.502723	-1.208264
C	2.023128	-3.314880	-0.489080
C	4.075144	0.197693	2.725921
C	6.061703	-0.153070	0.379570
C	5.653082	1.229969	-0.168675
C	4.088517	-0.029962	-2.531258
C	-3.854855	0.531004	-1.734135
C	-3.528614	-0.624026	-2.696045
C	-4.437542	1.173173	1.280798
C	-5.971670	1.156033	1.311298
C	-5.245513	1.088340	-2.066638
C	-2.792001	1.612195	-2.005081
C	-3.895471	0.906044	2.698779
C	-3.949943	2.582831	0.903580
C	-4.639760	-2.121017	1.758008
C	-5.907052	-1.874512	-0.384884
H	0.932180	-4.913361	-1.420993
H	2.983402	-6.111940	-2.198111
H	5.224938	-5.135309	-1.685363
H	4.769701	-3.068219	2.867017
H	6.206726	-2.061547	3.167854
H	6.142992	-3.116305	1.739721
H	3.547808	1.050894	2.273561
H	4.900750	0.616582	3.320872
H	3.386730	-0.308144	3.418158
H	6.989634	-0.072302	0.972820
H	6.302540	-0.829780	-0.456980
H	6.469530	1.645783	-0.785908
H	5.525803	1.942966	0.663385
H	4.039973	-1.032913	-2.088317
H	5.010658	0.028284	-3.129215
H	3.235045	0.088615	-3.214081
H	3.187200	3.177924	-2.673043
H	4.950350	3.003219	-2.861503
H	4.280928	3.841902	-1.442007
H	-1.132054	3.976068	-0.402935
H	-0.005943	5.756985	0.933498
H	2.349172	5.415130	1.684004
H	1.120959	-2.814234	-0.125467
H	5.415333	-3.014904	-0.458962
H	0.091183	1.892870	-0.986117
H	3.546046	3.322306	1.122168
H	-4.966510	-3.167853	1.846266
H	-3.674334	-2.027622	2.272480
H	-5.381475	-1.501655	2.272271
H	-4.064860	-3.858711	-0.125109
H	-3.477638	-2.799838	-1.418361
H	-2.611213	-2.859841	0.129435
H	-6.334035	-2.863370	-0.157879
H	-6.599186	-1.119101	0.002484
H	-5.868452	-1.781486	-1.475664
H	-3.468224	-0.209587	-3.713392
H	-2.556868	-1.078796	-2.462295
H	-4.295764	-1.404563	-2.704969
H	-5.268586	1.341231	-3.137612
H	-6.047397	0.365890	-1.880441
H	-5.469507	2.006304	-1.512149
H	-2.866614	1.907819	-3.062431
H	-2.916038	2.510108	-1.395978
H	-1.784803	1.213893	-1.829845
H	-4.264597	1.708843	3.355024
H	-4.224372	-0.046343	3.119974
H	-2.799188	0.936946	2.712094
H	-4.270262	3.272593	1.698550
H	-2.854307	2.626640	0.850174
H	-4.377637	2.945879	-0.036506
H	-6.311058	1.958622	1.983777
H	-6.419842	1.337164	0.328547
H	-6.369320	0.214124	1.705052
O	0.458031	-0.660176	0.998845
C	-0.462743	2.269149	2.817307
O	0.572373	2.100292	3.256177

77

Complex [^{t}Bu$_3$PAu-**IV**]

Al	-2.024485	-0.087952	-0.124350
Au	0.362928	0.174193	-0.089056
C	-3.431320	3.314669	0.938784
C	-3.056430	2.473842	-0.123323
C	-2.203561	2.983135	-1.114428
C	-1.714995	4.289675	-1.034860
C	-2.090420	5.115683	0.023023
C	-2.955033	4.620873	1.006650
N	-3.423160	1.101586	-0.155230
P	2.764070	0.226807	0.069669
C	3.365778	2.035946	0.268066
C	2.544952	2.914929	-0.698350
C	-4.782475	0.731458	-0.128631
C	-5.098232	-0.687033	0.002758
C	-4.288841	-1.785753	0.091383
N	-2.895583	-1.717070	0.039226

C	-2.073148	-2.839893	0.299092
C	-1.055023	-3.163551	-0.616202
C	-0.146709	-4.186593	-0.346802
C	-0.241631	-4.919757	0.836747
C	-1.252017	-4.609416	1.752056
C	-2.150615	-3.576190	1.495210
C	3.529867	-0.543984	-1.510216
C	3.387459	0.458051	-2.667675
C	3.269193	-0.809384	1.603004
C	4.709281	-0.594007	2.085261
C	5.001364	-0.957259	-1.380877
C	2.675553	-1.765590	-1.904454
C	2.283426	-0.482293	2.742851
C	3.056304	-2.299529	1.285567
C	3.004966	2.521325	1.682342
C	4.865619	2.252953	0.028671
H	0.634459	-4.415955	-1.073088
H	0.464601	-5.723319	1.046749
H	-1.328680	-5.164901	2.688033
H	-0.993668	-2.603625	-1.550165
H	-2.901785	-3.309456	2.238345
H	3.187897	3.605435	1.724450
H	1.943386	2.353643	1.907789
H	3.615013	2.054980	2.462803
H	2.784779	3.968433	-0.488530
H	2.766176	2.726999	-1.751612
H	1.465973	2.773043	-0.541389
H	5.101843	3.311224	0.220090
H	5.487732	1.648138	0.698225
H	5.159825	2.036319	-1.004326
H	3.665898	-0.058380	-3.598550
H	2.351164	0.805030	-2.776074
H	4.045768	1.326471	-2.565018
H	5.347776	-1.332837	-2.356400
H	5.648667	-0.119174	-1.099170
H	5.144678	-1.763484	-0.653032
H	3.028582	-2.130066	-2.881302
H	2.744327	-2.592068	-1.193717
H	1.616277	-1.489123	-2.001657
H	2.487844	-1.169035	3.578356
H	2.375521	0.538832	3.120167
H	1.244363	-0.637802	2.418791
H	3.175677	-2.862216	2.223728
H	2.043180	-2.495730	0.909539
H	3.787949	-2.694366	0.573061
H	4.899636	-1.272766	2.931063
H	5.450215	-0.815349	1.308880
H	4.880184	0.427322	2.443832
H	-6.167459	-0.895345	-0.015841
C	-4.926080	-3.151562	0.146576
H	-1.923352	2.338989	-1.949219
H	-1.044949	4.662105	-1.811102
H	-1.715559	6.137832	0.084611
H	-3.249519	5.256786	1.842984
H	-4.089756	2.923107	1.714150
C	-5.803788	1.628041	-0.265061
H	-6.831975	1.273184	-0.251821
H	-5.630895	2.693041	-0.394048
H	-5.998176	-3.074185	-0.063175
H	-4.465825	-3.822253	-0.594141
H	-4.803555	-3.634411	1.125639

80

RC (IV)

C	-2.019504	3.010413	0.839517
C	-2.951603	2.450343	-0.048594
C	-3.366573	3.213275	-1.155629
C	-2.851871	4.489213	-1.366915
C	-1.908646	5.032334	-0.486219
C	-1.494013	4.285453	0.614912
N	-3.357603	1.100600	0.119962
AL	-2.001165	-0.138557	0.113224
AU	0.390771	0.057421	0.242492
P	2.789959	0.035960	0.455036
C	3.423179	1.773046	0.960351
C	2.635494	2.819263	0.145934
C	-4.726984	0.772344	0.175481
C	-5.090458	-0.639553	0.111961
C	-4.319404	-1.768732	0.082624
N	-2.923950	-1.743483	0.085021
C	-2.142273	-2.917238	0.211815
C	-1.114828	-3.151832	-0.720427
C	-0.241214	-4.228127	-0.569978
C	-0.382709	-5.104721	0.506587
C	-1.404002	-4.884856	1.435961
C	-2.267970	-3.800047	1.299557
C	3.564820	-0.479124	-1.220323
C	3.458004	0.705712	-2.194658
C	3.246491	-1.248246	1.805074
C	4.682662	-1.146960	2.335203
C	5.026055	-0.940882	-1.145786
C	2.691838	-1.598606	-1.822849
C	2.247005	-1.091806	2.969799
C	3.009457	-2.660089	1.242762
C	3.048153	2.021690	2.430628
C	4.931150	1.994242	0.782250
H	0.547840	-4.386456	-1.306560
H	0.296086	-5.949680	0.623445
H	-1.517213	-5.555069	2.289340
H	-1.018628	-2.478634	-1.573163
H	-3.028200	-3.610061	2.056919
H	3.253472	3.078567	2.657168
H	1.979063	1.844106	2.609142
H	3.632645	1.416370	3.130944
H	2.889558	3.816890	0.535756
H	2.869621	2.810040	-0.920797
H	1.551470	2.675649	0.257490
H	5.183121	3.000510	1.151654
H	5.529869	1.272867	1.349774
H	5.239154	1.947458	-0.268246
H	3.732254	0.344993	-3.197413
H	2.433339	1.096190	-2.248467
H	4.136649	1.527322	-1.944652
H	5.379403	-1.153295	-2.166835
H	5.685152	-0.176479	-0.719069
H	5.142765	-1.861823	-0.563686
H	3.053741	-1.805206	-2.841532
H	2.729669	-2.532762	-1.257853
H	1.640280	-1.284533	-1.887511
H	2.423530	-1.911097	3.683295
H	2.351620	-0.149277	3.511862
H	1.210767	-1.170511	2.610139
H	3.105578	-3.374059	2.074579
H	1.997724	-2.769301	0.829460
H	3.742286	-2.945389	0.480913
H	4.845034	-1.957922	3.062184
H	5.431097	-1.257204	1.542393
H	4.869569	-0.201269	2.856153
H	-6.166407	-0.808278	0.079464
C	-5.000526	-3.108186	-0.040333
H	-4.088107	2.783953	-1.850457
H	-3.179285	5.062416	-2.235665
H	-1.503714	6.029394	-0.660851
H	-0.762594	4.696197	1.312454
H	-1.707303	2.429980	1.708746
C	-5.713750	1.707425	0.307537
H	-6.752088	1.386551	0.355189
H	-5.502681	2.771411	0.373121

H	-6.063444	-2.969584	-0.264446
H	-4.541917	-3.702046	-0.844831
H	-4.919855	-3.707290	0.877083
C	-0.743844	1.737742	-2.766048
O	0.067925	2.557962	-2.576623
O	-1.548087	0.911756	-2.974875

80

TSI (IV)

C	-1.784524	3.074781	0.774338
C	-2.572550	2.520151	-0.251967
C	-2.697859	3.241754	-1.454098
C	-2.072262	4.476254	-1.610859
C	-1.287481	5.014271	-0.586416
C	-1.145205	4.301707	0.605362
N	-3.117574	1.230091	-0.087762
AL	-1.973482	-0.162366	0.325792
AU	0.431923	-0.002894	0.433256
P	2.752938	0.115454	-0.219641
C	3.298930	1.952714	-0.222818
C	4.591356	2.243427	-0.996118
N	-2.807215	-1.632294	-0.397388
C	-1.988944	-2.747944	-0.698434
C	-1.868848	-3.248201	-2.007946
C	-0.960671	-4.261430	-2.298526
C	-0.144973	-4.799376	-1.295619
C	-0.259550	-4.315796	0.006296
C	-1.180193	-3.308111	0.304750
O	-2.361477	-0.346276	2.487248
C	-1.217256	-0.218561	2.842458
O	-0.282572	-0.128816	3.543950
C	2.884764	-0.616262	-1.986123
C	2.007245	-1.882019	-2.055266
C	3.838386	-0.898636	0.991453
C	3.582271	-2.393941	0.737763
C	2.259266	0.384192	-2.972911
C	4.312364	-0.947459	-2.440299
C	5.343874	-0.619017	0.900052
C	3.340934	-0.623938	2.424763
C	2.144545	2.799821	-0.796612
C	3.466899	2.407823	1.236558
H	-0.875863	-4.626852	-3.323181
H	0.569044	-5.589092	-1.530883
H	0.361181	-4.729606	0.802389
H	-0.539168	4.706694	1.417037
H	-0.792507	5.976619	-0.717764
H	-2.182180	5.015034	-2.553349
H	-2.490509	-2.820069	-2.793898
H	-1.288230	-2.952485	1.329977
H	-1.691788	2.537270	1.718423
H	-3.264552	2.807719	-2.277807
H	4.055891	-2.960473	1.553473
H	2.509290	-2.628842	0.744298
H	4.017180	-2.746892	-0.202761
H	3.865356	-1.311186	3.106004
H	3.535233	0.395398	2.766214
H	2.262588	-0.815239	2.513370
H	5.868883	-1.293846	1.593976
H	5.743159	-0.799491	-0.104276
H	5.594950	0.406989	1.191928
H	3.601857	3.499663	1.237063
H	2.573508	2.182221	1.834358
H	4.343193	1.968314	1.724175
H	4.837053	3.310393	-0.879164
H	5.443665	1.663453	-0.624122
H	4.484511	2.050463	-2.069513
H	2.398451	3.861414	-0.655023
H	1.974575	2.639494	-1.863621
H	1.200941	2.606521	-0.267858
H	1.964103	-2.209878	-3.104878
H	2.391145	-2.716060	-1.464634
H	0.979442	-1.680228	-1.722870
H	2.177221	-0.113744	-3.950479
H	1.247828	0.678376	-2.661839
H	2.867090	1.283969	-3.112110
H	4.273110	-1.302792	-3.481782
H	4.976318	-0.076078	-2.411723
H	4.764552	-1.746406	-1.842030
C	-4.431214	0.927234	-0.456351
C	-4.166920	-1.618238	-0.758245
C	-4.876829	-0.343047	-0.698561
H	-5.948360	-0.437094	-0.873603
C	-5.426564	2.062192	-0.458638
H	-6.447653	1.667052	-0.491016
H	-5.308781	2.674600	0.447772
H	-5.296163	2.738071	-1.314240
C	-4.853007	-2.741599	-1.121497
H	-5.905039	-2.666336	-1.387251
H	-4.382468	-3.720575	-1.161810

80

INT (IV)

C	2.312582	-2.363195	1.797222
C	2.594825	-2.096570	0.443604
C	2.435558	-3.142951	-0.484759
C	2.029732	-4.408903	-0.068044
C	1.747163	-4.660886	1.276577
C	1.885443	-3.625839	2.203673
N	2.938664	-0.784407	0.054365
AL	1.940453	0.652063	0.615260
AU	-0.512036	0.027265	0.884604
P	-2.103126	-0.762946	-0.719349
C	-2.775614	-2.424201	-0.047058
C	-3.595696	-3.229337	-1.063421
N	2.176105	1.962655	-0.639265
C	1.215457	2.999766	-0.737227
C	0.638144	3.376851	-1.964326
C	-0.373716	4.330832	-2.008975
C	-0.846566	4.928050	-0.835419
C	-0.281504	4.562160	0.385171
C	0.746564	3.618745	0.434706
O	1.864482	1.032012	2.382023
C	0.580141	0.706413	2.546233
O	-0.019909	0.774479	3.603490
C	-1.328107	-1.006212	-2.448582
C	-0.370799	0.165569	-2.726885
C	-3.489184	0.555993	-0.781106
C	-2.984537	1.764422	-1.586486
C	-0.457976	-2.271529	-2.431279
C	-2.362415	-1.113342	-3.577820
C	-4.810251	0.054633	-1.378344
C	-3.728746	1.077091	0.650655
C	-1.582510	-3.264497	0.450292
C	-3.644045	-2.143659	1.190749
H	-0.808270	4.603093	-2.972231
H	-1.641450	5.672982	-0.875948
H	-0.626484	5.023858	1.311059
H	1.674005	-3.802979	3.258966
H	1.421996	-5.650778	1.596790
H	1.914118	-5.202461	-0.808040
H	0.990827	2.908915	-2.881590
H	1.206245	3.367181	1.390852
H	2.452094	-1.572101	2.534385
H	2.605074	-2.949235	-1.542828
H	-3.720295	2.574034	-1.470933
H	-2.024890	2.140400	-1.209555
H	-2.890799	1.557953	-2.657273
H	-4.459548	1.897669	0.593754

H	-4.126476	0.324085	1.334230
H	-2.805716	1.488977	1.084951
H	-5.514784	0.899211	-1.417344
H	-4.692226	-0.326064	-2.398829
H	-5.274440	-0.728003	-0.767994
H	-3.887469	-3.110760	1.654800
H	-3.108568	-1.546004	1.941042
H	-4.589718	-1.648474	0.948405
H	-3.976379	-4.132328	-0.562311
H	-4.459307	-2.671389	-1.442246
H	-2.995188	-3.560444	-1.917641
H	-1.982953	-4.162188	0.944734
H	-0.911756	-3.595123	-0.344678
H	-0.979809	-2.717529	1.188749
H	0.119693	-0.020041	-3.694143
H	-0.868609	1.134602	-2.784952
H	0.415538	0.233896	-1.963587
H	0.117163	-2.293400	-3.368837
H	0.260873	-2.260161	-1.603302
H	-1.042708	-3.195742	-2.383155
H	-1.825299	-1.320960	-4.515652
H	-3.078230	-1.927558	-3.418743
H	-2.921259	-0.182468	-3.722714
C	3.933626	-0.526938	-0.899807
C	3.263193	1.887075	-1.538819
C	4.059696	0.663263	-1.559131
H	4.941514	0.745655	-2.193486
C	3.647551	2.924263	-2.336171
H	3.132856	3.880689	-2.342174
H	4.514507	2.806432	-2.982036
C	5.006782	-1.572002	-1.088073
H	5.863759	-1.132067	-1.609033
H	5.341094	-1.953520	-0.112158
H	4.668722	-2.440742	-1.667264

80

TSII (IV)

C	-1.396644	-3.281673	0.550836
C	-1.901260	-2.770983	-0.650855
C	-1.228858	-3.060008	-1.849658
C	-0.083172	-3.849574	-1.836965
C	0.423037	-4.348591	-0.631492
C	-0.231997	-4.052936	0.561605
N	-3.033538	-1.906739	-0.631603
C	-4.181679	-2.219410	-1.400590
C	-4.344762	-3.418644	-2.024984
AL	-2.995367	-0.405804	0.360176
N	-4.403452	0.623741	-0.116179
C	-4.440658	2.009443	0.198039
C	-4.495100	2.989763	-0.806519
C	-4.485210	4.342279	-0.472398
C	-4.394646	4.741173	0.863962
C	-4.312923	3.771259	1.864675
C	-4.342780	2.416246	1.536954
O	-1.747423	0.140538	1.370559
C	-0.577810	-0.315483	1.962782
O	-0.651118	-0.808610	3.074064
AU	1.135736	-0.039400	0.858316
P	3.133051	0.276158	-0.329529
C	3.947963	-1.421622	-0.685880
C	3.767657	-2.311035	0.560960
C	2.755186	1.174112	-1.978534
C	1.480969	0.550651	-2.584285
C	2.401013	2.638781	-1.670988
C	3.893853	1.138351	-3.006491
C	4.305395	1.346645	0.744855
C	3.475178	2.466404	1.403971
C	4.835138	0.482753	1.902100
C	5.490146	1.961525	-0.011862
C	3.167512	-2.117353	-1.812421
C	5.431182	-1.353497	-1.071230
C	-5.246890	-1.223211	-1.479595
C	-5.370385	0.028608	-0.946465
C	-6.654450	0.787776	-1.158864
H	4.139043	-3.317937	0.316034
H	4.318792	-1.955654	1.434862
H	2.706371	-2.395955	0.831994
H	3.531197	-3.153735	-1.882649
H	2.093729	-2.162181	-1.594403
H	3.320401	-1.649929	-2.790304
H	5.774796	-2.372546	-1.306830
H	5.606232	-0.732167	-1.956747
H	6.060436	-0.979720	-0.256210
H	5.361625	1.146366	2.604224
H	4.016587	-0.000848	2.451647
H	5.547469	-0.281705	1.576050
H	4.127625	3.000450	2.111475
H	3.085931	3.198323	0.692803
H	2.628659	2.050107	1.967435
H	6.124487	2.496469	0.711668
H	6.113768	1.207347	-0.504259
H	5.169937	2.691328	-0.763853
H	3.589033	1.728503	-3.884473
H	4.821646	1.574541	-2.620341
H	4.109550	0.123176	-3.356859
H	1.194953	1.146369	-3.464531
H	1.611490	-0.484041	-2.908691
H	0.651192	0.579965	-1.864481
H	2.014674	3.094121	-2.595049
H	1.614532	2.711669	-0.908012
H	3.265506	3.229497	-1.351280
H	-4.522934	2.682960	-1.851996
H	-4.530622	5.090993	-1.264428
H	-4.380561	5.800303	1.121735
H	-4.235024	4.068559	2.911075
H	-4.293498	1.658049	2.319946
H	-1.616271	-2.658658	-2.785771
H	0.429697	-4.066017	-2.775282
H	1.327675	-4.956999	-0.626051
H	0.154236	-4.426694	1.510137
H	-1.915993	-3.063162	1.485335
H	-6.115376	-1.573937	-2.035800
H	-7.409280	0.127447	-1.597997
H	-7.032820	1.176603	-0.202087
H	-6.528839	1.653452	-1.822598
H	-3.598524	-4.206722	-1.977309
H	-5.253358	-3.605499	-2.592414

80

PC (IV)

C	-2.552886	3.049853	-1.556047
C	-2.563949	2.368693	-0.325462
C	-1.515574	2.606870	0.579766
C	-0.488207	3.495595	0.264826
C	-0.493571	4.178429	-0.952433
C	-1.534415	3.951348	-1.857647
N	-3.534745	1.382751	-0.015741
C	-4.906414	1.644989	-0.068598
C	-5.334121	3.088946	-0.136413
C	-5.868066	0.676771	0.030902
C	-5.776419	-0.775325	0.154420
C	-6.922655	-1.499495	0.306848
N	-4.493452	-1.366712	0.144672
AL	-2.988539	-0.357667	0.159613
AU	1.255406	-0.488222	0.102778
P	3.523591	0.048856	-0.039167
C	3.756306	1.190651	-1.559387
C	3.176870	2.578771	-1.237306

C	-4.349941	-2.776885	0.046831
C	-4.916607	-3.504463	-1.014525
C	-4.680502	-4.870309	-1.137152
C	-3.869367	-5.539492	-0.212821
C	-3.301087	-4.824407	0.840064
C	-3.548697	-3.455983	0.976662
C	4.553313	-1.551930	-0.241811
C	6.055590	-1.377835	0.017699
C	4.032652	0.969929	1.560233
C	2.896811	1.939177	1.944787
C	3.977859	-2.619570	0.710496
C	4.343549	-2.098375	-1.663881
C	4.126411	-0.046685	2.710264
C	5.356909	1.737876	1.455162
C	5.213744	1.344053	-2.013840
C	2.902090	0.638998	-2.718645
H	0.314450	3.662043	0.984618
H	0.306399	4.877543	-1.197383
H	-1.543596	4.467422	-2.818831
H	-1.516476	2.083217	1.535892
H	-3.334325	2.842905	-2.287024
H	4.799372	-3.098432	-1.709784
H	3.277503	-2.207661	-1.903012
H	4.821844	-1.484329	-2.433545
H	4.490126	-3.571020	0.502648
H	4.123586	-2.386970	1.767622
H	2.903047	-2.767044	0.535169
H	6.555570	-2.337875	-0.181681
H	6.510661	-0.625308	-0.635727
H	6.271381	-1.109408	1.057774
H	4.249329	0.516493	3.647194
H	3.209264	-0.643828	2.799178
H	4.984745	-0.719485	2.617345
H	5.582009	2.176516	2.439234
H	6.197694	1.091423	1.180376
H	5.304750	2.562914	0.736392
H	3.143666	2.380734	2.922066
H	2.763698	2.756907	1.233301
H	1.939299	1.408798	2.041596
H	2.959836	1.356077	-3.551243
H	3.241155	-0.330628	-3.089934
H	1.847936	0.545973	-2.423056
H	3.170534	3.161397	-2.170588
H	2.139626	2.515573	-0.882473
H	3.775537	3.134941	-0.508888
H	5.241489	2.059070	-2.850040
H	5.862930	1.734538	-1.222365
H	5.639482	0.402473	-2.377629
H	-6.893192	1.043969	0.064102
H	-3.120700	-2.900957	1.813230
H	-2.669817	-5.331500	1.571025
H	-3.684816	-6.609158	-0.316632
H	-5.122517	-5.418680	-1.970380
H	-5.536713	-2.981428	-1.742065
H	-7.881661	-0.986683	0.313060
H	-6.918336	-2.579374	0.426239
H	-6.405857	3.172934	0.071652
H	-4.779050	3.690154	0.598938
H	-5.140506	3.537819	-1.120146
C	-0.769537	-0.723033	0.166682
O	-1.486542	-0.619875	1.258158
O	-1.505292	-0.772320	-0.911956

80

PC_CO (IV)

C	-1.666113	3.724919	-0.976505
C	-1.983520	2.913673	0.129989
C	-1.379371	3.211113	1.367415
C	-0.511630	4.293518	1.493963
C	-0.235062	5.117050	0.399552
C	-0.818364	4.821038	-0.834742
N	-2.808195	1.776437	0.015312
C	-3.944415	1.761169	-0.808044
C	-4.663070	3.070782	-1.034183
C	-4.503475	0.614070	-1.295851
C	-4.092390	-0.785174	-1.204967
C	-4.834435	-1.725614	-1.855961
N	-2.983795	-1.096720	-0.387200
AL	-2.320485	0.167744	0.752692
AU	0.351408	-0.103139	1.320110
P	2.290391	-0.085501	0.153404
C	2.017075	0.983574	-1.412368
C	1.955541	2.463671	-1.003784
C	-2.345774	-2.359125	-0.465458
C	-2.016678	-2.952952	-1.698491
C	-1.322493	-4.158860	-1.740999
C	-0.921112	-4.795519	-0.562178
C	-1.227171	-4.205364	0.663622
C	-1.939265	-3.006003	0.714666
C	2.755660	-1.873443	-0.345618
C	4.209180	-2.036539	-0.811155
C	3.669066	0.681248	1.241011
C	3.066924	1.868545	2.019484
C	2.479845	-2.802324	0.852660
C	1.809395	-2.343545	-1.461907
C	4.104117	-0.343033	2.302688
C	4.904765	1.144776	0.457963
C	3.098808	0.796412	-2.485727
C	0.634179	0.659917	-2.013307
H	-0.056989	4.501124	2.463715
H	0.433987	5.971202	0.504350
H	-0.592781	5.437177	-1.706562
H	-1.597142	2.575647	2.226687
H	-2.066703	3.471832	-1.957051
H	1.977177	-3.420178	-1.611815
H	0.755640	-2.219315	-1.183388
H	1.993135	-1.845245	-2.419110
H	2.704502	-3.832853	0.538804
H	3.087785	-2.579025	1.732358
H	1.420065	-2.763983	1.135872
H	4.350976	-3.079136	-1.133891
H	4.453983	-1.391480	-1.661910
H	4.931592	-1.843431	-0.010563
H	4.779063	0.170398	3.003797
H	3.249182	-0.716461	2.879693
H	4.653737	-1.190754	1.881554
H	5.652334	1.508650	1.179195
H	5.366757	0.333960	-0.116145
H	4.682853	1.971582	-0.225093
H	3.845129	2.262024	2.690933
H	2.728467	2.686752	1.379767
H	2.217387	1.544440	2.635672
H	0.499662	1.294261	-2.902169
H	0.512963	-0.381037	-2.319716
H	-0.167919	0.908442	-1.306340
H	1.628357	3.040205	-1.881049
H	1.217434	2.637774	-0.210931
H	2.924966	2.864051	-0.690248
H	2.896906	1.505903	-3.302283
H	4.107033	1.002693	-2.110681
H	3.088553	-0.209594	-2.918991
H	-5.446568	0.747369	-1.825399
H	-2.176968	-2.551146	1.677177
H	-0.921842	-4.683991	1.595303
H	-0.375929	-5.738723	-0.601732
H	-1.078998	-4.600041	-2.708877
H	-2.312951	-2.457057	-2.621865
H	-5.692300	-1.412571	-2.446720

H	-4.608465	-2.786970	-1.805056
H	-5.694434	2.876385	-1.347818
H	-4.678069	3.663211	-0.108042
H	-4.190084	3.695149	-1.803069
O	-1.484512	-0.056590	2.222142
C	0.881981	-1.611701	3.868321
O	0.190609	-2.491411	4.073617

81

Complex [^{t}Bu$_3$PAu-**V**]

C	-5.160868	3.273432	-0.423890
C	-4.301337	2.753609	-1.587975
C	-2.943803	3.474383	-1.584170
C	-2.228607	3.201319	-0.251531
C	-3.084417	3.728401	0.910775
C	-4.441082	3.006510	0.908519
C	-4.078584	1.241296	-1.423890
C	-3.364085	0.939876	-0.087238
C	-2.022285	1.690338	-0.085496
C	-4.217844	1.494452	1.075748
N	-3.107601	-0.496699	0.069119
C	-4.184827	-1.372050	0.130781
C	-3.963698	-2.707692	0.263742
C	-2.534235	-3.176632	0.331470
SI	-2.147908	-3.983390	1.969628
H	-3.031795	-5.170489	2.243361
AL	-1.516316	-1.452889	0.186559
AU	0.756241	-0.640630	0.153623
SI	-2.119837	-4.255304	-1.135400
H	-3.005414	-5.469737	-1.215154
H	-2.307696	-3.494696	-2.419330
H	-0.697984	-4.742172	-1.092376
H	-2.357440	-3.015741	3.102046
H	-0.724249	-4.462327	2.031714
H	-4.801040	-3.407927	0.315393
H	-5.197852	-0.966518	0.070255
H	-1.386282	1.297894	-0.896999
H	-1.496760	1.477415	0.860153
H	-5.043518	0.712683	-1.456556
H	-3.461747	0.846902	-2.247122
H	-3.700551	1.279425	2.024377
H	-5.187694	0.975670	1.108307
H	-1.246506	3.700306	-0.246433
H	-2.325718	3.118752	-2.424275
H	-3.089643	4.558156	-1.719483
H	-4.817522	2.942441	-2.542168
H	-5.343016	4.353471	-0.543230
H	-6.142629	2.773493	-0.428332
H	-5.058201	3.375095	1.742743
H	-3.233243	4.815330	0.808177
H	-2.567092	3.556316	1.868315
P	2.927533	0.417182	0.090809
C	2.693830	2.295908	0.418984
C	4.063064	-0.337944	1.437308
C	3.709543	0.159165	-1.640871
C	4.879357	1.092995	-1.974589
C	4.179813	-1.301067	-1.753112
C	2.593507	0.321936	-2.693142
C	3.978301	3.060231	0.766576
C	2.046916	2.933852	-0.821978
C	1.666019	2.462643	1.556903
C	5.558284	-0.034897	1.278269
C	3.589866	0.162894	2.812136
C	3.832805	-1.862778	1.443358
H	3.001796	0.032474	-3.673530
H	1.742066	-0.335992	-2.468273
H	2.220441	1.345317	-2.777387
H	4.459906	-1.486579	-2.800845
H	5.057745	-1.516505	-1.135799
H	3.377423	-2.004312	-1.493017
H	5.279092	0.816091	-2.962674
H	4.570715	2.142762	-2.032743
H	5.698580	1.013580	-1.251161
H	6.098298	-0.471956	2.132731
H	5.978993	-0.476989	0.368326
H	5.768148	1.040556	1.268420
H	4.117367	-0.417489	3.583940
H	3.818146	1.219849	2.982993
H	2.512638	0.001200	2.951935
H	4.371607	-2.287926	2.303818
H	2.764818	-2.097734	1.556754
H	4.197462	-2.362032	0.542666
H	1.415776	3.531582	1.637731
H	0.742459	1.910608	1.334410
H	2.035775	2.132915	2.530413
H	3.730942	4.127429	0.878819
H	4.417363	2.727942	1.713954
H	4.741278	2.976911	-0.015255
H	1.752937	3.961440	-0.560245
H	2.728521	2.995964	-1.676280
H	1.139225	2.394528	-1.124898

84

RC (V)

C	-4.210851	-1.561497	-1.404281
C	-4.486183	-3.064800	-1.235395
C	-5.269634	-3.299916	0.066983
C	-4.445338	-2.795887	1.263230
C	-4.173190	-1.292760	1.096149
C	-3.391290	-1.022520	-0.209508
C	-3.153376	-3.827218	-1.174603
C	-2.331730	-3.314833	0.018390
C	-3.109496	-3.553769	1.322322
C	-2.073646	-1.812173	-0.150034
N	-3.086572	0.404910	-0.359210
AL	-1.471670	1.327041	-0.345689
AU	0.794493	0.497478	-0.215241
P	3.008379	-0.484710	-0.254126
C	4.017485	0.043091	1.289270
C	3.492037	-0.725956	2.512687
C	-4.134898	1.305157	-0.506445
C	-3.872471	2.632229	-0.647032
C	-2.430063	3.065352	-0.634275
SI	-2.073899	4.297248	0.720567
SI	-1.941297	3.701712	-2.323695
C	2.836804	-2.395138	-0.293265
C	2.308891	-2.813361	-1.675957
C	3.897515	0.117513	-1.843999
C	4.294666	1.590361	-1.649873
C	4.126780	-3.169687	0.007385
C	1.738409	-2.795792	0.711582
C	5.135724	-0.692173	-2.248816
C	2.872114	0.109429	-2.995911
C	5.533424	-0.166470	1.180841
C	3.709439	1.528990	1.563467
H	-2.797117	4.860348	-2.758091
H	-2.887898	5.554998	0.568311
H	-2.401704	3.726771	2.070541
H	-0.626925	4.704758	0.725658
H	-2.103316	2.622705	-3.359566
H	-0.510285	4.162529	-2.363405
H	-4.686683	3.350769	-0.769672
H	-5.159369	0.924906	-0.508872
H	-1.463225	-1.437417	0.687831
H	-1.496280	-1.619503	-1.069828
H	-5.122497	-0.735715	1.080376
H	-3.586312	-0.905546	1.944312
H	-3.647895	-1.369642	-2.331632

H	-5.163133	-1.016111	-1.482346
H	-1.365908	-3.842418	0.059793
H	-2.515712	-3.207077	2.183716
H	-3.290778	-4.631523	1.461783
H	-5.006592	-2.961539	2.196103
H	-5.488592	-4.373111	0.185401
H	-6.235467	-2.771273	0.026643
H	-5.078371	-3.421490	-2.092516
H	-3.339395	-4.908018	-1.068953
H	-2.591623	-3.680124	-2.111052
H	3.950283	-0.284372	3.410407
H	2.403039	-0.633617	2.614079
H	3.758694	-1.787579	2.497666
H	4.167798	1.803433	2.525827
H	4.105832	2.204008	0.801263
H	2.625686	1.697881	1.638583
H	5.995866	0.117947	2.138977
H	5.797773	-1.211356	0.982647
H	5.987360	0.458106	0.403645
H	5.588015	-0.219845	-3.134835
H	5.897260	-0.718079	-1.461475
H	4.886287	-1.723472	-2.522630
H	3.332591	0.599577	-3.867283
H	2.567660	-0.894478	-3.300652
H	1.968774	0.673898	-2.722878
H	4.637094	1.979234	-2.620482
H	3.438468	2.199545	-1.330158
H	5.113692	1.722854	-0.935881
H	2.037079	-3.878482	-1.625787
H	1.406021	-2.251080	-1.950254
H	3.054429	-2.701747	-2.469901
H	3.920953	-4.246629	-0.095756
H	4.937694	-2.919357	-0.685496
H	4.483464	-3.004226	1.030060
H	1.542456	-3.872545	0.592680
H	2.012362	-2.619919	1.753918
H	0.804883	-2.254097	0.508500
C	-0.860628	-0.011514	3.170841
O	-0.192286	-0.971399	3.227391
O	-1.534717	0.943095	3.120054

84

TSI (V)

C	-2.141825	-1.905077	0.585447
C	-3.229027	-1.150841	-0.198286
C	-3.141362	-1.589643	-1.677756
C	-3.338285	-3.107971	-1.808735
C	-2.236224	-3.831485	-1.019443
C	-2.317973	-3.424502	0.460449
C	-4.601751	-1.574994	0.371909
C	-4.793214	-3.095461	0.240442
C	-4.714293	-3.495785	-1.242178
C	-3.690741	-3.822800	1.026287
N	-2.998271	0.289510	-0.062950
C	-3.889120	1.184102	-0.641073
C	-3.619050	2.517738	-0.646096
C	-2.308644	2.981502	-0.048549
SI	-1.169867	3.471785	-1.452843
AL	-1.556620	1.248617	0.605473
AU	0.725688	0.442574	0.680184
P	2.841102	-0.492840	-0.033473
C	3.028429	-0.109684	-1.903477
C	1.654543	-0.305489	-2.577193
C	4.297666	0.278206	0.945536
C	4.007181	1.783990	1.108178
C	2.800373	-2.390488	0.241347
C	2.163107	-2.655515	1.620585
C	4.296828	-0.304433	2.368883
C	5.682975	0.077710	0.318370
SI	-2.505156	4.318152	1.230837
C	1.846410	-3.021956	-0.786337
C	4.165911	-3.084283	0.153509
C	3.364328	1.382566	-2.066591
C	4.086954	-0.948989	-2.629938
H	-1.750982	4.578817	-2.289792
H	-3.119257	5.567827	0.657906
H	-3.395666	3.871933	2.354436
H	-1.177367	4.710442	1.818240
H	-0.942765	2.297283	-2.365356
H	0.179962	3.933407	-0.970787
H	-4.325316	3.225236	-1.086582
H	-4.806229	0.794204	-1.089637
H	-2.187725	-1.603887	1.645653
H	-1.150638	-1.599011	0.207457
H	-5.409190	-1.052761	-0.162647
H	-4.654743	-1.265064	1.427733
H	-2.157104	-1.287408	-2.071558
H	-3.903902	-1.054630	-2.264573
H	-1.519860	-3.927691	1.028983
H	-3.750300	-3.557276	2.093918
H	-3.828330	-4.913467	0.951178
H	-5.779096	-3.371675	0.645867
H	-4.873326	-4.580891	-1.348016
H	-5.511242	-2.991248	-1.811678
H	-3.281116	-3.393549	-2.870904
H	-2.353184	-4.922803	-1.116222
H	-1.247192	-3.571527	-1.429964
H	5.017310	0.270929	2.969240
H	3.312754	-0.205669	2.846239
H	4.606880	-1.353782	2.402215
H	4.764592	2.208049	1.784844
H	4.050994	2.340045	0.168894
H	3.017540	1.948100	1.557368
H	6.438630	0.514606	0.989713
H	5.931931	-0.980867	0.183229
H	5.778945	0.581378	-0.649914
H	4.146370	-0.608156	-3.675329
H	5.084144	-0.840822	-2.188758
H	3.832118	-2.014445	-2.650184
H	1.735024	0.039199	-3.619353
H	1.321701	-1.345861	-2.596194
H	0.880543	0.295440	-2.079372
H	3.297099	1.627951	-3.137009
H	2.644861	2.019362	-1.534490
H	4.377913	1.631034	-1.735951
H	1.691070	-4.073674	-0.502496
H	0.865391	-2.529083	-0.781519
H	2.245396	-3.012036	-1.805656
H	4.017228	-4.168802	0.273356
H	4.655770	-2.921546	-0.813045
H	4.849994	-2.760692	0.945728
H	2.014572	-3.741077	1.726547
H	2.779349	-2.320254	2.457754
H	1.181327	-2.167318	1.699972
O	-1.882758	1.209894	2.864613
C	-0.790434	0.800961	3.154278
O	0.116534	0.415669	3.791136

84

INT (V)

C	-2.285703	-1.866425	1.069895
C	-2.923884	-1.249198	-0.185566
C	-2.525411	-2.105397	-1.406730
C	-2.988787	-3.559460	-1.220725
C	-2.335176	-4.151685	0.037833
C	-2.742305	-3.318883	1.263274
C	-4.459758	-1.304921	-0.021792
C	-4.926956	-2.758186	0.161917

C	-4.518624	-3.588203	-1.066289
C	-4.271827	-3.349594	1.420636
N	-2.462263	0.136591	-0.302376
C	-2.795971	0.889848	-1.420590
C	-2.455276	2.207365	-1.486287
C	-1.757093	2.823993	-0.298053
SI	-0.328482	3.956128	-0.693547
AL	-1.445258	1.204829	0.803199
AU	0.933127	0.349537	1.359645
P	2.716559	-0.468395	-0.019667
C	2.606752	0.223911	-1.797787
C	1.130950	0.201858	-2.242019
C	4.348019	0.087797	0.818153
C	4.191404	1.557377	1.258592
C	2.606177	-2.381618	-0.043280
C	2.189573	-2.866016	1.360069
C	4.539334	-0.722767	2.111579
C	5.597499	-0.060314	-0.060015
SI	-3.075818	3.774988	0.658397
C	1.466696	-2.799422	-0.985276
C	3.900489	-3.085858	-0.472126
C	3.033024	1.700481	-1.778706
C	3.471041	-0.530195	-2.817119
H	-0.788006	5.309863	-1.166007
H	-3.738072	4.782072	-0.236133
H	-4.126614	2.828152	1.161734
H	-2.493657	4.509245	1.832748
H	0.519532	3.383901	-1.788335
H	0.539828	4.178574	0.512015
H	-2.676099	2.789574	-2.382927
H	-3.317270	0.399451	-2.245147
H	-2.562741	-1.264621	1.950364
H	-1.186519	-1.816749	0.973200
H	-4.938158	-0.858413	-0.907530
H	-4.743673	-0.691471	0.847619
H	-1.433149	-2.056813	-1.533859
H	-2.974880	-1.691265	-2.321432
H	-2.267323	-3.728501	2.167812
H	-4.570720	-2.771719	2.309349
H	-4.612820	-4.386012	1.572894
H	-6.022240	-2.774901	0.272819
H	-4.865274	-4.627508	-0.951033
H	-4.996607	-3.182602	-1.972328
H	-2.694283	-4.148384	-2.103553
H	-2.652331	-5.198182	0.170997
H	-1.238828	-4.154430	-0.070246
H	5.381106	-0.279484	2.663721
H	3.656033	-0.669908	2.762512
H	4.785022	-1.773162	1.926970
H	5.083533	1.833108	1.840510
H	4.107697	2.257913	0.425177
H	3.314800	1.689547	1.909064
H	6.475624	0.230859	0.536470
H	5.754009	-1.090279	-0.398775
H	5.570789	0.593577	-0.938353
H	3.388032	-0.014939	-3.786032
H	4.530845	-0.544506	-2.539224
H	3.138423	-1.562637	-2.969342
H	1.064535	0.696847	-3.221774
H	0.721599	-0.805029	-2.349231
H	0.489554	0.757749	-1.545781
H	2.794421	2.131692	-2.761879
H	2.478302	2.276606	-1.028428
H	4.107013	1.831185	-1.612910
H	1.304566	-3.880325	-0.860029
H	0.528161	-2.292682	-0.728951
H	1.690516	-2.621175	-2.041367
H	3.707506	-4.168688	-0.512999
H	4.240682	-2.771933	-1.465085
H	4.717273	-2.928789	0.240577
H	2.022016	-3.952336	1.308453
H	2.941694	-2.681449	2.130183
H	1.248161	-2.396928	1.680245
O	-1.620614	1.234140	2.607628
C	-0.377388	0.867496	2.921966
O	0.040731	0.757947	4.061477

84

TSII (V)

C	2.662255	-3.951338	-1.414518
C	1.598444	-3.154785	-0.643568
C	2.045697	-1.691665	-0.519504
C	3.382562	-1.588773	0.229662
C	4.437497	-2.404414	-0.547904
C	3.999786	-3.874105	-0.661211
C	1.425534	-3.752574	0.761424
C	2.762198	-3.675694	1.516359
C	3.199212	-2.206224	1.632924
C	3.831431	-4.468321	0.746865
N	3.754772	-0.171759	0.319968
AL	2.950806	1.380092	-0.193050
C	4.952062	0.196699	0.937840
C	5.288662	1.507781	1.062069
C	4.340962	2.559658	0.521381
SI	5.179877	3.620293	-0.777155
SI	3.631628	3.570874	1.935909
AU	-1.327291	0.636060	-0.628902
P	-3.258128	-0.019960	0.524391
C	-2.982861	-1.804386	1.169432
C	-4.255907	-2.519898	1.639745
C	-3.525289	1.173065	2.000565
C	-4.488850	0.657255	3.077355
C	-4.773254	0.027895	-0.645284
C	-6.134097	-0.015213	0.061503
C	-2.152856	1.480036	2.634760
C	-4.046124	2.514308	1.456705
C	-4.671089	1.301729	-1.508170
C	-4.675778	-1.152872	-1.625635
C	-2.306093	-2.624564	0.052856
C	-1.972327	-1.757946	2.328090
H	4.723820	4.235743	2.723185
H	6.383664	4.310896	-0.201514
H	5.644639	2.769284	-1.922521
H	4.246104	4.669669	-1.304340
H	2.870857	2.673664	2.871273
H	2.695703	4.632701	1.436627
H	6.231673	1.796627	1.529983
H	5.604704	-0.595208	1.309334
H	2.149758	-1.241679	-1.520131
H	1.278657	-1.111926	0.021884
H	5.410566	-2.343374	-0.037261
H	4.563131	-1.958336	-1.547124
H	2.445382	-1.618378	2.181145
H	4.141560	-2.136417	2.197789
H	0.641416	-3.191771	-1.186115
H	2.776749	-3.542117	-2.430714
H	2.345379	-5.001386	-1.516977
H	4.768460	-4.438071	-1.211673
H	3.535418	-5.526928	0.674973
H	4.790057	-4.432979	1.288992
H	2.642543	-4.098045	2.526250
H	1.092254	-4.800028	0.686996
H	0.648670	-3.200233	1.312990
H	-5.444730	-1.011820	-2.399780
H	-3.699763	-1.182168	-2.127837
H	-4.863748	-2.120428	-1.149604
H	-5.470081	1.265296	-2.264148
H	-4.793487	2.225769	-0.938588

H	-3.706409	1.345514	-2.032569
H	-6.923548	-0.041638	-0.705265
H	-6.252773	-0.904686	0.690327
H	-6.311542	0.871738	0.679853
H	-4.613986	1.443852	3.837381
H	-5.481252	0.422639	2.676532
H	-4.104228	-0.230989	3.590823
H	-2.296051	2.270588	3.386841
H	-1.703009	0.620865	3.137564
H	-1.442606	1.847835	1.881224
H	-4.032127	3.240803	2.282671
H	-3.401054	2.905034	0.658584
H	-5.075246	2.455619	1.088152
H	-1.689839	-2.792977	2.571252
H	-1.057760	-1.220058	2.044074
H	-2.383004	-1.308120	3.237337
H	-3.973661	-3.506819	2.037742
H	-4.773717	-1.976349	2.437604
H	-4.963965	-2.690524	0.821366
H	-2.019783	-3.601078	0.471841
H	-2.952996	-2.807591	-0.807841
H	-1.394274	-2.125510	-0.303323
C	0.449293	1.112274	-1.552113
O	0.798277	0.855087	-2.693331
O	1.341810	1.725387	-0.695894

84

PC (V)

C	-5.137819	1.312702	1.710658
C	-5.272429	2.770427	2.179899
C	-6.016189	3.587028	1.110383
C	-5.227996	3.546262	-0.209411
C	-5.093841	2.088920	-0.680942
C	-4.356758	1.241740	0.379670
C	-3.872441	3.365840	2.398909
C	-3.086771	3.319161	1.078331
C	-3.827988	4.141391	0.012051
C	-2.966891	1.862141	0.607013
N	-4.175750	-0.141392	-0.066901
AL	-2.687516	-1.149850	-0.429585
AU	1.532243	-0.276023	-0.285118
P	3.810656	0.237065	-0.164308
C	4.799758	-1.141395	-1.052279
C	4.050750	-1.522088	-2.345547
C	-5.286014	-0.947708	-0.306587
C	-5.128659	-2.234805	-0.716771
C	-3.718651	-2.766028	-0.912246
SI	-3.454926	-3.236060	-2.702110
SI	-3.378256	-4.167712	0.277459
C	4.109146	1.923503	-1.020683
C	2.966487	2.880773	-0.625752
C	4.310933	0.333614	1.681500
C	3.618083	-0.819788	2.435355
C	5.462891	2.569048	-0.696834
C	3.991536	1.732673	-2.542390
C	5.823485	0.285223	1.932923
C	3.740872	1.630077	2.281362
C	6.250632	-0.768697	-1.383371
C	4.788616	-2.402519	-0.171859
H	-4.347287	-5.303657	0.094529
H	-4.441423	-4.277415	-3.155384
H	-3.633461	-2.038144	-3.591185
H	-2.076028	-3.787112	-2.937347
H	-3.510576	-3.694172	1.697292
H	-1.992459	-4.723051	0.100671
H	-5.998169	-2.868190	-0.904870
H	-6.279979	-0.519982	-0.156635
H	-2.394462	1.817671	-0.334707
H	-2.424518	1.264659	1.358039
H	-6.090188	1.660687	-0.870662
H	-4.530620	2.036549	-1.626390
H	-4.606376	0.708827	2.463333
H	-6.136058	0.867577	1.581041
H	-2.077709	3.732705	1.231805
H	-3.261364	4.133374	-0.932936
H	-3.910489	5.191056	0.336701
H	-5.760257	4.129368	-0.977062
H	-6.134084	4.629565	1.446921
H	-7.027291	3.176170	0.957903
H	-5.837348	2.796320	3.124852
H	-3.954485	4.406479	2.751418
H	-3.339009	2.796352	3.176696
H	4.558652	-2.393001	-2.786424
H	3.010968	-1.806210	-2.131945
H	4.040603	-0.727930	-3.095218
H	5.207755	-3.228727	-0.764840
H	5.401572	-2.301434	0.729277
H	3.767992	-2.685768	0.117445
H	6.739735	-1.646560	-1.832330
H	6.316059	0.046868	-2.111956
H	6.826106	-0.486761	-0.494568
H	5.999282	0.407591	3.012519
H	6.265508	-0.673211	1.638922
H	6.361746	1.087705	1.416584
H	3.883554	1.586405	3.371212
H	4.249553	2.529313	1.919750
H	2.663545	1.725069	2.091273
H	3.814966	-0.688163	3.509943
H	2.530266	-0.795364	2.282674
H	3.981510	-1.809369	2.149230
H	3.074717	3.800845	-1.219571
H	1.986054	2.440480	-0.855357
H	2.977742	3.162517	0.429423
H	5.552110	3.497790	-1.280689
H	5.554238	2.840490	0.360657
H	6.309531	1.927171	-0.964021
H	3.997289	2.729707	-3.007101
H	4.826804	1.167848	-2.968066
H	3.048517	1.242417	-2.818107
O	-1.205852	-0.507962	-1.417977
O	-1.139268	-1.157792	0.658112
C	-0.463083	-0.697129	-0.360032

84

PC_CO (V)

C	-2.923204	-2.051434	-1.230249
C	-3.032591	-3.538168	-0.854912
C	-4.449638	-3.831627	-0.334685
C	-4.730799	-2.969749	0.907289
C	-4.624688	-1.482868	0.532154
C	-3.211316	-1.160611	-0.001884
C	-2.006927	-3.863919	0.242326
C	-2.288516	-2.994839	1.478254
C	-3.702165	-3.294916	2.002816
C	-2.194136	-1.512626	1.094508
N	-3.070918	0.262178	-0.324560
AL	-1.983520	1.590216	0.333065
AU	0.890045	0.776825	0.791963
P	2.760196	-0.171162	-0.042529
C	4.224092	0.141472	1.157649
C	4.080518	-0.776511	2.383093
C	-3.888091	0.834369	-1.295891
C	-3.789310	2.159762	-1.585755
C	-2.780331	2.987686	-0.820428
SI	-3.669108	4.262256	0.224714
SI	-1.486701	3.727204	-1.949084
C	2.482698	-2.058508	-0.247438
C	1.520666	-2.288901	-1.425068

C	3.137594	0.619986	-1.748451
C	3.663291	2.048373	-1.524195
C	3.766352	-2.867381	-0.480372
C	1.760908	-2.593598	1.004871
C	4.142353	-0.162420	-2.605064
C	1.815673	0.777598	-2.524339
C	5.609535	-0.075039	0.534439
C	4.111603	1.582188	1.695215
H	-2.080534	4.696843	-2.934453
H	-4.527599	5.161402	-0.621362
H	-4.572031	3.581515	1.214290
H	-2.707871	5.130436	0.985520
H	-0.818010	2.644082	-2.747294
H	-0.424601	4.455674	-1.175814
H	-4.429822	2.611364	-2.346151
H	-4.604931	0.193344	-1.813606
H	-2.370779	-0.875393	1.976005
H	-1.181425	-1.273575	0.728481
H	-5.377768	-1.232822	-0.230809
H	-4.822589	-0.845361	1.408405
H	-1.913483	-1.817688	-1.604956
H	-3.632009	-1.815260	-2.038457
H	-1.546594	-3.211127	2.262835
H	-3.906312	-2.692999	2.902423
H	-3.779570	-4.355181	2.292124
H	-5.745906	-3.177692	1.279795
H	-4.543502	-4.899731	-0.081552
H	-5.191948	-3.615230	-1.119771
H	-2.830184	-4.153208	-1.745812
H	-2.065192	-4.930896	0.510584
H	-0.987361	-3.674423	-0.127960
H	4.826487	-0.459309	3.127018
H	3.090953	-0.680110	2.847142
H	4.273582	-1.829479	2.155061
H	4.905104	1.726453	2.444085
H	4.236028	2.347182	0.925253
H	3.143983	1.743408	2.188874
H	6.366851	0.065866	1.320672
H	5.736941	-1.085740	0.131085
H	5.829730	0.645385	-0.260547
H	4.337819	0.416629	-3.520407
H	5.101952	-0.317546	-2.100412
H	3.751849	-1.137384	-2.917204
H	2.033602	1.319331	-3.456954
H	1.346680	-0.171673	-2.792761
H	1.090378	1.369427	-1.952011
H	3.709237	2.546754	-2.503720
H	2.985461	2.630909	-0.886276
H	4.669798	2.074466	-1.094994
H	1.230515	-3.350099	-1.422811
H	0.604865	-1.691666	-1.321775
H	1.977195	-2.077018	-2.396952
H	3.486159	-3.918640	-0.647391
H	4.325458	-2.530935	-1.359833
H	4.436731	-2.844296	0.385655
H	1.494924	-3.645072	0.819357
H	2.373785	-2.560691	1.908195
H	0.834574	-2.037610	1.196683
O	-0.785653	1.640544	1.539173
C	0.680089	0.058752	3.881442
O	-0.181954	-0.656711	4.079568

66

Complex [$^{t}Bu_3$PAu-**VI**]

C	2.652497	-1.013847	2.621056
C	3.153640	0.221581	1.853541
C	2.423236	1.427534	2.479464
P	2.590293	0.075195	0.026125
C	3.506550	1.370450	-1.047885

C	2.698852	1.557057	-2.348601
AU	0.197225	0.439535	-0.030671
AL	-2.205870	0.524436	0.111406
C	2.956854	-1.690949	-0.627215
C	1.862897	-2.636015	-0.089370
C	4.666829	0.365002	2.058619
C	2.794178	-1.691997	-2.156345
C	4.342920	-2.241712	-0.269116
C	3.456157	2.727470	-0.326490
C	4.964723	1.030493	-1.380730
H	3.129658	2.405469	-2.901874
H	2.723392	0.684739	-3.005764
H	1.647574	1.791065	-2.127989
H	3.820503	3.495484	-1.025085
H	2.429896	2.995396	-0.041791
H	4.094246	2.763010	0.562339
H	5.397360	1.864398	-1.955321
H	5.577451	0.892808	-0.482618
H	5.052386	0.130383	-1.999430
H	2.830331	-2.736974	-2.499046
H	1.824301	-1.275546	-2.459693
H	3.593096	-1.149914	-2.672232
H	1.985178	-3.614217	-0.578881
H	1.916228	-2.795434	0.989995
H	0.859243	-2.255813	-0.327740
H	4.459100	-3.230476	-0.739835
H	5.155078	-1.603047	-0.634147
H	4.470358	-2.379321	0.810497
H	4.873367	0.391051	3.139969
H	5.228512	-0.474504	1.633557
H	5.058954	1.294288	1.630656
H	2.626773	1.428455	3.561084
H	2.748759	2.390266	2.078451
H	1.335918	1.345633	2.335904
H	2.818761	-0.835584	3.694081
H	1.575856	-1.172220	2.471796
H	3.188603	-1.930098	2.353913
C	-3.698087	1.867513	-0.017735
C	-4.968984	0.972984	-0.049309
C	-4.800539	-0.274162	0.840175
C	-3.457554	-0.994052	0.545658
SI	-3.436140	2.707881	1.635080
SI	-3.540227	3.034817	-1.453744
H	-5.154740	0.643129	-1.087425
H	-5.869624	1.529308	0.261950
H	-5.667760	-0.942762	0.704925
H	-4.819537	0.042521	1.898734
SI	-3.421800	-1.848193	-1.118171
SI	-2.812791	-2.072262	1.913571
H	-3.657851	-3.290537	2.188182
H	-1.427165	-2.560379	1.605509
H	-2.758893	-1.295399	3.201481
H	-4.770707	-2.366944	-1.529345
H	-2.989834	-0.877218	-2.199660
H	-2.423119	-2.966302	-1.152465
H	-2.209434	3.727746	-1.439912
H	-3.661689	2.276737	-2.747772
H	-4.598181	4.107782	-1.486458
H	-2.625725	3.962218	1.504768
H	-4.729036	3.012579	2.336174
H	-2.648598	1.799362	2.559293

69

RC (VI)

C	2.767743	-1.107171	-2.986837
C	2.997894	-1.673998	-1.575973
C	4.410654	-2.266723	-1.496191
C	1.950091	-2.792407	-1.396233
P	2.621777	-0.296339	-0.296430

C	3.459733	1.341918	-0.829211
C	4.910597	1.213236	-1.309781
C	3.264108	-0.829032	1.431080
C	4.781474	-0.711812	1.625407
AU	0.217986	-0.001192	-0.152001
AL	-2.179532	-0.086812	0.110916
C	-3.817808	0.615953	-0.811623
SI	-3.371985	0.854310	-2.610445
SI	-4.426944	2.231329	-0.096439
C	2.532938	0.018471	2.491583
C	2.829928	-2.281074	1.692556
C	-3.161690	-1.579008	1.030405
SI	-2.682264	-1.802607	2.813667
SI	-2.564672	-3.055650	0.041291
C	2.592707	1.970161	-1.939216
C	3.400340	2.323383	0.353528
C	-4.651037	-1.217967	0.779005
C	-4.834510	-0.550514	-0.599153
H	2.803316	-0.369086	3.485736
H	2.794932	1.078284	2.463080
H	1.443131	-0.070646	2.376138
H	3.043257	-2.512341	2.746913
H	1.750923	-2.413524	1.534565
H	3.373308	-3.008499	1.081092
H	5.036807	-1.090835	2.627369
H	5.344587	-1.301880	0.893611
H	5.129191	0.326004	1.572268
H	3.708160	3.314173	-0.013122
H	2.380789	2.415873	0.750929
H	4.077284	2.050475	1.169502
H	2.973809	2.982707	-2.141347
H	2.616212	1.412304	-2.878231
H	1.544666	2.056787	-1.618164
H	5.295697	2.219288	-1.538561
H	5.565347	0.771893	-0.549894
H	4.994307	0.617154	-2.225565
H	4.527648	-3.004963	-2.305011
H	5.190683	-1.507537	-1.624121
H	4.590499	-2.789980	-0.550546
H	2.073621	-3.512162	-2.219736
H	2.054240	-3.340601	-0.457217
H	0.928315	-2.389195	-1.444063
H	2.810794	-1.944561	-3.699310
H	1.777137	-0.642292	-3.078865
H	3.531852	-0.382330	-3.285840
H	-4.989799	-0.514337	1.557883
H	-5.310691	-2.100305	0.846694
H	-5.879229	-0.212949	-0.712813
H	-4.683724	-1.319281	-1.378914
H	-4.577986	1.103073	-3.477494
H	-2.435398	2.013295	-2.789003
H	-2.697519	-0.376625	-3.153212
H	-5.591601	2.779611	-0.878834
H	-4.895864	2.078506	1.321987
H	-3.344376	3.271814	-0.111364
H	-1.189262	-1.753274	2.978451
H	-3.284706	-0.715368	3.658944
H	-3.136758	-3.110685	3.410880
H	-1.298207	-3.610369	0.625598
H	-3.598080	-4.147423	-0.008167
H	-2.246572	-2.697420	-1.390142
C	-1.004564	2.260748	2.248289
O	0.015989	2.826842	2.251313
O	-2.039961	1.707015	2.263978

69

RC (VI)

C	2.439732	2.402303	-1.452073
C	2.873535	0.942756	-1.669871
C	4.295421	0.918131	-2.245474
C	1.881657	0.361026	-2.698521
P	2.652692	-0.013518	-0.022622
C	3.382146	1.007333	1.427321
C	4.755754	1.633546	1.155256
C	3.530019	-1.713753	-0.130195
C	5.056789	-1.669788	0.010256
AU	0.267433	-0.306792	0.350698
AL	-2.154496	-0.118750	0.359115
SI	-3.895589	1.692049	2.391954
C	-3.154092	1.593096	0.682349
SI	-1.934268	2.983304	0.424542
C	2.927124	-2.623720	0.958878
C	3.164807	-2.369535	-1.472252
C	-3.497670	-0.870489	-0.922575
SI	-3.872385	-2.671398	-0.631739
SI	-2.695549	-0.662821	-2.603504
C	2.367752	2.110776	1.791264
C	3.476041	0.099432	2.665105
C	-4.706537	0.084137	-0.696796
C	-4.239328	1.532232	-0.439101
H	3.321806	-3.640782	0.813269
H	3.176496	-2.312160	1.975728
H	1.832159	-2.667437	0.873311
H	3.520024	-3.410664	-1.447776
H	2.078012	-2.391582	-1.628676
H	3.638679	-1.880777	-2.329490
H	5.450862	-2.689755	-0.120622
H	5.527770	-1.032349	-0.746675
H	5.375303	-1.323279	0.999725
H	3.726669	0.731912	3.529892
H	2.518137	-0.393378	2.879185
H	4.257349	-0.662440	2.578901
H	2.713043	2.603476	2.713002
H	2.264038	2.879130	1.021777
H	1.372051	1.686066	1.981850
H	5.093740	2.147404	2.068691
H	5.513452	0.885703	0.895298
H	4.721007	2.382280	0.356055
H	4.314704	1.535329	-3.157294
H	5.036099	1.329643	-1.550577
H	4.612905	-0.091351	-2.529766
H	1.904978	1.000801	-3.593902
H	2.123360	-0.657417	-3.010997
H	0.855189	0.365672	-2.304506
H	2.404755	2.893577	-2.435920
H	1.434598	2.464803	-1.013620
H	3.139104	2.967892	-0.828068
H	-5.282031	-0.257673	0.180114
H	-5.404898	0.073206	-1.551258
H	-5.111777	2.164477	-0.200320
H	-3.826202	1.936351	-1.380564
H	-2.599015	4.334378	0.355502
H	-0.920783	3.039235	1.529878
H	-1.192816	2.808749	-0.873543
H	-4.650498	2.978509	2.600020
H	-4.867989	0.576158	2.650977
H	-2.827163	1.614876	3.443978
H	-2.605283	-3.461953	-0.467201
H	-4.692482	-2.835529	0.617049
H	-4.646750	-3.318172	-1.750915
H	-1.751048	-1.796407	-2.878112
H	-3.722074	-0.614933	-3.702828
H	-1.888918	0.605168	-2.709989
O	-2.489093	-1.341026	2.209246
C	-1.358722	-1.665642	2.444879
O	-0.407637	-2.141810	2.930840

69

INT (VI)

C	2.287824	2.077668	-2.158333
C	2.508317	0.567933	-1.972268
P	2.506128	0.157419	-0.106902
AU	0.334208	0.305336	0.924842
AL	-2.207222	0.126419	0.473230
O	-2.375321	0.479321	2.265835
C	-1.079437	0.564043	2.478101
O	-0.532128	0.789782	3.544192
C	3.794021	0.156308	-2.702223
C	1.294749	-0.131528	-2.610257
C	3.565001	1.433972	0.857634
C	3.792461	0.921695	2.290065
C	3.171674	-1.613972	0.192547
C	2.532965	-2.571699	-0.826021
C	4.921898	1.741129	0.210339
C	2.749734	2.735434	1.000475
C	4.699061	-1.735027	0.107692
C	2.686637	-2.083260	1.579210
C	-3.004569	1.373525	-0.844889
C	-3.357417	0.350616	-1.967206
C	-3.896226	-0.975010	-1.380661
C	-2.954522	-1.555073	-0.274383
SI	-1.661513	-2.698112	-0.982663
SI	-4.534788	2.096194	-0.018970
SI	-1.914492	2.777462	-1.404219
SI	-3.899969	-2.466537	1.061616
H	3.009920	-3.126141	1.715985
H	3.090842	-1.497774	2.407932
H	1.589548	-2.062556	1.644800
H	2.794560	-3.597959	-0.528462
H	1.438733	-2.498120	-0.822512
H	2.900215	-2.422391	-1.846059
H	4.967496	-2.794818	0.234289
H	5.093462	-1.407114	-0.860543
H	5.208969	-1.174459	0.898417
H	4.260339	1.734909	2.864239
H	2.847789	0.669783	2.791141
H	4.464289	0.059075	2.336625
H	3.326559	3.427709	1.631703
H	2.551310	3.236515	0.050789
H	1.788069	2.552997	1.501352
H	5.466653	2.437570	0.865768
H	5.542506	0.846742	0.088856
H	4.818877	2.227290	-0.765736
H	3.709049	0.475306	-3.752133
H	4.686181	0.633699	-2.282260
H	3.948076	-0.927943	-2.703741
H	1.219923	0.198494	-3.657101
H	1.369676	-1.220645	-2.607680
H	0.363650	0.150262	-2.102112
H	2.120325	2.259982	-3.230120
H	1.397111	2.430112	-1.623947
H	3.152313	2.675967	-1.854029
H	-4.895410	-0.781374	-0.952199
H	-4.055312	-1.697028	-2.198933
H	-4.100577	0.760656	-2.671499
H	-2.453880	0.129652	-2.561172
H	-2.667480	3.858022	-2.134814
H	-1.215899	3.432415	-0.248171
H	-0.870928	2.262810	-2.353475
H	-5.548155	2.483983	-1.059286
H	-5.200010	1.129086	0.919009
H	-4.159687	3.311009	0.775215
H	-2.960237	-2.995980	2.103782
H	-4.890434	-1.552961	1.721734
H	-4.668599	-3.630581	0.497758
H	-0.658814	-3.090938	0.062923
H	-2.274601	-3.961826	-1.524401
H	-0.910355	-2.067730	-2.118781

69

TSII (VI)

C	-3.254692	-2.896793	0.767566
C	-3.010482	-2.225700	-0.594482
C	-4.072308	-2.707031	-1.591759
C	-1.614984	-2.698078	-1.052430
P	-2.954585	-0.327018	-0.338381
C	-4.416884	0.234836	0.762613
C	-5.771696	-0.387798	0.401028
C	-3.025794	0.563522	-2.034103
C	-4.420817	0.609918	-2.670835
AU	-0.925539	0.201620	0.702778
C	0.935447	0.629096	1.464557
O	1.806377	-0.446473	1.347833
AL	3.249386	-0.349106	0.393695
C	4.607134	-1.739459	0.141827
SI	6.038153	-1.507740	1.336413
C	-2.485858	1.996332	-1.851628
C	-2.053395	-0.130375	-3.003164
O	1.367122	1.691037	1.886175
C	-4.065475	-0.091819	2.227984
C	-4.526909	1.767481	0.689662
C	3.651382	0.815813	-1.132910
SI	2.246910	0.587855	-2.360230
SI	3.899770	2.628410	-0.721409
C	4.984482	0.140422	-1.597595
C	5.004314	-1.385017	-1.331542
SI	3.869553	-3.454684	0.295698
H	-2.420748	2.463853	-2.845730
H	-3.122659	2.627305	-1.227281
H	-1.478109	1.984343	-1.413853
H	-1.976387	0.495076	-3.904959
H	-1.047212	-0.219223	-2.572981
H	-2.397441	-1.120429	-3.318789
H	-4.335795	1.088200	-3.658696
H	-4.847595	-0.388072	-2.821029
H	-5.129034	1.203595	-2.082494
H	-5.246847	2.089780	1.456632
H	-3.566765	2.252413	0.910139
H	-4.895249	2.125337	-0.277056
H	-4.844958	0.345152	2.870412
H	-4.021793	-1.162776	2.438297
H	-3.101398	0.353991	2.509995
H	-6.540552	0.045993	1.058763
H	-6.065938	-0.181720	-0.634064
H	-5.786460	-1.472291	0.556104
H	-4.051503	-3.807367	-1.617698
H	-5.085527	-2.401330	-1.307996
H	-3.877388	-2.354749	-2.610722
H	-1.618390	-3.798497	-1.068812
H	-1.343282	-2.351673	-2.052115
H	-0.835673	-2.367676	-0.351478
H	-3.110821	-3.980140	0.640423
H	-2.535790	-2.551662	1.522589
H	-4.270997	-2.742552	1.144046
H	5.831027	0.596530	-1.056739
H	5.169526	0.322199	-2.668539
H	6.001761	-1.782436	-1.580517
H	4.298546	-1.868590	-2.029286
H	4.848915	-4.523948	-0.101425
H	3.423870	-3.721757	1.700183
H	2.683076	-3.570823	-0.620161
H	7.221216	-2.327457	0.905722
H	6.482766	-0.073210	1.391937
H	5.632572	-1.916101	2.719198
H	2.607632	3.293541	-0.373671
H	4.858027	2.760594	0.427364

H	4.511314	3.353003	-1.889839
H	1.083713	1.461025	-2.005321
H	2.696315	0.903609	-3.759036
H	1.738245	-0.831202	-2.372999

69

PC (VI)

C	3.783190	1.181566	-2.571237
C	3.992749	-0.163974	-1.856388
C	3.098475	-1.178350	-2.596847
P	3.364758	-0.007082	-0.053957
C	3.937515	-1.514893	0.978508
C	3.037237	-1.618389	2.225648
C	5.464265	-0.580406	-1.983779
AU	1.024092	0.059291	-0.077508
C	-1.018418	0.114394	-0.120015
O	-1.771328	0.184069	0.947595
C	4.008611	1.616750	0.730945
C	3.152929	2.784736	0.199885
AL	-3.286495	0.109571	-0.180712
C	-4.536557	-1.425569	-0.206022
SI	-4.197793	-2.642374	1.167663
C	-4.638668	1.551619	-0.278278
SI	-4.209967	2.895159	-1.500100
C	3.760176	1.567875	2.247823
C	5.493361	1.902917	0.471211
C	3.686156	-2.798316	0.168951
C	5.411827	-1.469280	1.401797
C	-5.883544	0.709123	-0.709055
C	-5.898482	-0.678956	-0.029679
SI	-4.846380	2.277121	1.439042
SI	-4.404359	-2.259794	-1.879024
H	3.295946	-2.548260	2.754102
H	3.164361	-0.790418	2.926620
H	1.976439	-1.669892	1.943919
H	3.858128	-3.654957	0.837296
H	2.649248	-2.858087	-0.187341
H	4.364488	-2.906586	-0.683188
H	5.648150	-2.403498	1.933589
H	6.092642	-1.393839	0.546752
H	5.624637	-0.642401	2.088048
H	3.975170	2.566836	2.655152
H	2.713175	1.332575	2.479969
H	4.410437	0.855679	2.765610
H	3.450180	3.695492	0.741238
H	3.285176	2.973515	-0.867745
H	2.084559	2.610015	0.389089
H	5.768356	2.821464	1.011802
H	6.146149	1.099431	0.829946
H	5.706379	2.075162	-0.589483
H	5.733460	-0.579150	-3.051023
H	6.143514	0.108860	-1.470193
H	5.645903	-1.592058	-1.604585
H	3.386504	-1.171302	-3.658841
H	3.203167	-2.202083	-2.231074
H	2.039043	-0.894618	-2.530046
H	3.983574	1.028189	-3.641900
H	2.747728	1.534161	-2.474669
H	4.462451	1.964290	-2.219088
H	-6.104366	-0.538004	1.045694
H	-6.740005	-1.271447	-0.426095
H	-6.825344	1.236456	-0.482470
H	-5.867258	0.563222	-1.802828
H	-5.240005	3.992130	-1.553882
H	-2.895337	3.531569	-1.156680
H	-4.112435	2.315736	-2.883465
H	-6.193621	2.928287	1.584880
H	-4.743596	1.232335	2.517035
H	-3.782215	3.298142	1.711591
H	-2.815787	-3.214704	1.051387
H	-4.318321	-1.955868	2.498939
H	-5.164231	-3.797192	1.180605
H	-3.163133	-3.097434	-1.956898
H	-5.595298	-3.140808	-2.140000
H	-4.343748	-1.263522	-3.004337
O	-1.710041	0.067107	-1.226911

69

PC_CO (VI)

C	3.265807	1.535342	-2.524756
C	3.107420	0.088102	-2.028237
C	1.875544	-0.475206	-2.763316
P	2.737547	0.117867	-0.145556
C	2.929404	-1.645016	0.580944
C	2.196648	-1.698752	1.935339
C	4.350869	-0.720492	-2.421853
AU	0.631676	0.857059	0.152797
O	-1.228456	1.604081	0.394741
C	3.955325	1.313573	0.732669
C	3.468805	2.759125	0.507926
AL	-2.627691	0.757485	-0.021865
C	-3.002333	-1.149227	-0.430904
SI	-2.313890	-2.329608	0.837329
C	-4.470389	1.441121	-0.264512
SI	-4.531623	3.023231	-1.251832
C	3.884924	1.080177	2.251269
C	5.414134	1.182313	0.273900
C	2.198419	-2.641897	-0.334209
C	4.383322	-2.102240	0.763993
C	-5.105346	0.223493	-1.003089
C	-4.560299	-1.124421	-0.472748
SI	-5.180308	1.677250	1.456111
SI	-2.231557	-1.465227	-2.112040
H	2.239250	-2.735541	2.302252
H	2.639645	-1.055350	2.698718
H	1.140908	-1.423659	1.818370
H	2.159845	-3.610557	0.186071
H	1.164498	-2.328642	-0.528594
H	2.711350	-2.801965	-1.287986
H	4.373962	-3.144021	1.119318
H	4.955719	-2.076975	-0.169617
H	4.917129	-1.508981	1.514326
H	4.459611	1.881796	2.739078
H	2.854048	1.138087	2.622637
H	4.328115	0.127837	2.559345
H	4.121451	3.429678	1.087317
H	3.507173	3.076644	-0.536659
H	2.439629	2.887589	0.869145
H	6.029313	1.860415	0.884980
H	5.808820	0.169012	0.406570
H	5.552957	1.474221	-0.772707
H	4.506134	-0.608639	-3.505879
H	5.259971	-0.372277	-1.919878
H	4.231895	-1.790777	-2.220423
H	2.059079	-0.390451	-3.845184
H	1.674737	-1.525099	-2.539415
H	0.973861	0.102613	-2.521080
H	3.300442	1.511369	-3.624048
H	2.409524	2.156883	-2.231011
H	4.188471	2.011169	-2.177885
H	-4.955132	-1.283277	0.545731
H	-4.960656	-1.944748	-1.090672
H	-6.204458	0.228255	-0.916155
H	-4.881470	0.289563	-2.082782
H	-5.938776	3.498408	-1.500057
H	-3.793422	4.123837	-0.553742
H	-3.904252	2.800267	-2.600338
H	-6.677412	1.539710	1.448936

H	-4.653912	0.648702	2.423872
H	-4.809030	3.021666	2.003894
H	-0.837091	-2.137552	1.000162
H	-2.974679	-2.096037	2.166634
H	-2.536829	-3.775899	0.480033
H	-0.837502	-1.996039	-1.964227
H	-3.047339	-2.449540	-2.902196
H	-2.127240	-0.212350	-2.944464
C	0.208655	1.109749	3.318845
O	-0.681563	0.483204	3.648980

94

Complex [^{t}Bu$_3$PAu-**I**^{C}]

C	0.984531359	0.034307772	-0.374843473
AU	-1.116044238	0.034927725	-0.208731739
C	2.948516442	1.567183624	0.283328038
C	3.683950535	0.714032041	1.089539685
C	5.071370525	0.788119842	1.236151017
C	5.682434550	1.962897030	0.795993103
C	4.918831682	2.946543756	0.149962663
C	3.572865465	2.743800533	-0.155697095
C	5.761748436	-0.557904051	1.567887149
C	5.043516126	-1.521041480	0.590763352
C	3.657912227	-1.355847592	0.519044612
O	3.072486294	-0.486345014	1.432064362
C	2.902109556	-1.665023746	-0.599130756
C	3.499465536	-2.455956150	-1.590680842
C	4.842952649	-2.805221116	-1.453390759
C	5.628314375	-2.308499905	-0.401765470
N	1.553901371	-1.162438324	-0.701584652
C	0.649629271	-2.157629178	-1.252288294
P	-3.409332735	0.020763633	0.200335848
C	-3.715976312	0.648334934	1.983708322
C	-2.769209889	1.832068009	2.254963385
C	7.262265887	-0.495350700	1.285697888
C	5.544058489	-0.970014662	3.041918328
N	1.594072470	1.217900472	-0.069045469
C	0.723300380	2.382316402	-0.069663495
C	-4.252992743	1.183979654	-1.065230003
C	-3.585336880	0.968771582	-2.437967936
C	-4.085170419	-1.759611812	0.005975896
C	-3.089859665	-2.733748904	0.664366598
C	-5.771080921	0.991447030	-1.185959203
C	-3.963033418	2.641893729	-0.676324215
C	-5.484461340	-1.970455521	0.600455332
C	-4.110952743	-2.118791383	-1.488726554
C	-5.166314646	1.068886632	2.259989119
C	-3.312222367	-0.453113512	2.978207955
H	3.024025876	3.474994460	-0.746515298
H	6.758952040	2.097708874	0.892350066
H	6.701936860	-2.491784167	-0.403359639
H	2.931714053	-2.770627928	-2.464707786
H	7.724044688	-1.472543570	1.476278871
H	7.741481540	0.231813324	1.953543211
H	7.474603717	-0.206791095	0.248170573
H	6.008356918	-1.948471656	3.221667992
H	4.482257901	-1.034217276	3.298707120
H	6.020689862	-0.234545400	3.703139892
H	5.403899077	3.861477610	-0.188698250
H	5.308676657	-3.420140386	-2.222930008
H	-4.307210925	3.283038105	-1.501467356
H	-4.494752250	2.958118332	0.226420264
H	-2.889677999	2.821970228	-0.543370883
H	-4.011276827	1.700278939	-3.140865461
H	-2.501978826	1.144552780	-2.378306327
H	-3.749348226	-0.028249867	-2.852975271
H	-6.158083987	1.748893632	-1.884099034
H	-6.041493309	0.010183656	-1.589958780
H	-6.288709802	1.128617916	-0.230310040
H	-2.894983681	2.132670596	3.305970178
H	-1.721663954	1.540020549	2.105064483
H	-2.966450571	2.706283389	1.631022386
H	-3.328464014	-0.015404921	3.987048362
H	-4.001814837	-1.302804561	2.979122784
H	-2.293439224	-0.816961283	2.789519624
H	-5.245896900	1.349507815	3.320966973
H	-5.470422715	1.940573676	1.670764935
H	-5.882971617	0.260929553	2.078232281
H	-3.415305782	-3.758559097	0.431110949
H	-2.078917160	-2.601981281	0.259354288
H	-3.042819646	-2.638959962	1.751417597
H	-5.798791763	-3.001380348	0.378548396
H	-5.498513106	-1.855806270	1.689633509
H	-6.233594188	-1.297268221	0.170475427
H	-4.336578783	-3.192166650	-1.571339824
H	-4.881422200	-1.578179040	-2.046872130
H	-3.135148093	-1.951877790	-1.961491366
C	0.492735573	-3.375492069	-0.590528496
C	-0.310526004	-4.362915892	-1.158199101
C	-0.942167893	-4.137626514	-2.382876383
C	-0.772660434	-2.919034648	-3.041812653
C	0.029228882	-1.924552555	-2.481422283
H	0.998499280	-3.544694081	0.359814304
H	-0.444273428	-5.311653717	-0.639175141
H	-1.564718101	-4.914635832	-2.825887004
H	-1.254371129	-2.743156796	-4.003545368
H	0.191642850	-0.977579976	-2.994961198
C	0.192151478	2.842555486	-1.276173436
C	-0.568079978	4.011973972	-1.286543578
C	-0.790392119	4.713472331	-0.100263817
C	-0.249912021	4.246330001	1.099765629
C	0.517885055	3.083082604	1.118196770
H	0.390887274	2.292323219	-2.195430222
H	-0.980994620	4.377740768	-2.226385981
H	-1.382517998	5.628311932	-0.111043273
H	-0.424364433	4.790835373	2.027438511
H	0.957699335	2.717580488	2.045935689

85

Complex [^{t}Bu$_3$PAu-**II**^{C}]

C	0.0000	0.0000	2.0825
AU	0.0000	0.0000	0.0000
C	2.8955	0.9833	1.4510
C	2.3758	-0.2030	1.9787
C	3.0612	-1.4081	1.8126
C	4.2719	-1.4243	1.1178
C	4.7930	-0.2437	0.5883
C	4.1012	0.9586	0.7526
N	1.1595	-0.1669	2.7562
SI	1.4351	-0.3372	4.5482
C	1.6160	-2.1388	4.9800
P	-0.0223	-0.0528	-2.3329
C	-0.5044	1.6867	-2.9735
C	-2.0075	1.9094	-2.7366
N	-1.1658	0.1229	2.7568
SI	-1.4750	0.3159	4.5425
C	-2.5052	-1.1032	5.1598
C	-2.3653	0.2800	1.9629
C	-2.6699	1.5210	1.3957
C	-3.8465	1.6757	0.6639
C	-4.7208	0.5980	0.5057
C	-4.4148	-0.6373	1.0773
C	-3.2375	-0.7993	1.8090
C	-2.2428	1.9819	4.8330
C	1.7222	-0.5296	-2.9601
C	1.7672	-0.9666	-4.4299
C	-1.3089	-1.3511	-2.9012
C	-2.5582	-1.2213	-2.0087

C	2.2695	-1.6567	-2.0629
C	2.6659	0.6675	-2.7593
C	2.8863	0.7149	5.0269
O	0.0420	0.2725	5.1952
C	-0.7438	-2.7570	-2.6364
C	-1.7097	-1.2366	-4.3777
C	-0.1896	1.9213	-4.4569
C	0.2216	2.7389	-2.1116
H	4.5057	1.8835	0.3414
H	5.7382	-0.2589	0.0459
H	4.8026	-2.3667	0.9833
H	2.7403	1.7553	4.7076
H	2.9974	0.7009	6.1206
H	3.8178	0.3396	4.5840
H	-2.5809	-1.0337	6.2547
H	-3.5224	-1.0755	4.7487
H	-3.2467	2.0453	4.3937
H	-2.3332	2.1512	5.9156
H	-1.6217	2.7828	4.4106
H	-5.0909	-1.4826	0.9500
H	-5.6416	0.7227	-0.0638
H	-4.0840	2.6429	0.2207
H	2.3533	1.9168	1.6002
H	2.6384	-2.3294	2.2116
H	-2.9828	-1.7627	2.2498
H	-1.9829	2.3557	1.5340
H	3.6900	0.3240	-2.9674
H	2.6445	1.0297	-1.7235
H	2.4546	1.4986	-3.4390
H	3.3180	-1.8345	-2.3450
H	1.7312	-2.6009	-2.1714
H	2.2513	-1.3650	-1.0049
H	2.8164	-1.1602	-4.6997
H	1.3874	-0.1975	-5.1112
H	1.2110	-1.8934	-4.6079
H	-1.5547	-3.4811	-2.8032
H	-0.4052	-2.8690	-1.5977
H	0.0763	-3.0231	-3.3106
H	-2.4009	-2.0600	-4.6129
H	-0.8536	-1.3200	-5.0557
H	-2.2351	-0.3006	-4.5963
H	-3.2486	-2.0373	-2.2701
H	-3.0945	-0.2787	-2.1368
H	-2.2998	-1.3208	-0.9463
H	-0.1369	3.7335	-2.4161
H	1.3076	2.7260	-2.2287
H	-0.0120	2.6033	-1.0461
H	-2.2266	2.9663	-2.9488
H	-2.2883	1.7127	-1.6940
H	-2.6382	1.3063	-3.3975
H	-0.5618	2.9187	-4.7357
H	-0.6800	1.1915	-5.1104
H	0.8854	1.9064	-4.6660
H	1.5833	-2.2492	6.0737
H	2.5723	-2.5478	4.6291
H	0.8006	-2.7366	4.5505
H	-2.0508	-2.0711	4.9092

90

Complex [^{t}Bu$_3$PAu-**III**^{C}]

C	0.0000	0.0000	2.0785
AU	0.0000	0.0000	0.0000
C	3.2507	-0.4204	2.0198
C	2.0694	-1.1607	2.0109
C	2.0030	-2.3751	1.3214
C	3.1290	-2.8536	0.6542
C	4.3173	-2.1208	0.6689
C	4.3742	-0.9015	1.3444
N	0.9314	-0.6895	2.7762
SI	0.6819	-1.5950	4.3710
C	-0.9338	-2.5238	4.1700
N	-0.9532	0.6865	2.7585
SI	-0.7291	1.6402	4.3239
C	0.8599	2.6066	4.0854
P	-0.0779	-0.0623	-2.3349
C	-2.1118	1.0601	1.9715
C	-2.1689	2.2968	1.3248
C	-3.3056	2.6465	0.5978
C	-4.3839	1.7636	0.5135
C	-4.3212	0.5263	1.1557
C	-3.1863	0.1735	1.8873
C	0.6163	-0.4036	5.8173
C	-0.6199	0.5025	5.8116
C	2.1324	-2.7541	4.5862
C	-2.2216	2.7487	4.5238
H	3.0773	-3.8019	0.1192
H	5.1977	-2.4983	0.1492
H	5.2971	-0.3218	1.3522
H	2.1618	-3.5452	3.8278
H	2.0315	-3.2289	5.5738
H	3.0906	-2.2185	4.5638
H	-1.7795	-1.8458	4.0024
H	-1.1403	-3.1038	5.0809
H	-0.8787	-3.2248	3.3262
H	0.6463	-1.0256	6.7279
H	1.5437	0.1905	5.8278
H	-0.6287	1.1626	6.6957
H	-1.5459	-0.0909	5.8708
H	1.7097	1.9451	3.8744
H	1.0908	3.1773	4.9962
H	0.7624	3.3162	3.2527
H	-2.2790	3.5377	3.7653
H	-2.1451	3.2281	5.5114
H	-3.1578	2.1753	4.4980
H	-3.3459	3.6107	0.0910
H	-5.2720	2.0396	-0.0545
H	-5.1580	-0.1688	1.0888
H	1.0712	-2.9414	1.3192
H	3.2881	0.5258	2.5595
H	-1.3201	2.9777	1.3892
H	-3.1282	-0.7897	2.3936
C	-0.1004	1.7136	-3.0511
C	-1.6885	-0.9878	-2.8091
C	1.4520	-1.0076	-2.9873
C	1.2902	-2.4994	-2.6502
C	1.6957	-0.8576	-4.4945
C	2.6894	-0.5196	-2.2081
C	-1.8673	-2.1798	-1.8471
C	-2.8886	-0.0609	-2.5538
C	-1.7284	-1.4843	-4.2601
C	-0.5731	1.7955	-4.5087
C	-1.0028	2.5862	-2.1597
C	1.3091	2.3191	-2.9418
H	2.2431	-2.9997	-2.8769
H	1.0805	-2.6525	-1.5840
H	0.5129	-2.9894	-3.2450
H	3.5324	-1.1788	-2.4630
H	2.9814	0.5030	-2.4563
H	2.5302	-0.5836	-1.1241
H	2.5635	-1.4774	-4.7664
H	0.8454	-1.1979	-5.0955
H	1.9320	0.1730	-4.7799
H	1.2331	3.3888	-3.1864
H	1.7085	2.2384	-1.9222
H	2.0223	1.8739	-3.6422
H	-0.4846	2.8405	-4.8423
H	0.0318	1.1809	-5.1840
H	-1.6231	1.5063	-4.6244

H	-0.9555	3.6192	-2.5358
H	-2.0502	2.2775	-2.1575
H	-0.6478	2.5814	-1.1212
H	-2.8365	-2.6509	-2.0695
H	-1.0939	-2.9446	-1.9489
H	-1.8849	-1.8413	-0.8015
H	-3.8040	-0.6600	-2.6693
H	-2.8830	0.3397	-1.5317
H	-2.9498	0.7688	-3.2650
H	-2.7127	-1.9417	-4.4419
H	-1.6012	-0.6745	-4.9866
H	-0.9725	-2.2513	-4.4605

77

Complex [^{t}Bu$_3$PAu-**IV**^{C}]

C	-2.069616378	0.293216099	0.104967239
AU	-0.020784127	0.025076762	-0.019176065
C	-2.092586310	-2.672813156	-1.125062731
C	-2.348352764	-2.080097778	0.110277090
C	-2.121549307	-2.761985346	1.304029538
C	-1.618001306	-4.061790168	1.256854163
C	-1.355469192	-4.667282199	0.026591418
C	-1.586485252	-3.972301348	-1.161622013
N	-2.914783163	-0.749158321	0.153555653
P	2.288922578	-0.213481069	-0.185547654
C	2.703779406	-2.043478956	-0.554192888
C	1.816417047	-2.932745896	0.336968420
C	-4.345912186	-0.644075246	0.232795502
C	-4.816347403	0.719111526	0.284096561
C	-3.990926205	1.783094831	0.236166500
N	-2.593913302	1.539556344	0.135584091
C	-1.678257513	2.659316127	0.067860719
C	-1.110139713	3.151308044	1.241726655
C	-0.236608773	4.236445585	1.172264912
C	0.054817309	4.822925888	-0.060377919
C	-0.523730351	4.323873714	-1.229113489
C	-1.395181941	3.236741437	-1.169271375
C	3.083706345	0.331967225	1.467701520
C	2.840439735	-0.759261197	2.523890103
C	2.885992288	0.909232197	-1.616175867
C	4.301353682	0.587584300	-2.113870320
C	4.589196589	0.614109359	1.371821540
C	2.347216640	1.586813171	1.975008140
C	1.882917774	0.790862128	-2.781189776
C	2.828708682	2.376300339	-1.159682333
C	2.298198522	-2.359705700	-2.003663217
C	4.179833969	-2.408646671	-0.348454188
H	0.216583447	4.622852198	2.084711476
H	0.736498181	5.671714994	-0.111006358
H	-0.293888976	4.779006928	-2.192150584
H	-1.350241401	2.682850260	2.195998218
H	-1.852083654	2.832741400	-2.072529247
H	2.389450187	-3.446428297	-2.146287223
H	1.252554943	-2.086804700	-2.199860848
H	2.942087915	-1.877027460	-2.745365412
H	1.957638851	-3.976663609	0.019969169
H	2.062957682	-2.872260622	1.398941187
H	0.754618310	-2.686234919	0.212320852
H	4.318056556	-3.459569589	-0.643719805
H	4.855774459	-1.801843835	-0.960295427
H	4.488676530	-2.323148486	0.699112723
H	3.149428415	-0.355466731	3.499216578
H	1.777784778	-1.026632177	2.596041790
H	3.426618351	-1.665886573	2.344828786
H	4.960236242	0.851660214	2.380041385
H	5.156808454	-0.248551415	1.006315497
H	4.814922172	1.473850367	0.731986627
H	2.758200154	1.843863271	2.962663606
H	2.464046645	2.457595434	1.326498515
H	1.272462433	1.392222228	2.092004319
H	2.191323025	1.498333604	-3.565395692
H	1.843913287	-0.204907564	-3.227961593
H	0.869295126	1.064989395	-2.456459181
H	3.012647775	3.007929833	-2.041215549
H	1.839731531	2.640964239	-0.764810197
H	3.591764723	2.624037279	-0.415257427
H	4.570986167	1.324527144	-2.885173297
H	5.052219115	0.651474552	-1.318791304
H	4.367887191	-0.403518060	-2.575309445
H	-5.889405877	0.880192737	0.362508704
C	-4.445768047	3.201930757	0.288642543
H	-2.341355959	-2.276815078	2.254612101
H	-1.431375438	-4.601487703	2.184824469
H	-0.965096382	-5.684303379	-0.005714075
H	-1.376400250	-4.442356984	-2.122040615
H	-2.292078463	-2.119342616	-2.042267092
C	-5.145333894	-1.730403110	0.255041842
H	-6.221218631	-1.585601139	0.315313480
H	-4.762230533	-2.744907686	0.213987924
H	-5.534822407	3.232092321	0.394547491
H	-3.992183234	3.736141288	1.135232669
H	-4.164101803	3.747698048	-0.622728068

81

Complex [^{t}Bu$_3$PAu-**V**^{C}]

C	0.0000	0.0000	2.0545
AU	0.0000	0.0000	0.0000
C	-5.1581	0.5195	3.8990
C	-4.4481	1.7061	3.2275
C	-4.8037	1.7447	1.7344
C	-4.3558	0.4311	1.0775
C	-5.0754	-0.7545	1.7352
C	-4.7184	-0.7920	3.2278
C	-2.9267	1.5491	3.3881
C	-2.4724	0.2283	2.7315
C	-2.8355	0.2666	1.2456
C	-3.1985	-0.9641	3.3883
N	-0.9963	0.0723	2.9532
C	-0.5043	-0.0012	4.2761
C	0.8387	-0.1250	4.2511
C	1.2495	-0.1269	2.8316
SI	2.1872	-1.7293	2.3201
H	3.3440	-1.8645	3.2537
SI	2.3840	1.3828	2.4345
H	3.5543	1.2892	3.3560
H	1.5897	2.6192	2.6912
H	2.8181	1.3070	1.0106
H	1.2434	-2.8765	2.4579
H	2.6557	-1.5868	0.9108
H	1.4977	-0.2030	5.1105
H	-1.1825	0.0455	5.1194
H	-2.3161	1.1032	0.7544
H	-2.5038	-0.6636	0.7579
H	-2.6647	1.5552	4.4558
H	-2.3961	2.3882	2.9123
H	-2.8597	-1.8978	2.9137
H	-2.9473	-1.0265	4.4566
H	-4.5834	0.4560	0.0010
H	-4.3106	2.6011	1.2480
H	-5.8886	1.8773	1.6073
H	-4.7552	2.6455	3.7096
H	-6.2485	0.6369	3.8099
H	-4.9201	0.4940	4.9739
H	-5.2185	-1.6437	3.7113
H	-6.1635	-0.6515	1.6089
H	-4.7778	-1.6974	1.2496
P	0.0648	-0.0530	-2.3299
C	-1.1589	-1.4096	-2.9089

C	1.8407	-0.4646	-2.9135
C	-0.4753	1.6583	-2.9982
C	-0.8845	1.6490	-4.4771
C	0.6709	2.6639	-2.7971
C	-1.6437	2.1756	-2.1362
C	-0.9755	-1.8354	-4.3718
C	-2.5998	-0.9102	-2.7064
C	-1.0011	-2.6358	-1.9872
C	2.0930	-0.1777	-4.3997
C	2.1304	-1.9479	-2.6264
C	2.8375	0.3356	-2.0527
H	-1.9145	3.1781	-2.4989
H	-1.3413	2.2681	-1.0840
H	-2.5384	1.5510	-2.1857
H	0.2812	3.6658	-3.0296
H	1.5222	2.4831	-3.4604
H	1.0224	2.6776	-1.7569
H	-1.1093	2.6829	-4.7791
H	-1.7861	1.0546	-4.6594
H	-0.0877	1.2799	-5.1316
H	3.1152	-0.5029	-4.6444
H	2.0275	0.8894	-4.6368
H	1.4069	-0.7220	-5.0573
H	3.2008	-2.1209	-2.8103
H	1.5729	-2.6284	-3.2774
H	1.9282	-2.2059	-1.5781
H	3.8538	0.0188	-2.3301
H	2.6952	0.1221	-0.9853
H	2.7755	1.4162	-2.1986
H	-1.7755	-3.3676	-2.2610
H	-1.1500	-2.3621	-0.9332
H	-0.0310	-3.1293	-2.0776
H	-1.7575	-2.5688	-4.6191
H	-0.0095	-2.3208	-4.5470
H	-1.0753	-0.9978	-5.0704
H	-3.2769	-1.7560	-2.8954
H	-2.8786	-0.1081	-3.3967
H	-2.7723	-0.5719	-1.6762

66

Complex [^{t}Bu$_3$PAu-**VI**^{C}]

C	0.0000	0.0000	2.0387
AU	0.0000	0.0000	0.0000
C	1.4200	2.3479	-2.7669
C	1.5767	0.8316	-2.9672
C	2.7537	0.4043	-2.0683
P	-0.0094	-0.0455	-2.3520
C	-0.0262	-1.8775	-2.9056
C	-1.0090	-2.6510	-2.0040
C	-1.5698	0.8638	-2.9831
C	-1.7778	2.1312	-2.1316
C	1.9139	0.5482	-4.4369
C	-2.8018	-0.0215	-2.7301
C	-1.5120	1.2386	-4.4702
C	1.3602	-2.4913	-2.6471
C	-0.4029	-2.0811	-4.3792
H	-0.9359	-3.7181	-2.2611
H	-2.0510	-2.3496	-2.1305
H	-0.7442	-2.5420	-0.9431
H	1.2821	-3.5750	-2.8167
H	1.6856	-2.3411	-1.6090
H	2.1311	-2.1093	-3.3233
H	-0.3286	-3.1546	-4.6086
H	0.2680	-1.5498	-5.0628
H	-1.4320	-1.7742	-4.5945
H	-3.6958	0.5804	-2.9484
H	-2.8652	-0.3408	-1.6811
H	-2.8364	-0.9044	-3.3757

H	-2.7246	2.5960	-2.4442
H	-0.9873	2.8754	-2.2493
H	-1.8611	1.8813	-1.0651
H	-2.4749	1.6919	-4.7491
H	-1.3563	0.3690	-5.1179
H	-0.7316	1.9763	-4.6858
H	2.8007	1.1408	-4.7071
H	1.1047	0.8352	-5.1170
H	2.1606	-0.5040	-4.6148
H	3.6379	0.9834	-2.3726
H	3.0061	-0.6551	-2.1480
H	2.5481	0.6318	-1.0128
H	2.3945	2.8137	-2.9733
H	1.1455	2.5969	-1.7329
H	0.6911	2.7976	-3.4481
C	0.7202	-0.9534	2.8546
C	0.2602	-0.7591	4.3285
C	-0.3315	0.6608	4.3505
C	-0.7370	0.9264	2.8711
SI	2.6060	-0.5301	2.6250
SI	0.5652	-2.7488	2.1740
H	-0.5104	-1.5068	4.5710
H	1.0696	-0.8899	5.0578
H	-1.1682	0.7591	5.0537
H	0.4295	1.3944	4.6588
SI	-2.6201	0.5706	2.5437
SI	-0.4829	2.7470	2.2935
H	-1.1126	3.6084	3.3381
H	-1.1291	2.9554	0.9645
H	0.9869	3.0000	2.2168
H	-3.3557	1.5659	3.3784
H	-2.9082	-0.8236	2.9919
H	-2.9176	0.7396	1.0926
H	1.3407	-2.8746	0.9040
H	-0.8810	-3.0375	1.9383
H	1.1185	-3.6471	3.2307
H	2.9633	-0.5779	1.1796
H	3.3309	-1.5748	3.4081
H	2.8412	0.8290	3.1951

53

Anion **I**

Al	0.911537	-0.254218	2.008646
C	1.023202	1.931050	4.097526
C	1.488125	0.825464	4.841881
C	1.811650	0.812236	6.187592
C	1.747412	2.035997	6.871749
C	1.338026	3.178494	6.176247
C	0.976825	3.144230	4.829442
C	2.166794	-0.550971	6.796258
C	1.172978	-1.560914	6.206830
C	0.877937	-1.427686	4.861261
O	1.475732	-0.386683	4.138867
C	-0.085927	-2.158362	4.133517
C	-0.725545	-3.179385	4.880275
C	-0.419264	-3.374603	6.227198
C	0.508742	-2.579396	6.907856
N	-0.342052	-1.722916	2.856464
C	-1.101249	-2.467021	1.963861
C	2.109171	-0.523169	8.323664
C	3.601276	-0.940884	6.357714
N	0.584999	1.666796	2.823026
C	0.300283	2.681017	1.919493
H	0.605136	4.046320	4.347179
H	1.990038	2.097158	7.931234
H	0.694786	-2.740444	7.968441
H	-1.499947	-3.782373	4.409598
H	2.372545	-1.507474	8.733254
H	2.832165	0.202329	8.719752

H	1.107934	-0.251611	8.682872
H	3.857430	-1.934382	6.753080
H	3.683679	-0.971035	5.264859
H	4.323922	-0.206077	6.740729
H	1.274387	4.129565	6.709564
H	-0.943421	-4.162286	6.772870
C	-1.053261	-3.876027	1.854176
C	-1.765859	-4.544592	0.863596
C	-2.542464	-3.840726	-0.064508
C	-2.582533	-2.447264	0.015512
C	-1.879521	-1.771206	1.009552
H	-0.417633	-4.438878	2.537252
H	-1.700315	-5.633393	0.802681
H	-3.098097	-4.370184	-0.839525
H	-3.173355	-1.875833	-0.703271
H	-1.932073	-0.684302	1.080186
C	-0.710646	2.452851	0.956523
C	-0.980132	3.381178	-0.045921
C	-0.261749	4.576130	-0.123623
C	0.752940	4.811322	0.811536
C	1.036472	3.883543	1.808908
H	-1.289926	1.531328	1.023920
H	-1.767900	3.169617	-0.771883
H	-0.479367	5.307221	-0.903361
H	1.344676	5.727802	0.752518
H	1.861689	4.064455	2.496844

53

Carbene $\mathbf{I}^{C}$

C	-0.266543	0.073282	2.257957
C	0.805803	1.570333	4.081691
C	1.657400	0.689090	4.726671
C	1.953140	0.744465	6.092818
C	1.591678	1.912550	6.764314
C	0.886753	2.916891	6.083227
C	0.453516	2.741178	4.769446
C	2.368454	-0.606412	6.720699
C	1.331106	-1.574945	6.106710
C	1.097355	-1.393548	4.739366
O	1.924179	-0.503098	4.065366
C	-0.083619	-1.737416	4.103515
C	-0.970552	-2.568855	4.803718
C	-0.677815	-2.923575	6.119777
C	0.437782	-2.399226	6.790524
N	-0.393220	-1.160144	2.808852
C	-0.987274	-2.118439	1.897037
C	2.265676	-0.570122	8.244898
C	3.811262	-0.997566	6.325795
N	0.247334	1.209902	2.791545
C	0.216741	2.324591	1.864344
H	-0.173571	3.489256	4.285879
H	1.799856	2.030017	7.827244
H	0.564190	-2.594011	7.854982
H	-1.889826	-2.907520	4.327434
H	2.520359	-1.551588	8.666020
H	2.977014	0.157819	8.656615
H	1.256254	-0.299041	8.580451
H	4.054489	-1.983614	6.744123
H	3.937027	-1.038622	5.239477
H	4.519087	-0.263135	6.733133
H	0.610675	3.828190	6.614163
H	-1.370015	-3.569567	6.660307
C	-0.392549	-3.371315	1.736716
C	-0.953349	-4.297275	0.857222
C	-2.109871	-3.979605	0.144123
C	-2.700644	-2.725097	0.312284
C	-2.141417	-1.793438	1.184295
H	0.512217	-3.614281	2.293865
H	-0.476210	-5.268553	0.723353
H	-2.547279	-4.704567	-0.542628
H	-3.606266	-2.469057	-0.238672
H	-2.569878	-0.801117	1.312924
C	-0.941529	2.611074	1.141969
C	-0.954369	3.684007	0.253539
C	0.185976	4.471401	0.078853
C	1.342435	4.177615	0.802186
C	1.359635	3.108882	1.697955
H	-1.809762	1.968367	1.275491
H	-1.863700	3.907556	-0.305362
H	0.174243	5.307209	-0.620952
H	2.242730	4.777154	0.663963
H	2.261810	2.873732	2.262613

44

Anion **II**

C	-2.663163	1.677309	-2.061027
C	-1.660292	2.277636	-2.863647
C	-1.597176	3.692273	-2.836800
C	-2.495459	4.452656	-2.092828
C	-3.482408	3.836744	-1.315637
C	-3.546174	2.441246	-1.300999
N	-0.777885	1.480734	-3.591054
Si	-0.277534	1.973380	-5.179743
O	0.075300	0.591220	-6.032662
Si	-0.208263	-0.989920	-5.589810
C	0.669861	-2.023170	-6.901614
N	0.437149	-1.228663	-3.996053
C	1.072434	-2.426454	-3.665502
C	2.284091	-2.422139	-2.932716
C	2.923255	-3.607340	-2.575337
C	2.392585	-4.847568	-2.940279
C	1.190352	-4.876797	-3.654213
C	0.537939	-3.694756	-3.995652
Al	0.101886	0.034261	-2.514366
C	-2.073226	-1.279032	-5.690455
C	-1.647399	2.869897	-6.121604
C	1.299804	3.011413	-5.225480
H	-2.564407	2.263793	-6.107528
H	-2.576116	-0.636034	-4.953936
H	1.169809	3.977827	-4.717546
H	-4.182922	4.433577	-0.729837
H	2.108037	2.468254	-4.714919
H	-2.740282	0.588939	-2.065540
H	-2.414206	5.542101	-2.107354
H	-0.420172	-3.738082	-4.516958
H	-2.463826	-1.034399	-6.689832
H	-2.331716	-2.323479	-5.462693
H	-0.808657	4.194043	-3.400175
H	2.713778	-1.457396	-2.660866
H	-4.303893	1.936897	-0.697041
H	1.614737	3.204388	-6.262164
H	3.857656	-3.560242	-2.011290
H	2.901804	-5.773571	-2.669211
H	0.746759	-5.834418	-3.936839
H	1.757948	-1.896774	-6.811771
H	0.363219	-1.678145	-7.900314
H	0.446399	-3.094239	-6.817960
H	-1.341933	3.007927	-7.169693
H	-1.884986	3.852435	-5.694108

44

Carbene $\mathbf{II}^{C}$

C	-2.325890	1.471352	-1.902900
C	-1.338602	2.008988	-2.737506
C	-0.978589	3.352960	-2.599415
C	-1.612225	4.155037	-1.647967
C	-2.599521	3.620103	-0.821337
C	-2.947186	2.272880	-0.948814

N	-0.728728	1.177752	-3.738340
Si	-0.638476	1.889845	-5.394442
O	-0.237554	0.605404	-6.352975
Si	0.064719	-0.973530	-5.972249
C	1.729381	-1.483319	-6.644464
N	0.092135	-0.983910	-4.167750
C	0.659790	-2.152494	-3.554174
C	1.577082	-2.006406	-2.506651
C	2.154397	-3.129957	-1.920840
C	1.833622	-4.410903	-2.377027
C	0.917285	-4.558120	-3.417491
C	0.326795	-3.435304	-4.000671
C	-0.335828	-0.045517	-3.269333
C	-1.344674	-1.982685	-6.686552
C	-2.308118	2.566586	-5.883752
C	0.735266	3.149834	-5.588850
H	-3.079076	1.789719	-5.790103
H	-2.251480	-1.879535	-6.075181
H	0.441455	4.157718	-5.269742
H	-3.091598	4.246888	-0.077388
H	1.629285	2.849704	-5.025682
H	-2.577024	0.417546	-2.013816
H	-1.316479	5.199464	-1.543557
H	-0.420974	-3.564104	-4.781530
H	-1.568445	-1.600462	-7.693497
H	-1.105974	-3.049850	-6.778498
H	-0.178099	3.768147	-3.209252
H	1.808452	-1.000000	-2.161126
H	-3.714429	1.843508	-0.303442
H	1.006508	3.199440	-6.653857
H	2.866955	-3.003820	-1.104805
H	2.291874	-5.288403	-1.920489
H	0.642628	-5.553014	-3.769570
H	2.522524	-0.852437	-6.220815
H	1.744525	-1.369697	-7.738063
H	1.957453	-2.529183	-6.401834
H	-2.286633	2.908489	-6.928579
H	-2.597084	3.413960	-5.248421

49

Anion **III**

Al	-0.147138	-0.053510	2.093348
H	0.941293	-2.514372	0.946218
C	-2.699977	2.020203	4.566132
C	-1.874867	1.859525	3.426414
C	-2.426221	2.273073	2.186795
C	-3.706765	2.811243	2.102097
C	-4.496721	2.975386	3.245007
C	-3.974437	2.578174	4.477983
N	-0.595884	1.300306	3.491543
Si	0.516837	1.832763	4.725665
C	2.279165	1.726794	4.048916
N	0.786595	-1.316854	3.313911
Si	0.261050	-1.697848	4.934132
C	-1.625370	-1.619890	5.018841
H	3.215922	-1.571218	4.469751
C	1.912208	-1.963470	2.790695
C	1.876877	-2.558557	1.504647
C	2.992600	-3.192896	0.965319
C	4.190870	-3.274682	1.681948
C	4.250631	-2.693357	2.950656
C	3.141861	-2.040407	3.487475
C	0.443313	0.854922	6.362230
C	0.984749	-0.585074	6.304167
C	0.202064	3.652210	5.165625
C	0.747047	-3.478904	5.378762
H	-4.089759	3.121591	1.126858
H	-5.496640	3.406128	3.175452
H	-4.570989	2.692904	5.386107
H	0.181925	4.263215	4.251426
H	1.008093	4.028876	5.813787
H	-0.754767	3.798586	5.683938
H	2.530128	0.710076	3.720485
H	3.004558	2.027964	4.820726
H	2.396273	2.399082	3.186734
H	0.980923	1.446484	7.126234
H	-0.611109	0.842357	6.685530
H	0.824900	-1.103559	7.267571
H	2.077717	-0.566714	6.156147
H	-2.015805	-0.636281	4.729783
H	-1.969816	-1.840151	6.041372
H	-2.061545	-2.369400	4.342380
H	0.414783	-4.168508	4.589058
H	0.265017	-3.777806	6.322125
H	1.832382	-3.602782	5.491050
H	2.920589	-3.644629	-0.026996
H	5.059287	-3.783235	1.260294
H	5.176218	-2.739849	3.529386
H	-1.811781	2.164716	1.292292
H	-2.322712	1.692025	5.535819

49

Carbene **III**$^{\mathbf{C}}$

C	-0.009110	-0.057437	2.993459
H	0.015744	-2.237323	1.459383
C	-2.828307	1.746831	4.065861
C	-1.692218	1.569743	3.272746
C	-1.673659	2.064586	1.964975
C	-2.780971	2.747420	1.468150
C	-3.915002	2.936834	2.263707
C	-3.935721	2.432235	3.562940
N	-0.553963	0.880853	3.826062
Si	0.359046	1.778734	5.132155
C	2.106774	2.038066	4.487797
N	0.858719	-0.906696	3.621449
Si	0.590876	-1.626853	5.281703
C	-1.257925	-1.937462	5.438736
H	3.478650	-1.266290	3.789732
C	1.659909	-1.699324	2.722946
C	1.082999	-2.363179	1.636101
C	1.878121	-3.141869	0.799294
C	3.251395	-3.260900	1.033965
C	3.826217	-2.588390	2.111180
C	3.033315	-1.806257	2.952812
C	0.390419	0.819696	6.756518
C	1.189309	-0.489257	6.662070
C	-0.444736	3.455871	5.390245
C	1.488032	-3.273356	5.378454
H	-2.758600	3.138407	0.450055
H	-4.779323	3.471404	1.868964
H	-4.819337	2.562799	4.188618
H	-0.612924	3.974278	4.437244
H	0.235124	4.070606	5.999689
H	-1.407373	3.388623	5.911420
H	2.605386	1.094826	4.238204
H	2.706633	2.562561	5.246073
H	2.085197	2.657813	3.580724
H	0.804204	1.485759	7.532822
H	-0.650940	0.616324	7.053581
H	1.136290	-1.059304	7.605580
H	2.257605	-0.278934	6.493743
H	-1.849139	-1.022870	5.316165
H	-1.479883	-2.369385	6.425647
H	-1.584187	-2.653611	4.671809
H	1.256110	-3.911540	4.515813
H	1.151112	-3.792306	6.288841
H	2.577782	-3.160785	5.431050
H	1.421878	-3.664356	-0.042585

H	3.869810	-3.871300	0.375468
H	4.898295	-2.664094	2.296004
H	-0.789327	1.884307	1.355612
H	-2.843902	1.336820	5.076797

36

Anion **IV**

Al	0.074486	0.510521	2.255615
C	-3.500541	-0.739045	3.295994
C	-2.609903	0.344952	3.157311
C	-3.046216	1.459141	2.415714
C	-4.311542	1.481385	1.824420
C	-5.182467	0.403065	1.972534
C	-4.763990	-0.708738	2.715883
N	-1.287937	0.293452	3.645305
C	-1.105617	0.044944	5.010398
C	0.247690	-0.206694	5.482733
C	1.414836	-0.357116	4.788350
N	1.525223	-0.212714	3.412234
C	2.622853	-0.761873	2.743436
C	3.263061	-0.029405	1.717064
C	4.314238	-0.578115	0.987103
C	4.775819	-1.870510	1.255067
C	4.143648	-2.614700	2.256605
C	3.079776	-2.079077	2.977598
H	4.786667	0.017643	0.202901
H	5.605636	-2.294173	0.687482
H	4.471156	-3.635980	2.465021
H	2.911997	0.981865	1.510600
H	2.558022	-2.689875	3.714926
H	0.332649	-0.247306	6.569021
C	2.689212	-0.535657	5.591806
H	-2.365368	2.303080	2.303974
H	-4.617838	2.355963	1.246280
H	-6.172705	0.421528	1.514177
H	-5.429504	-1.567325	2.831058
H	-3.171369	-1.607744	3.866469
C	-2.113337	0.090744	5.941502
H	-1.882365	-0.097816	6.988572
H	-3.142851	0.317748	5.678767
H	2.497568	-0.318381	6.649794
H	3.467212	0.150030	5.222372
H	3.110768	-1.547810	5.521214

36

Carbene **IV**^{C}

C	-0.022333	-0.295635	2.924002
C	-2.843754	-0.746421	2.250574
C	-2.314471	0.241996	3.077245
C	-2.981243	1.455860	3.248274
C	-4.185626	1.678647	2.584529
C	-4.724843	0.692231	1.755580
C	-4.050983	-0.518114	1.590599
N	-1.050940	0.021094	3.736132
C	-1.014887	0.079741	5.173737
C	0.332408	0.162558	5.683745
C	1.409350	-0.089983	4.909181
N	1.176864	-0.445459	3.557707
C	2.266451	-0.895888	2.735847
C	2.560627	-0.226226	1.546912
C	3.610467	-0.670862	0.746346
C	4.366225	-1.782795	1.125910
C	4.057739	-2.457552	2.307390
C	3.005160	-2.020054	3.111303
H	3.840180	-0.144925	-0.180802
H	5.187045	-2.127964	0.497015
H	4.627797	-3.339851	2.599711
H	1.944481	0.624712	1.260276
H	2.736753	-2.563950	4.017036
H	0.473152	0.454757	6.722819
C	2.816051	0.098938	5.384565
H	-2.552201	2.217441	3.898811
H	-4.701272	2.631157	2.709737
H	-5.667190	0.869868	1.236854
H	-4.463535	-1.290280	0.940698
H	-2.287687	-1.673183	2.117305
C	-2.121587	0.063678	5.948470
H	-2.008389	0.088035	7.030230
H	-3.125285	0.027333	5.536818
H	2.806579	0.592496	6.363210
H	3.383989	0.721230	4.678115
H	3.362937	-0.848535	5.478037

40

Anion **V**

Al	0.025463	0.048788	2.226187
H	-5.050559	1.851030	1.736226
C	-5.671382	-0.419036	4.286836
C	-4.938543	-1.605384	3.637986
C	-5.191444	-1.593307	2.122481
C	-4.659248	-0.278148	1.531504
C	-5.404350	0.903162	2.172861
C	-5.149556	0.898048	3.688069
C	-3.429863	-1.483868	3.903407
C	-2.862410	-0.162923	3.324376
C	-3.154372	-0.156112	1.812468
C	-3.640118	1.021542	3.952739
N	-1.420721	-0.052308	3.547819
C	-0.989869	-0.041258	4.862474
C	0.336235	0.027706	5.150971
C	1.280553	0.080632	3.988976
Si	2.253821	1.654027	3.970777
H	3.035724	1.894718	5.246293
Si	2.333269	-1.442739	3.940755
H	3.114008	-1.671049	5.218752
H	1.508712	-2.693211	3.762369
H	3.364638	-1.438106	2.840274
H	1.371411	2.868992	3.826564
H	3.272817	1.718587	2.861305
H	0.671564	0.038506	6.194563
H	-1.728127	-0.088744	5.673817
H	-2.600108	-0.981391	1.337721
H	-2.748855	0.769638	1.374232
H	-3.233696	-1.526594	4.985583
H	-2.881895	-2.321070	3.443172
H	-3.243258	1.956095	3.526018
H	-3.451640	1.056069	5.036449
H	-4.823497	-0.270214	0.441413
H	-4.681668	-2.448945	1.651316
H	-6.270769	-1.696039	1.914872
H	-5.313207	-2.549559	4.067938
H	-6.758392	-0.506829	4.118039
H	-5.506657	-0.426954	5.376885
H	-5.675769	1.748266	4.154047
H	-6.486493	0.828455	1.968029

40

Carbene V^{C}

C	-0.236819	-0.020782	3.107482
H	-4.641658	1.913472	1.700695
C	-5.583306	-0.419995	4.095539
C	-4.784999	-1.595678	3.507907
C	-4.847744	-1.542310	1.973560
C	-4.244319	-0.215519	1.486333
C	-5.050877	0.957813	2.064386
C	-4.987728	0.907833	3.598501
C	-3.319742	-1.496253	3.963025
C	-2.713008	-0.166055	3.474710

C	-2.779902	-0.115264	1.943091
C	-3.522420	1.012097	4.052952
N	-1.300642	-0.071847	3.926185
C	-0.973071	-0.041434	5.305937
C	0.368116	0.039239	5.432762
C	0.912983	0.055138	4.049744
Si	1.815082	1.648847	3.536446
H	2.963520	1.871227	4.475716
Si	1.965522	-1.464570	3.597490
H	3.126338	-1.538253	4.544763
H	1.134331	-2.696154	3.767610
H	2.475302	-1.356691	2.199586
H	0.873108	2.803865	3.657750
H	2.333399	1.531169	2.142537
H	0.927661	0.081202	6.363438
H	-1.740056	-0.081260	6.073722
H	-2.175853	-0.932269	1.525871
H	-2.316028	0.816609	1.590752
H	-3.262843	-1.558757	5.061051
H	-2.726272	-2.328891	3.555198
H	-3.073097	1.956855	3.710022
H	-3.474482	1.005694	5.152630
H	-4.273676	-0.177699	0.386891
H	-4.292101	-2.390297	1.543341
H	-5.892637	-1.631237	1.636470
H	-5.207664	-2.546865	3.865920
H	-6.639236	-0.494222	3.791685
H	-5.558833	-0.458145	5.196659
H	-5.554483	1.750598	4.023113
H	-6.098247	0.901473	1.727978

25

Anion **VI**

Al	-0.011273	0.039904	2.193563
C	0.032955	-1.055464	5.009041
H	-2.930612	-2.354822	2.483262
H	-3.002571	-1.860868	4.856821
H	0.428827	3.578568	2.493487
H	-1.622413	2.961721	3.634763
H	1.105727	-1.317492	5.082658
H	2.862195	-0.167887	3.240823
Si	2.344905	1.096564	3.890457
C	0.491144	1.191100	3.950524
C	-0.511218	-1.494079	3.630946
H	-2.929189	-0.079935	3.249440
C	-0.103538	0.463508	5.176764
H	-0.342001	-3.492278	1.677723
H	-0.464688	-1.572076	5.853236
H	-0.145945	-4.293525	3.936169
H	2.957835	1.111207	5.273373
H	2.970874	2.226453	3.118615
Si	-2.366134	-1.451998	3.545962
H	1.671865	-3.077293	2.969969
Si	0.164854	-3.108435	3.039015
Si	-0.116294	2.920665	3.729130
H	-1.178582	0.709585	5.266214
H	0.362202	0.777362	6.131764
H	0.213585	3.866335	4.870470

25

Carbene **VI**^{C}

C	0.007695	-0.078924	3.118006
C	-0.128150	-1.109274	5.368415
H	-2.234497	-1.630986	1.740933
H	-2.999017	-1.923532	4.046816
H	-0.229615	2.938654	2.230596
H	-1.697948	2.751728	4.191502
H	0.775176	-1.668613	5.660626
H	2.684519	-0.486822	3.188082
Si	2.165545	0.897505	3.415477
C	0.344412	0.928374	4.101920
C	-0.352246	-1.281940	3.837253
H	-2.672435	0.313413	3.203873
C	0.085664	0.410522	5.546945
H	0.248592	-2.820074	1.568775
H	-0.954952	-1.496967	5.977763
H	-0.563875	-4.054168	3.520092
H	2.982577	1.500131	4.524579
H	2.282453	1.738568	2.191248
Si	-2.153717	-1.087427	3.125811
H	1.670849	-3.107846	3.552342
Si	0.271551	-2.897989	3.057406
Si	-0.284958	2.674480	3.697098
H	-0.825263	0.890960	5.938423
H	0.897598	0.646607	6.247101
H	0.521774	3.703532	4.430797

66

Complex **VI**^{B}

C	-2.688302793	1.146723909	2.643408000
C	-2.863261021	-0.173514002	1.873976907
C	-1.921075331	-1.185132756	2.558722807
P	-2.245979551	0.086774295	0.076768061
C	-2.800245076	-1.390273700	-1.014083800
C	-1.906043832	-1.411682968	-2.271000158
AU	0.164161498	0.185652581	0.067620283
B	2.240066474	0.192962688	0.034407344
C	-2.967066744	1.718057146	-0.627307067
C	-2.141707742	2.887441608	-0.052199100
C	-4.316908172	-0.644722134	2.006880278
C	-2.720668702	1.742532206	-2.145288685
C	-4.458157988	1.950503149	-0.354153728
C	-2.491596674	-2.696781858	-0.263267274
C	-4.278050659	-1.383699937	-1.425098057
H	-2.117100165	-2.338575031	-2.826063820
H	-2.082809310	-0.571114542	-2.946188346
H	-0.841836864	-1.412212941	-1.994406329
H	-2.639554152	-3.532212375	-0.964085490
H	-1.448034542	-2.729012397	0.077814186
H	-3.154066791	-2.865082881	0.591742901
H	-4.487618333	-2.301068285	-1.997338779
H	-4.952952717	-1.370151041	-0.561823934
H	-4.527902448	-0.534304075	-2.070723322
H	-2.964880657	2.750837508	-2.511990015
H	-1.666747101	1.546582445	-2.384963425
H	-3.349724145	1.032783619	-2.692153992
H	-2.443964029	3.808559718	-0.573726360
H	-2.297418812	3.044983667	1.017707757
H	-1.067112115	2.732783774	-0.223255818
H	-4.764679550	2.884938748	-0.849893685
H	-5.087499224	1.144209263	-0.747441396
H	-4.672452488	2.064814777	0.714517662
H	-4.568882446	-0.708693247	3.077049026
H	-5.026159192	0.047869697	1.539596689
H	-4.473100349	-1.640221329	1.576476001
H	-2.173345514	-1.217347757	3.629682611
H	-2.011525940	-2.199983919	2.164689173
H	-0.871013121	-0.874686744	2.461461274
H	-2.867436577	0.944405606	3.710035289
H	-1.666967559	1.538187815	2.544351306
H	-3.398299168	1.919663849	2.331761032
C	3.165645422	-1.017287524	0.435834697
C	4.599876198	-0.644860755	-0.040196929
C	4.644026536	0.893905416	-0.067409220
C	3.200057446	1.352869556	-0.425534513
SI	2.965700726	-1.041524826	2.319234866
SI	2.480872230	-2.620926982	-0.266929265
H	4.752825590	-1.034051427	-1.061423093

H	5.399666228	-1.072398131	0.582822895
H	5.413739230	1.275813951	-0.754657841
H	4.902890935	1.271345640	0.936820507
SI	2.854561976	1.419847964	-2.286309172
SI	2.693501142	2.994898342	0.334203098
H	3.625567863	4.098691318	-0.088147378
H	1.304544978	3.389377973	-0.060871706
H	2.769109270	2.907097033	1.830130159
H	3.965312141	2.172011197	-2.964417365
H	2.804277150	0.051818048	-2.900422808
H	1.557537959	2.110202643	-2.573638002
H	1.132525216	-2.947813751	0.296963426
H	2.369173321	-2.521629949	-1.760218889
H	3.398371667	-3.777731633	0.027779029
H	1.665683746	-1.664308429	2.721779162
H	4.090634618	-1.835120534	2.922068779
H	3.027218816	0.336379610	2.909988492

H	5.518547265	-1.298994576	-0.417781921
H	5.264946937	1.121387881	-1.320310240
H	5.246381013	0.961986626	0.434088900
SI	2.425526037	1.507805359	-2.104960202
SI	3.227174858	3.062336398	0.446471194
H	3.804126608	4.116765684	-0.445187640
H	1.807538964	3.409044354	0.770138924
H	4.029559990	2.971660326	1.708418787
H	3.432164398	2.030416933	-3.078046245
H	1.938238207	0.162371174	-2.561742752
H	1.245374447	2.421097337	-2.002380055
H	1.646022608	-3.323236818	0.973755799
H	2.106986103	-2.944349768	-1.397056187
H	3.757400840	-4.086267574	-0.000772032
H	3.092003757	-1.863059652	3.162543566
H	5.048540246	-0.371515104	2.586102243
H	2.743444623	0.311487310	2.209164411

66

Complex **VI**^{Si}

C	-3.235771365	1.092450995	2.714827401
C	-3.335971843	-0.196837321	1.882856973
C	-2.535313590	-1.264895151	2.653748515
P	-2.488223614	0.083330961	0.192043170
C	-2.896112302	-1.337362050	-1.022088740
C	-1.836286057	-1.350616605	-2.142108585
AU	-0.128772832	0.082339284	0.506581714
SI	2.193876588	0.103222815	0.469661801
C	-2.980413826	1.776264269	-0.549498794
C	-2.190876370	2.878540489	0.184003233
C	-4.807748187	-0.617718155	1.776757162
C	-2.527593372	1.836095230	-2.018010435
C	-4.485541051	2.066892665	-0.472615242
C	-2.750938320	-2.679270255	-0.285683128
C	-4.300265291	-1.240194583	-1.633539498
H	-2.027362028	-2.229485481	-2.775424556
H	-1.863090639	-0.467188459	-2.783310851
H	-0.821900155	-1.447817780	-1.729144973
H	-2.837718766	-3.481160196	-1.033195963
H	-1.766800732	-2.777501519	0.192124018
H	-3.531067358	-2.845099218	0.463575105
H	-4.464691782	-2.126933298	-2.263730269
H	-5.089454031	-1.223166333	-0.874084804
H	-4.416360479	-0.360129318	-2.275165983
H	-2.682022772	2.865917173	-2.371708728
H	-1.459101746	1.606278523	-2.125655259
H	-3.104859765	1.175968905	-2.672559933
H	-2.407360086	3.836328381	-0.311608323
H	-2.457160179	2.982633986	1.237966481
H	-1.107597795	2.702058577	0.116066478
H	-4.676746571	3.029674236	-0.969494092
H	-5.086583041	1.306316846	-0.982756720
H	-4.843285095	2.157434760	0.558614368
H	-5.216793388	-0.698526491	2.794931927
H	-5.415325460	0.114344601	1.233543368
H	-4.931234114	-1.595328043	1.298758335
H	-2.965957227	-1.348358681	3.662507838
H	-2.568833796	-2.255287410	2.194858182
H	-1.481651189	-0.968889698	2.762206143
H	-3.589566433	0.861265352	3.730114532
H	-2.199250585	1.445483000	2.798690129
H	-3.860250896	1.903359171	2.327960644
C	3.456476083	-1.332598898	0.372854723
C	4.549982581	-0.813286929	-0.603265999
C	4.681075651	0.716961555	-0.480265481
C	3.271938702	1.369337782	-0.396504800
SI	3.744776779	-0.959141191	2.180624963
SI	2.713892843	-3.014919059	-0.029145887
H	4.255707789	-1.075460173	-1.632091196

102

Complex **VI real**

C	3.697366	1.227882	-2.402178
C	3.865126	-0.212775	-1.889873
C	5.299102	-0.680844	-2.166596
C	2.868905	-1.064777	-2.703542
P	3.320730	-0.274152	-0.051108
C	4.107379	1.192917	0.903568
C	5.591696	1.447198	0.612986
C	3.879081	-1.930353	0.735294
C	5.370331	-2.026096	1.080054
AU	0.897027	-0.090505	0.040919
AL	-1.504465	0.186315	0.036337
SI	-2.453656	2.458857	1.726113
C	-2.600856	1.860570	-0.064833
SI	-2.057510	3.229688	-1.245004
C	3.029815	-2.155698	2.003407
C	3.506809	-3.075404	-0.221548
C	-3.033490	-1.113531	0.013022
SI	-3.017431	-2.539458	1.256106
SI	-2.826812	-1.819636	-1.731936
C	3.285857	2.458377	0.582950
C	3.922549	0.949498	2.411004
C	-4.248092	-0.144894	0.174596
C	-4.004824	1.268145	-0.412958
H	3.234850	-3.170252	2.378245
H	3.254153	-1.452318	2.808654
H	1.956819	-2.086168	1.774531
H	3.656245	-4.025646	0.312843
H	2.451087	-3.023118	-0.520252
H	4.131438	-3.102172	-1.120352
H	5.576872	-3.033241	1.475341
H	6.012582	-1.874531	0.205085
H	5.666027	-1.307599	1.852890
H	4.206674	1.871662	2.940069
H	2.874687	0.732148	2.658027
H	4.554682	0.142441	2.795119
H	3.628531	3.268143	1.245153
H	3.396123	2.801520	-0.448232
H	2.216231	2.288661	0.770509
H	5.938934	2.273523	1.253240
H	6.216617	0.573486	0.830183
H	5.767075	1.746388	-0.426436
H	5.510070	-0.555228	-3.240322
H	6.043082	-0.097993	-1.611705
H	5.444833	-1.740564	-1.928913
H	3.074746	-0.907167	-3.773397
H	2.948032	-2.135991	-2.503445
H	1.832904	-0.754667	-2.502730
H	3.831648	1.216827	-3.494326

H	2.691796	1.616975	-2.192781
H	4.439347	1.917741	-1.987372
H	-4.470822	-0.029247	1.247156
H	-5.170131	-0.553102	-0.275964
H	-4.827559	1.926025	-0.081720
H	-4.100581	1.202112	-1.507899
C	-2.800125	4.924141	-0.796618
C	-0.172853	3.398071	-1.356141
C	-2.640107	2.975412	-3.034238
C	-4.018427	3.350285	2.309395
C	-2.178207	1.021369	2.940745
C	-0.938972	3.557102	2.034874
C	-1.253580	-3.020580	1.755130
C	-3.949038	-2.156550	2.867662
C	-3.915445	-4.108999	0.655436
C	-1.464944	-3.138132	-1.804850
C	-4.449362	-2.476895	-2.452131
C	-2.216470	-0.525095	-2.983713
H	-2.454581	5.680372	-1.517848
H	-3.897957	4.880871	-0.860386
H	-2.537823	5.274498	0.209702
H	-2.451539	3.914966	-3.575873
H	-2.090772	2.176841	-3.546688
H	-3.715129	2.761410	-3.111438
H	0.087491	4.154844	-2.111562
H	0.291325	3.689089	-0.406597
H	0.274658	2.438720	-1.657378
H	-3.888550	3.700677	3.344089
H	-4.250673	4.218318	1.678184
H	-4.885654	2.674848	2.290041
H	-0.922624	3.820089	3.103424
H	-0.018767	2.996510	1.814975
H	-0.920718	4.487870	1.456102
H	-2.016441	1.431586	3.949609
H	-3.043311	0.352186	2.983767
H	-1.286387	0.416440	2.701338
H	-1.268948	-3.895129	2.422824
H	-0.624670	-3.258186	0.887846
H	-0.770228	-2.189795	2.290144
H	-3.829230	-4.882387	1.434125
H	-4.986382	-3.900086	0.513704
H	-3.528934	-4.530121	-0.280354
H	-4.106145	-3.112771	3.389682
H	-3.392824	-1.499187	3.546464
H	-4.939091	-1.714417	2.688700
H	-1.367424	-3.483745	-2.845011
H	-0.507351	-2.680295	-1.511716
H	-1.632248	-4.014571	-1.168534
H	-4.275644	-2.874829	-3.463112
H	-4.887465	-3.273227	-1.838027
H	-5.187741	-1.666083	-2.535297
H	-1.985666	-1.037320	-3.930667
H	-2.973402	0.239989	-3.189122
H	-1.290925	-0.014686	-2.668407

105

RC (VI real)

C	3.219929	-0.355900	3.057548
C	3.585252	0.705928	2.006105
P	3.279734	-0.031027	0.259160
AU	0.863097	-0.127342	-0.059671
AL	-1.549511	0.038078	0.056607
C	-2.646901	1.710686	0.162974
C	-4.059211	1.106456	0.464845
C	-3.995005	-0.246267	1.222059
C	-2.993286	-1.253394	0.569050
SI	-3.710444	-2.202238	-0.903548
C	5.012689	1.204980	2.263431
C	2.588010	1.861441	2.220717
C	4.061743	-1.780589	0.162454
C	4.129044	-2.218650	-1.310016
C	4.070018	1.108386	-1.064290
C	3.681996	2.565698	-0.762598
C	5.457178	-1.911830	0.785197
C	3.085952	-2.761122	0.845191
C	5.596103	1.009893	-1.187217
C	3.407490	0.774539	-2.416290
SI	-2.218723	-2.458414	1.800405
SI	-2.535745	2.616514	-1.493139
SI	-2.046804	2.818493	1.574535
O	0.069870	-0.230669	-3.913373
C	0.559208	-1.235335	-3.567042
O	1.055229	-2.242469	-3.236108
H	3.776830	1.488152	-3.168477
H	3.630294	-0.233412	-2.774436
H	2.316113	0.888789	-2.349430
H	3.973184	3.179512	-1.628279
H	2.597440	2.675071	-0.627417
H	4.195584	2.971107	0.115170
H	5.936215	1.736761	-1.941462
H	6.105654	1.244841	-0.246052
H	5.926126	0.019154	-1.518734
H	4.421941	-3.279234	-1.331649
H	3.152067	-2.136490	-1.801774
H	4.871751	-1.662167	-1.890658
H	3.454662	-3.785007	0.679854
H	2.995871	-2.608233	1.923438
H	2.081875	-2.683830	0.406090
H	5.815585	-2.940975	0.625933
H	6.184356	-1.232331	0.325870
H	5.452610	-1.733287	1.865951
H	5.079331	1.552541	3.306341
H	5.764745	0.420262	2.126094
H	5.277210	2.052771	1.621471
H	2.661481	2.188342	3.269233
H	2.781291	2.729928	1.587285
H	1.556546	1.529796	2.038171
H	3.225884	0.129877	4.044834
H	2.211028	-0.757047	2.890830
H	3.931836	-1.186481	3.095352
H	-4.595724	0.934370	-0.479272
H	-4.690084	1.802504	1.043051
H	-5.015497	-0.661892	1.290554
H	-3.691964	-0.040985	2.263651
C	-3.525409	-3.373958	2.826227
C	-1.058623	-3.679849	0.930013
C	-1.108334	-1.615579	3.089440
C	-4.380728	-3.937914	-0.504170
C	-5.231054	-1.359966	-1.659979
C	-2.411071	-2.405388	-2.270191
C	-0.756144	2.843741	-2.110684
C	-3.413981	1.647607	-2.867682
C	-3.364743	4.324278	-1.487321
C	-0.522443	3.846793	1.117390
C	-3.410985	4.013596	2.133171
C	-1.544928	1.881549	3.144315
H	-3.038252	-3.982133	3.603243
H	-4.179549	-2.648086	3.332068
H	-4.158482	-4.033034	2.220269
H	-0.590111	-4.335564	1.678898
H	-1.545986	-4.314172	0.180014
H	-0.258099	-3.113979	0.429588
H	-0.675954	-2.399199	3.730550
H	-0.274207	-1.077272	2.613485
H	-1.649602	-0.914197	3.736087
H	-4.732455	-4.390216	-1.444283
H	-3.649828	-4.620855	-0.055517

H	-5.243576	-3.870041	0.174052
H	-5.675378	-2.049887	-2.393389
H	-5.993431	-1.154358	-0.894823
H	-5.001480	-0.422795	-2.177297
H	-2.799366	-3.027025	-3.090450
H	-2.139362	-1.428400	-2.697782
H	-1.494288	-2.873796	-1.888110
H	-3.004039	4.701408	2.889813
H	-3.805017	4.612521	1.302174
H	-4.251976	3.475983	2.593730
H	-0.146385	4.337456	2.027481
H	0.278626	3.210197	0.718363
H	-0.741259	4.628357	0.378285
H	-1.231882	2.610733	3.907347
H	-2.375243	1.296074	3.561680
H	-0.701772	1.199662	2.971332
H	-3.356478	4.745374	-2.504160
H	-4.412148	4.252281	-1.159788
H	-2.851583	5.034697	-0.824948
H	-3.228271	2.160528	-3.823220
H	-3.038923	0.619674	-2.967582
H	-4.501776	1.602934	-2.722105
H	-0.785765	3.148453	-3.167577
H	-0.204725	3.608284	-1.551521
H	-0.187398	1.904619	-2.044383

105

TSI (VI real)

C	3.523417	-1.297526	2.616760
C	3.813670	0.043282	1.921224
P	3.354490	-0.108900	0.064975
AU	0.921930	-0.136212	-0.205296
AL	-1.516146	0.034004	-0.113617
O	-1.602566	-0.081657	-2.479888
C	-0.502674	-0.509882	-2.674046
O	0.431390	-0.969958	-3.218622
C	5.266538	0.448146	2.201837
C	2.852565	1.064816	2.560940
C	4.069784	-1.741210	-0.644031
C	3.977316	-1.694766	-2.178467
C	4.068694	1.387177	-0.901388
C	3.761446	2.677820	-0.123294
C	5.514925	-2.055111	-0.236496
C	3.138209	-2.889030	-0.202723
C	5.574800	1.319970	-1.184309
C	3.289661	1.505197	-2.227646
C	-2.907120	-1.297122	0.447875
C	-3.826635	-0.324960	1.271761
C	-3.904514	1.102109	0.671189
C	-2.500679	1.718539	0.357655
SI	-1.734078	2.501453	1.904113
SI	-3.902004	-2.135309	-0.931395
SI	-2.136729	-2.642135	1.544559
SI	-2.523492	2.977302	-1.055668
H	3.620419	2.421936	-2.739329
H	3.451207	0.665143	-2.906991
H	2.210293	1.591389	-2.039126
H	4.014385	3.530680	-0.770924
H	2.695269	2.756564	0.126562
H	4.350670	2.776624	0.794001
H	5.876438	2.246441	-1.697563
H	6.169065	1.240044	-0.266965
H	5.840137	0.483891	-1.840597
H	4.225114	-2.696428	-2.560905
H	2.961570	-1.452186	-2.515941
H	4.682519	-0.988594	-2.628508
H	3.452310	-3.806299	-0.723703
H	3.169000	-3.087447	0.871120
H	2.095799	-2.677070	-0.481138
H	5.826682	-2.983910	-0.739347
H	6.213869	-1.265510	-0.534513
H	5.618794	-2.219168	0.841830
H	5.423745	0.455539	3.291793
H	5.988181	-0.252674	1.767270
H	5.498008	1.453825	1.833989
H	3.003168	1.045553	3.651053
H	3.014722	2.090025	2.220269
H	1.806179	0.801037	2.358024
H	3.624782	-1.143221	3.701599
H	2.498062	-1.640716	2.426193
H	4.224493	-2.088263	2.331590
H	-4.493679	1.045641	-0.254341
H	-4.486275	1.737762	1.360727
H	-4.853311	-0.721500	1.366218
H	-3.454585	-0.240136	2.306302
C	-3.382447	-3.287073	2.822854
C	-1.459323	-4.106795	0.547658
C	-0.639724	-2.108256	2.584439
C	-5.052711	-3.490511	-0.254398
C	-5.097006	-0.964018	-1.819346
C	-2.819566	-2.934245	-2.279474
C	-0.820471	3.229004	-1.857026
C	-3.708785	2.480751	-2.449715
C	-3.126191	4.706366	-0.545516
C	-0.173279	3.500922	1.510362
C	-2.975554	3.615356	2.809093
C	-1.164219	1.270702	3.226495
H	-1.072169	-4.852555	1.258224
H	-2.198293	-4.601301	-0.094147
H	-0.619113	-3.784254	-0.084629
H	-0.188978	-3.019083	3.008232
H	0.125253	-1.607135	1.969674
H	-0.905460	-1.446018	3.416063
H	-2.886137	-4.014177	3.483197
H	-3.750976	-2.464637	3.453052
H	-4.250990	-3.775992	2.365383
H	-3.188167	-3.943971	-2.511026
H	-2.863675	-2.341899	-3.203399
H	-1.767911	-3.028597	-1.982795
H	-5.693755	-3.847330	-1.075072
H	-4.510444	-4.355739	0.148412
H	-5.709335	-3.101122	0.536808
H	-5.634550	-1.545693	-2.584223
H	-5.846673	-0.543698	-1.134390
H	-4.586887	-0.135775	-2.324091
H	-3.160305	5.349673	-1.438126
H	-4.140373	4.665961	-0.122422
H	-2.470148	5.189898	0.190290
H	-0.946138	3.797052	-2.791091
H	-0.137755	3.792110	-1.209470
H	-0.330472	2.277712	-2.101547
H	-3.706694	3.290203	-3.195560
H	-3.403659	1.558139	-2.957597
H	-4.742443	2.358283	-2.098435
H	-2.468152	4.106925	3.653019
H	-3.403054	4.393618	2.165769
H	-3.803841	3.022977	3.223994
H	0.315866	3.781740	2.454853
H	0.530966	2.883519	0.934333
H	-0.370377	4.419368	0.943462
H	-0.741156	1.839096	4.069096
H	-1.983702	0.653226	3.617201
H	-0.380634	0.601272	2.851391

105

INT (VI real)

C	3.188271	-2.008374	1.996755

C	3.564741	-0.520851	1.901351
C	2.575241	0.253637	2.793380
P	3.292577	0.059619	0.101522
C	4.077085	1.795641	-0.155742
C	3.375482	2.451066	-1.362069
C	4.993500	-0.332874	2.429251
AU	0.975642	0.092389	-0.629973
C	-0.402632	0.014381	-2.218475
O	0.179170	0.060365	-3.293064
C	4.097776	-1.179307	-1.133902
C	3.188857	-2.416396	-1.266622
AL	-1.716689	-0.005906	-0.200170
C	-2.598167	1.544257	0.704622
SI	-3.332023	2.812267	-0.512815
O	-1.691895	-0.108081	-2.044883
C	-2.597552	-1.528717	0.739995
SI	-1.428781	-2.832515	1.453008
C	4.131093	-0.544537	-2.535738
C	5.509084	-1.635073	-0.738042
C	3.788728	2.700242	1.053200
C	5.596282	1.770320	-0.376594
C	-3.314149	-0.695902	1.857377
C	-3.747302	0.721593	1.396659
SI	-3.879138	-2.379674	-0.389288
SI	-1.561153	2.457332	2.003923
H	3.795845	3.459172	-1.497067
H	3.509655	1.904323	-2.298633
H	2.297231	2.557853	-1.180006
H	4.108940	3.718553	0.786107
H	2.722081	2.744400	1.292505
H	4.348006	2.407606	1.947511
H	5.945475	2.810874	-0.460645
H	6.130555	1.310153	0.462732
H	5.887038	1.255919	-1.297602
H	4.449503	-1.323617	-3.244274
H	3.135336	-0.203327	-2.850971
H	4.838859	0.284787	-2.622867
H	3.652906	-3.096549	-1.996749
H	3.052987	-2.969146	-0.334919
H	2.198145	-2.141494	-1.652290
H	5.902198	-2.273032	-1.544315
H	6.207081	-0.802521	-0.604355
H	5.505742	-2.237195	0.177684
H	5.032744	-0.716583	3.460325
H	5.733889	-0.885987	1.841808
H	5.294148	0.719652	2.462733
H	2.532529	-0.240630	3.774808
H	2.869785	1.293197	2.954810
H	1.563594	0.249175	2.368776
H	3.168383	-2.284736	3.061197
H	2.191987	-2.208000	1.584792
H	3.916594	-2.658345	1.500931
H	-4.596440	0.608668	0.702203
H	-4.153335	1.260619	2.270100
H	-4.205506	-1.219312	2.240234
H	-2.642496	-0.578258	2.718002
C	-2.290394	-4.392269	2.107706
C	-0.120706	-3.389050	0.205273
C	-0.470789	-2.167835	2.945682
C	-5.245472	-3.188734	0.650650
C	-4.818243	-1.240304	-1.570325
C	-3.103499	-3.689870	-1.514770
C	-2.045235	4.081637	-1.081277
C	-4.049122	2.112154	-2.117978
C	-4.805447	3.720221	0.265527
C	0.132838	2.983156	1.353909
C	-2.392261	4.019046	2.693288
C	-1.226592	1.425728	3.557034
H	-1.531096	-5.052207	2.554538
H	-3.021411	-4.145478	2.891025
H	-2.809200	-4.960558	1.325076
H	0.201778	-2.957773	3.311784
H	0.150005	-1.303953	2.679360
H	-1.126719	-1.876274	3.776667
H	0.570390	-4.100443	0.681852
H	-0.544996	-3.870210	-0.683362
H	0.466270	-2.522694	-0.135483
H	-5.895754	-3.771002	-0.019684
H	-4.864542	-3.861586	1.427614
H	-5.872994	-2.426619	1.134722
H	-5.525530	-1.862497	-2.140324
H	-5.404929	-0.479068	-1.039929
H	-4.158022	-0.738684	-2.287061
H	-3.891040	-4.080270	-2.176573
H	-2.323014	-3.247394	-2.149570
H	-2.669696	-4.539049	-0.972363
H	-4.362587	2.976911	-2.723394
H	-3.325459	1.529911	-2.700189
H	-4.936400	1.491134	-1.947579
H	-5.253067	4.381828	-0.491519
H	-5.579147	3.002535	0.575146
H	-4.538896	4.328474	1.136849
H	-2.499105	4.710177	-1.861648
H	-1.697703	4.743462	-0.278161
H	-1.168424	3.585189	-1.521955
H	-1.767248	4.403234	3.513935
H	-2.495585	4.817935	1.948048
H	-3.388029	3.803760	3.105575
H	0.728664	3.392687	2.183001
H	0.665814	2.110337	0.943546
H	0.080800	3.738713	0.561882
H	-0.613130	2.033235	4.240001
H	-2.155631	1.168640	4.084973
H	-0.682617	0.496827	3.354400

105

PC (VI real)

C	-4.611560	-1.421756	-2.415689
C	-4.893466	-0.064692	-1.748771
C	-6.391634	0.249668	-1.854537
C	-4.084625	0.974772	-2.550522
P	-4.212740	-0.108353	0.040144
C	-4.725368	-1.740275	0.902298
C	-6.193354	-2.136181	0.697970
C	-4.861627	1.396996	1.031379
C	-6.319003	1.274964	1.494971
AU	-1.876670	-0.005379	-0.019737
C	0.162349	0.071015	-0.041346
O	0.871664	0.285430	-1.113221
AL	2.447771	0.110486	-0.048170
C	3.722740	-1.402021	-0.141326
SI	3.716894	-2.256798	1.555187
C	-3.940673	1.608115	2.249945
C	-4.711248	2.660118	0.166821
O	0.886673	-0.104944	1.027919
C	-3.805075	-2.867991	0.392047
C	-4.439673	-1.613282	2.408165
C	3.703372	1.637718	0.046761
SI	3.697316	2.489731	-1.651020
SI	3.325103	2.924235	1.384811
C	5.022292	0.824410	0.296849
C	5.033093	-0.573099	-0.383422
SI	3.368639	-2.693770	-1.481058
H	-4.245972	2.540441	2.748630
H	-3.997106	0.802853	2.985624
H	-2.892172	1.714864	1.938235
H	-4.920746	3.531140	0.805284

H	-3.688317	2.768093	-0.217712
H	-5.414395	2.691117	-0.671481
H	-6.602328	2.212119	1.997985
H	-7.013951	1.123097	0.661585
H	-6.461375	0.463140	2.216756
H	-4.576321	-2.606405	2.861148
H	-3.404649	-1.299807	2.598408
H	-5.121570	-0.924285	2.916589
H	-4.019102	-3.772898	0.980579
H	-3.956619	-3.112638	-0.661594
H	-2.746704	-2.609162	0.536494
H	-6.390671	-3.049373	1.279978
H	-6.889048	-1.364473	1.046068
H	-6.423107	-2.362810	-0.348952
H	-6.685378	0.187884	-2.913620
H	-7.011593	-0.460961	-1.296867
H	-6.630119	1.262037	-1.510193
H	-4.388971	0.900232	-3.605437
H	-4.254790	2.004084	-2.227282
H	-3.007034	0.768508	-2.491199
H	-4.846030	-1.325303	-3.486113
H	-3.553246	-1.702165	-2.331081
H	-5.229663	-2.231834	-2.016039
H	5.156048	0.674987	1.379032
H	5.914267	1.379105	-0.039886
H	5.929807	-1.116557	-0.040864
H	5.171998	-0.422187	-1.464790
C	4.243921	-4.361684	-1.206636
C	1.513071	-3.035244	-1.661824
C	3.974974	-2.183118	-3.201907
C	5.357147	-3.146009	1.891592
C	3.507399	-1.084225	3.029500
C	2.269979	-3.465317	1.747873
C	1.464286	3.244503	1.550400
C	3.920139	2.417179	3.110741
C	4.185822	4.600571	1.117693
C	2.242052	3.686348	-1.853483
C	5.331575	3.392398	-1.979517
C	3.503253	1.312100	-3.123089
H	3.985089	-5.031774	-2.040710
H	5.336050	-4.231482	-1.211423
H	3.968018	-4.866511	-0.272515
H	1.360169	-3.868125	-2.364435
H	1.035203	-3.302057	-0.710961
H	0.995110	-2.151943	-2.060198
H	3.839363	-3.048373	-3.868612
H	3.408809	-1.343702	-3.620726
H	5.042158	-1.920794	-3.215008
H	5.300001	-3.654015	2.866056
H	5.606075	-3.893310	1.128840
H	6.184927	-2.423982	1.944599
H	2.311954	-3.892012	2.761354
H	1.318575	-2.922371	1.652830
H	2.268508	-4.294927	1.031204
H	3.454451	-1.694181	3.944718
H	4.352348	-0.394345	3.140093
H	2.580351	-0.497986	2.971649
H	3.442637	1.918862	-4.039956
H	4.357352	0.633301	-3.231615
H	2.584173	0.713164	-3.064922
H	5.276451	3.900309	-2.954125
H	5.569304	4.141470	-1.214800
H	6.165909	2.677613	-2.027353
H	2.286327	4.110557	-2.867930
H	1.293812	3.137257	-1.761802
H	2.230954	4.518063	-1.139283
H	3.910960	5.269814	1.947250
H	5.279110	4.481308	1.134822
H	3.915457	5.100423	0.179287
H	1.295595	4.067709	2.260752
H	0.993094	3.517047	0.597810
H	0.952258	2.351213	1.934180
H	3.773388	3.281596	3.776171
H	3.354382	1.574511	3.523838
H	4.988412	2.160364	3.134062
